# Supplementary material for: Antibody agonists trigger immune receptor signaling through local exclusion of receptor-type protein tyrosine phosphatases
Source: Immunity. Author manuscript; Available in PMC 2026 Feb 24. (PMC7618792; doi:10.1016/j.immuni.2024.01.007)
Supplement: Supplementary Materials [file EMS212518-supplement-Supplementary_Materials.zip › 1-s2.0-S1074761324000335-mmc1.pdf]

Immunity, Volume *57*

Supplemental information

Antibody agonists trigger immune receptor signaling through local exclusion of receptor-type protein tyrosine phosphatases

Anna H. Lippert, Christopher Paluch, Meike Gaglioni, Mai T. Vuong, James McColl, Edward Jenkins, Martin Fellermeyer, Joseph Clarke, Sumana Sharma, Sara Moreira da Silva, Billur Akkaya, Consuelo Anzilotti, Sara H. Morgan, Claire F. Jessup, Markus Körbel, Uzi Gileadi, Judith Leitner, Rachel Knox, Mami Chirifu, Jiandong Huo, Susan Yu, Nicole Ashman, Yuan Lui, Ian Wilkinson, Kathrine E. Attfield, Lars Fugger, Nathan J. Robertson, Christopher J. Lynch, Lynne Murray, Peter Steinberger, Ana Mafalda Santos, Steven F. Lee, Richard J. Cornall, David Klenerman, and Simon J. Davis

SUPPLEMENTARY INFORMATION

Antibody agonists trigger immune receptor signaling through local exclusion of receptor-type protein tyrosine phosphatases

Anna H. Lippert, Christopher Paluch, Meike Gaglioni, Mai T. Vuong, James McColl, Edward Jenkins, Martin Fellermeyer, Joseph Clarke, Sumana Sharma, Sara Moreira da Silva, Billur Akkaya, Consuelo Anzilotti, Sara H. Morgan, Claire F. Jessup, Markus Körbel, Uzi Gileadi, Judith Leitner, Rachel Knox, Mami Chirifu, Jiandong Huo, Susan Yu, Nicole Ashman, Yuan Lui, Ian Wilkinson, Kathrine E. Attfield, Lars Fugger, Nathan J. Robertson, Christopher J. Lynch, Lynne Murray, Peter Steinberger, Ana Mafalda Santos, Steven F. Lee, Richard J. Cornall, David Klenerman, and Simon J. Davis

Figures S1-S7

Tables S1-S3

Supplementary figures

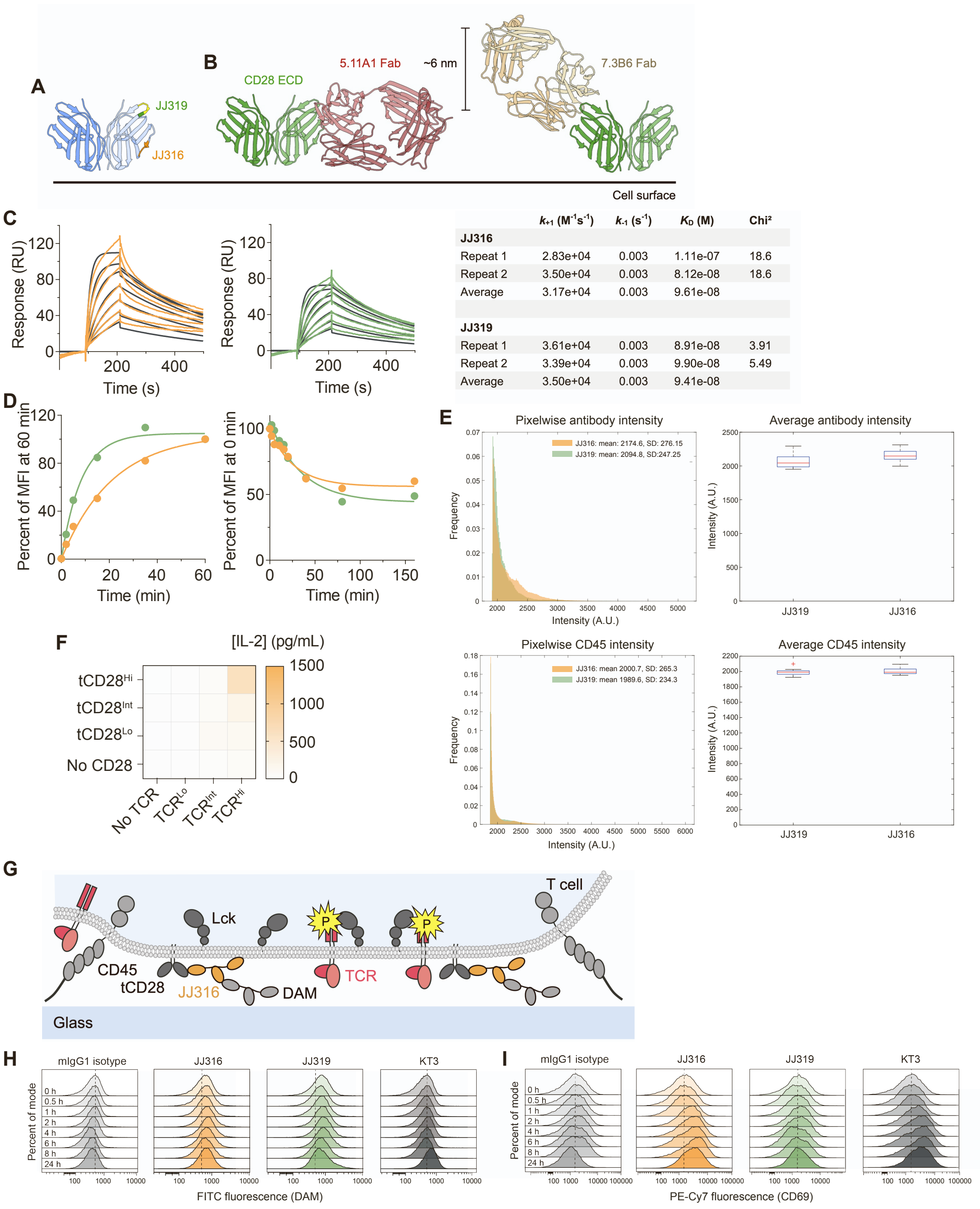

Figure S1 | Epitopes, binding properties, and signaling effects of anti-CD28 agonistic antibodies, related to Figure 1

(A) The positions of the JJ316 (orange) and JJ319 (green) anti-rCD28 antibody epitopes identified using mutational analysis by Luhder *et al.* [26], relative to the cell surface, shown on the structure of the hCD28 ECD. The JJ316 epitope was identified by substituting residues 60-65 of the C''D loop of mCD28 with the equivalent rat sequence, and the JJ319 epitope via a single substitution (of V98). For reference, the ligand binding MYPPPY motif is shown in yellow.

(B) Crystal structure of the complex of CD28 ECD with the Fab fragment of the 5.11A1 anti-hCD28 antibody, the murine precursor of TGN1412 (adapted from ref. [22]; left). Cryo-electron microscopy [22] showed that a non-mitogenic anti-hCD28 antibody, 7.3B6, bound to the "top" of the CD28 ECD, forming a ~6 nm "taller" complex (measured along an axis orthogonal to the membrane; right).

(C) Example sensorgrams and summary of the affinity and kinetic properties for the binding of Fab fragments of JJ316 and JJ319 antibodies to immobilized rCD28 ECD expressed in the form of a fusion protein with mIgG1 Fc. Measurements were done at 37 °C using a Biacore 3000 machine; the ligand immobilization level was ~300 RU. Analyte concentrations varied from 9.33×10^{-8} to 1.2×10^{-6} M. Black lines in the sensorgrams represent fits of a 1:1 Langmuir binding model to the data.

(D) Flow cytometry-based analysis of the binding of Alexa Fluor 647 labeled antibodies to CD28-expressing BW cells at 37 °C. Data represent averages of three independent repeats. The curves on the left were fitted with an association model: MFI = MFI_{max} * (1-exp(-1*(k_{+1}*L+k_{-1})*time)), where L is the antibody concentration (in M). The curves on the right were fitted with a dissociation model: MFI = (MFI_0 – NS) * exp(-k_{-1}*time) + NS, where NS is non-specific binding at infinite times. The association rates for JJ316 and JJ319 binding to CD28 (3.3×10^5 and 2.9×10^6 $M^{-1}s^{-1}$, respectively) were significantly different (p = 0.0003; F-test) but not the dissociation rates (3.67×10^{-2} s^{-1} and 2.84×10^{-2} s^{-1}; p = 0.3578). Curve fitting was done in GraphPad Prism.

(E) Antibody and Fab labeling levels. Pixelwise and per-cell averaged fluorescence intensities greater than background + 2 SD, for TCR-deficient $tCD28^+$ BW cells incubated with Alexa Fluor 647 labeled JJ316 (orange) or JJ319 (green) antibody, and Alexa Fluor 488 tagged anti-CD45 Fab. Images were taken at the mid-plane of the cells under epi-illumination, following a 15' incubation with the fluorescent antibodies or Fab. Degree of labeling (dyes/molecule) was 6.9 (JJ316) and 6.4 (JJ319); N = 19 cells (JJ316) and 20 cells (JJ319). Boxes indicate the 25% and 75% quartile, the red horizontal line the median, and whiskers the 1.5 x IQR.

(F) Effects of varying the expression of the TCR and tCD28, a non-signaling form of CD28 lacking its cytosolic region, on signaling by BW cells induced by JJ316 antibody (10 µg/ml).

(G) Proposed mechanism of signaling induced by antibody binding to tCD28 expressed in the presence of high levels of TCR. The formation of a large contact by the binding of immobilized JJ316 to tCD28 leads to exclusion of CD45 over a large area, greatly reducing the likelihood that TCRs constitutively phosphorylated by kinases will be exposed to phosphatases, e.g., CD45, that would otherwise quench signaling.

(H) Time course for staining of cells for DAM adsorption from DAM antibody-coated surfaces during culture for 24 h on these surfaces in the presence of the indicated antibodies at 10 µg/mL, using a FITC-labeled rabbit anti-donkey IgG antibody. The dashed line corresponds to the peak-level staining in the presence of the mIgG1 isotype control.

(I) Staining of cells for CD69 expression following a 24 h culture as in (H), using a PE-Cy7-labeled anti-mCD69 antibody.

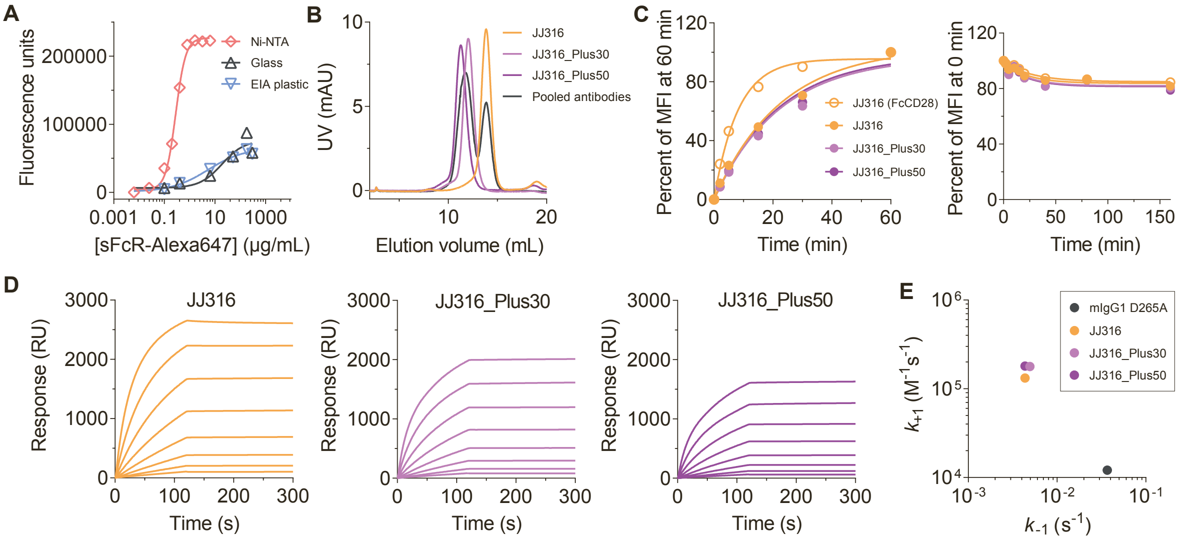

Figure S2 | High-level antibody immobilization and characterization of the effects of extending mIgG1 antibodies with mucin-like sequence, related to Figure 2

(A) Ni-NTA plastic immobilizes His-tagged proteins at very high levels. Histidine (6x)-tagged mFcγR2b ECD was labeled with Alexa Fluor 647 and then allowed to bind to Ni-NTA-coated plastic, or anti-FcR antibody-coated EIA plastic or glass. Fluorescence at 647 nm was used to determine the amount of binding.

(B) Thirty (“Plus30”) or 50 (“Plus50”) residues of mucin-like sequence from the mCD43 ECD were inserted into the hinge regions of JJ316 antibody. Fifty micrograms of unmodified JJ316, JJ316_Plus30, and JJ316_Plus50 were subjected to size exclusion chromatography on a Sepharose S200 column, either alone or as a mixture.

(C) Flow cytometry was used to follow the binding of the native and hinge-extended Alexa Fluor 647 labeled antibodies to CD28 and FcCD28-expressing BW cells at 37 °C. Data represent averages of three independent repeats. Curves were fitted as in Fig. S1D.

(D) Example surface plasmon resonance-derived sensorgrams showing the binding of JJ316 and extended variants to immobilized rCD28 ECD expressed in the form of a fusion protein with mIgG1 Fc. Measurements were done at 37 °C; ligand immobilization levels were ~3000 RU. Analyte concentrations varied from 1 x 10^{-1} to 7.8 x 10^{-4} mg/ml.

(E) Kinetic analysis of JJ316 and hinge-extended antibody binding to mFcγR2b ECD. Measurements were done at 37 °C; antibodies were immobilized at ~2250 RU. Analyte concentrations varied from 1.60 x 10^{-2} to 6.70 x 10^{-5} mg/ml. Biacore Evaluation Software was used to fit a 1:1 binding model. Calculated on- and off-rates are shown, compared to MOPC-21 mIgG1 D265A used as a negative control.

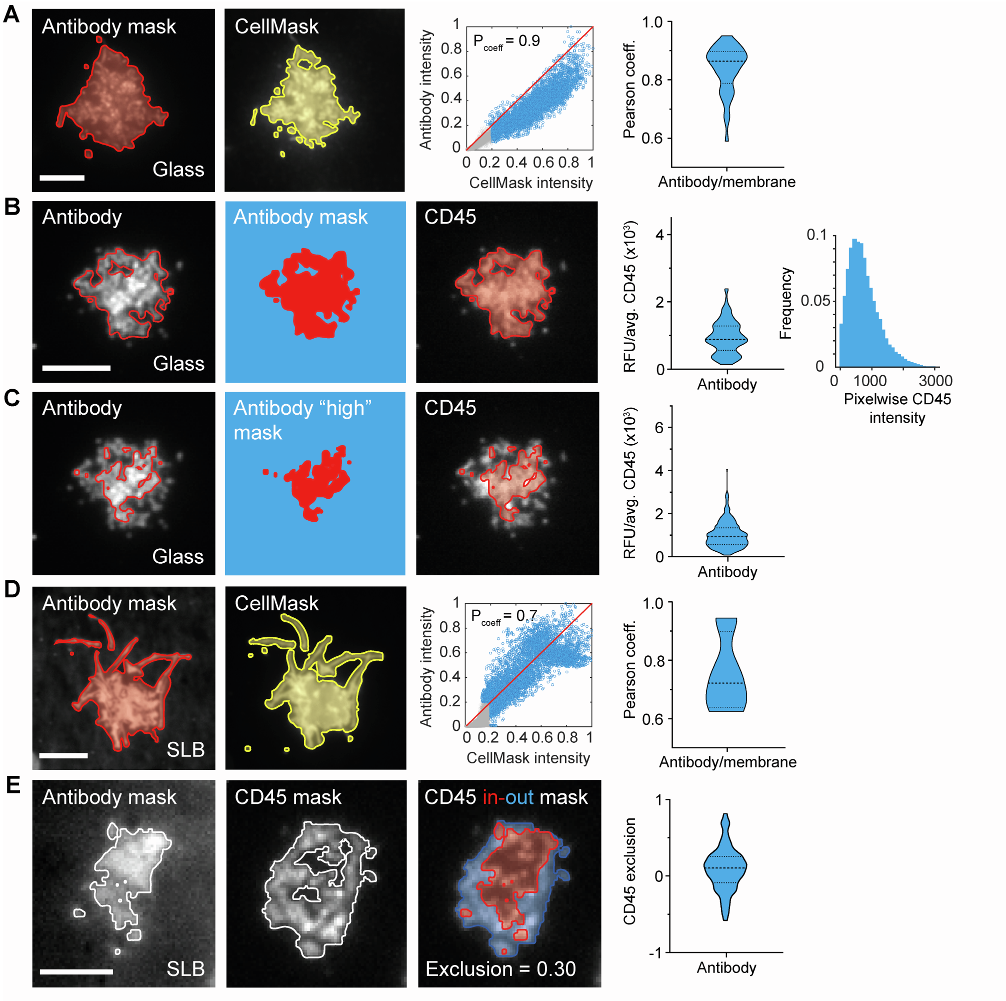

Figure S3 | Fluorescence intensity analysis, related to Figure 3

(A) TIRF images of antibody-treated cells interacting with DAM-coated glass coverslips, following staining with fluorescent anti-CD28 antibody (red) and CellMask (yellow; left panels). Relationship between the pixelwise fluorescence intensities in the antibody and CellMask channels, and correlation calculated using Pearson's coefficient and plotted as a violin plot (right panels; with the violin plot showing 25% and 75% quantiles (dotted lines) and median (dashed line)).

(B) TIRF images of antibody fluorescence for antibody-treated cells interacting with DAM-coated glass coverslips were intensity-thresholded (40% of max. intensity, left panel) to create antibody masks (red, middle panel). These masks were overlayed onto CD45 fluorescence images (right panel) allowing measurements of the average CD45 fluorescence intensity across regions delineated by the antibody masks, presented as violin plots and as histograms of the pixelwise CD45 fluorescence intensities.

(C) Intensity thresholding (60% of max. intensity) was used to also generate masks delineating regions of "high" antibody fluorescence, allowing levels of CD45 fluorescence in these regions to be determined.

(D) Correlation between the pixelwise fluorescence intensities in antibody (red) and CellMask (yellow) channels, for cells interacting with SLBs.

(E) Antibody (40% max. intensity) and CD45 (20% max. intensity) masks (white), for antibody-treated cells interacting with SLBs, were generated by intensity thresholding and overlayed, creating two masks allowing CD45 intensities inside ($CD45_{in}$, red) and outside ($CD45_{out}$, blue) regions of high antibody fluorescence to be measured. Violin plots were used to present cell-wise "exclusion" values (Exclusion = 1 – (Avg $CD45_{in}$/Avg $CD45_{out}$).

Scale bars, 5 µm.

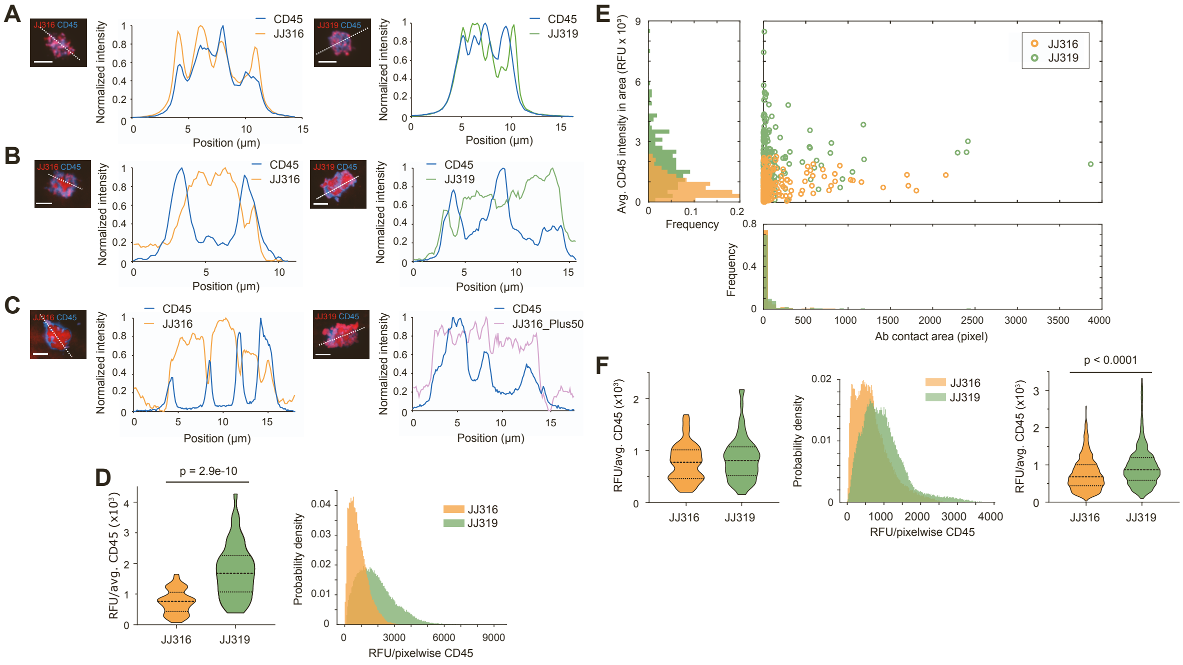

Figure S4 | Fluorescence imaging-based analysis of antibody and CD45 redistribution, related to Figure 3

(A-C) Line scans showing normalized antibody and CD45 fluorescence intensity values from Figure 3B,K, and L in the regions indicated by the dotted lines in TIRF images of tCD28^{+} BW cells treated with Alexa Fluor 647 tagged JJ316, JJ319, or JJ316_Plus50 antibody (red) and Alexa Fluor 488 labeled anti-CD45 Fab fragments (blue). The cells were interacting with DAM-coated coverslips (A) or FcR-antibody presenting SLBs (B,C). Scale bars, 5 µm.

(D) CD45 distribution analyzed using Otsu-based thresholding. Antibody and CD45 masks were created for TIRF images of JJ316- and JJ319-treated tCD28^{+} BW cells interacting with DAM-coated glass surfaces using the Otsu method. Violin plots (left panel) show cell-wise average CD45 intensity values; dotted lines indicate the 25% and 75% quartile and the dashed line the median. The histogram (right panel) shows the probability density function for the pixelwise CD45 intensity for all cells.

(E) Contact size and CD45 exclusion. The scatter plot and histograms show contact sizes and corresponding average CD45 fluorescence intensities for JJ316 (orange)- and JJ319 (green)-interacting tCD28-expressing BW cells.

(F) CD45 distribution for BW cells expressing tCD28 at levels corresponding to that of CD28 expressed by TCR^{Int}-CD28^{Int} BW cells (see Fig. 1F), treated with JJ316 and JJ319 antibody. Violin plots (left panel) compare the average CD45 fluorescence intensities for individual cells (N = 76 cells (JJ316), N = 61 cells (JJ319)). The probability density functions (middle panel) show the pixelwise CD45 fluorescence intensity for all cells. Violin plots (right panel) show the average CD45 intensities in regions of antibody fluorescence. Dotted lines indicate the 25% and 75% quartile and the dashed line the median.

In (D) and (F) two-sample Student's t-tests were used for statistical comparisons.

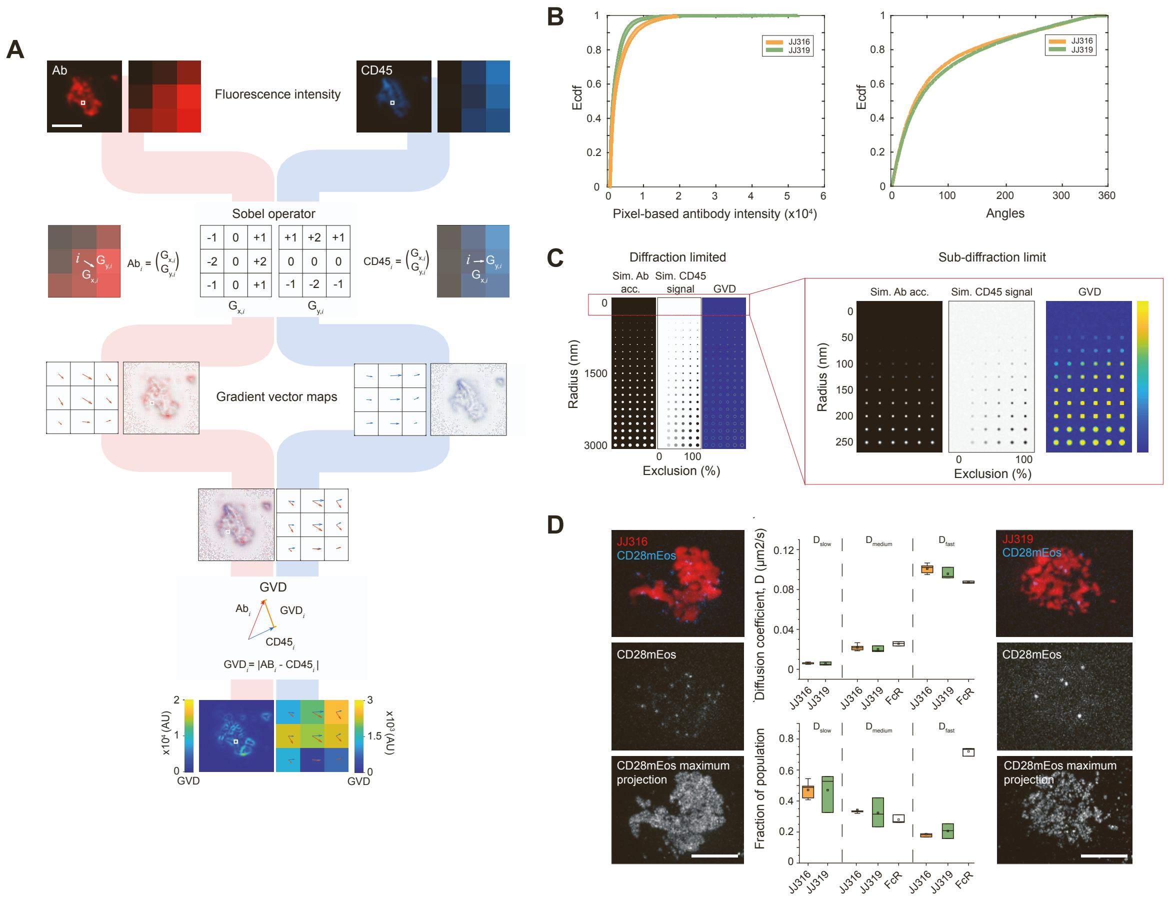

Figure S5 | Gradient vector distance and diffusion analyses, related to Figure 3

(A) For GVD analysis, the CD45 and antibody channels (top) were multiplied with the Sobel operator generating directional gradient vectors Gx and Gy for each pixel (middle). These gradient vector maps were then subtracted from each other, and the GVDs produced were color-coded (bottom). In the top panels, the raw data for each fluorescence channel is shown (left), alongside a subset of 9 pixels (right). For comparisons between two conditions, the fluorescence intensities in the antibody channel were divided by the degree of labeling of the antibody, in order to correct for differences. Scale bar, 5µm.

(B) Sources of differences in the GVD analysis shown in Fig. 3I. The panel on the left shows the empirical cumulative distribution function (Ecdf) for antibody fluorescence intensity, i.e., the pixelwise intensity for values greater than 1.5 x background for antibody intensities measured for JJ316- (orange) and JJ319- (green) treated tCD28^{+} BW cells. The right-hand panel shows the Ecdf of the angles between the vectors assigned to the intensity gradients in the antibody and CD45 fluorescence channels. Degrees of labeling of the antibodies (dyes/molecule) were 9.1 (JJ316) and 7.1 (JJ319).

(C) Simulations show that GVD analysis allows detection of molecular exclusion on sub diffraction-limited length scales. Simulations of antibody accumulation and varying CD45 exclusion, and the resulting GVD plots are shown. The sizes of the contacts and levels of CD45 exclusion were varied. For large regions of accumulation and exclusion, the GVD analysis functioned as an edge detector whereas at sub diffraction-limited length scales, the GVD plot highlights regions of local CD45 versus antibody exclusion. Signal-to-noise ratios were matched to the data shown in Fig. 3I. The simulations were generated on a 16 nm grid assuming homogeneous fluorescence with contacts excluding (CD45) or accumulating (antibody) signal, Gaussian-blurred with a sigma of 131 nm to simulate image formation, and downsampled to a pixel size of 160 nm to match the original data.

(D) CD28 diffusion analysis. BW cells expressing mEos-tagged tCD28 were allowed to settle onto mFcγR2b ECD-presenting bilayers which were preloaded with Alexa Fluor 647 labeled JJ316 or JJ319 antibody. Fluorescence images show antibody fluorescence (red, top) overlayed with a single frame of diffusing CD28mEos (blue, middle) and CD28mEos maximum projection (bottom). Scale bars, 5 µm. Boxplots show diffusion coefficients (upper) and fractions (lower) of slow, medium and fast diffusing populations on antibody presenting and FcR-only bilayers. Data correspond to ~5000 tracks taken from 10 cells each (JJ316- and JJ319-presenting SLBs), or 6 cells (FcR-only SLBs). Boxes indicate the 25% and 75% quartile, horizontal line the median, the square the mean, and whiskers the 1.5 x IQR.

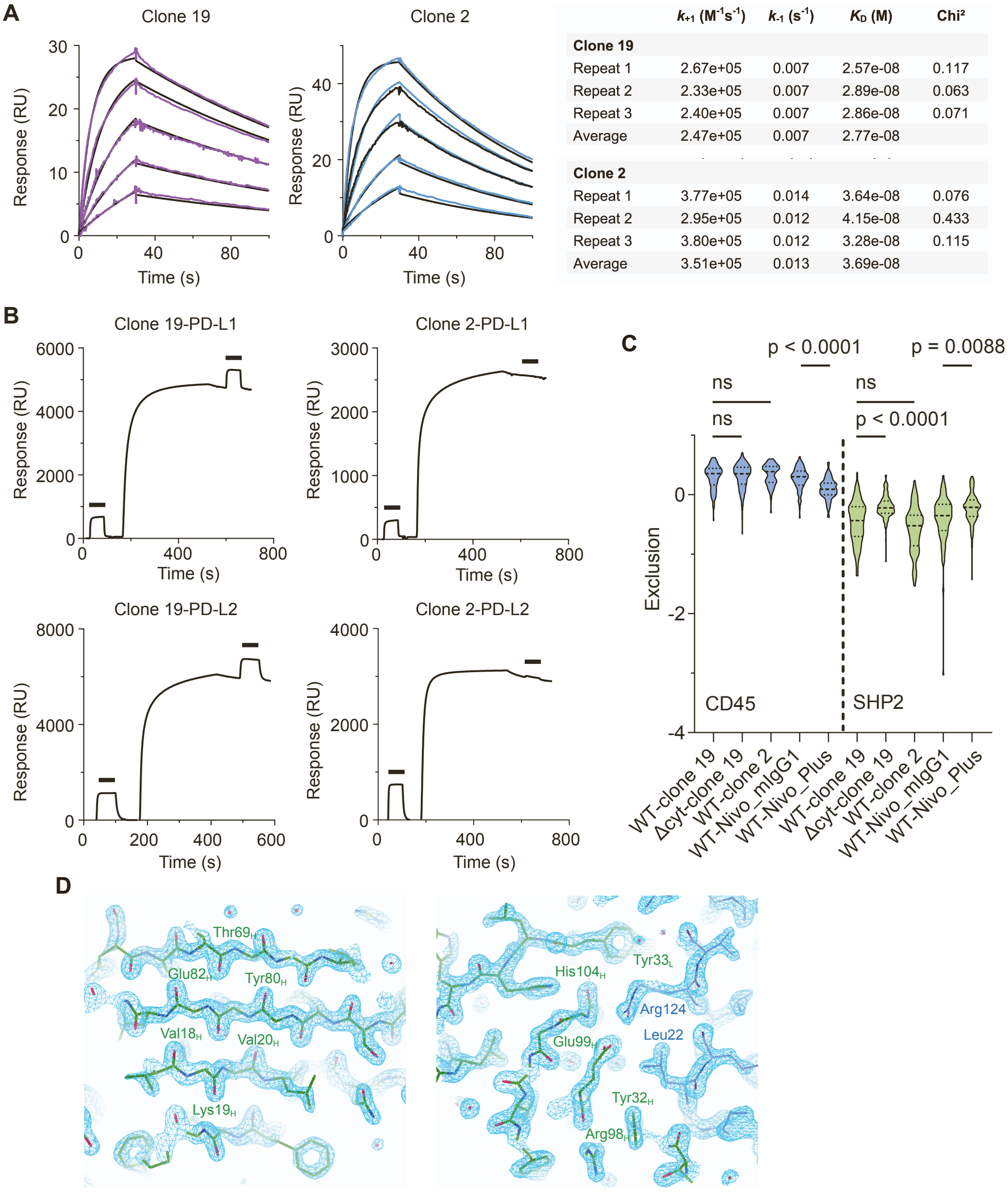

	k_{+1} ($M^{-1}s^{-1}$)	k_{-1} (s^{-1})	K_D (M)	Chi²
**Clone 19**				
Repeat 1	2.67e+05	0.007	2.57e-08	0.117
Repeat 2	2.33e+05	0.007	2.89e-08	0.063
Repeat 3	2.40e+05	0.007	2.86e-08	0.071
Average	2.47e+05	0.007	2.77e-08	
**Clone 2**				
Repeat 1	3.77e+05	0.014	3.64e-08	0.076
Repeat 2	2.95e+05	0.012	4.15e-08	0.433
Repeat 3	3.80e+05	0.012	3.28e-08	0.115
Average	3.51e+05	0.013	3.69e-08	

Figure S6 | Anti-PD-1 antibody binding to PD-1, related to Figures 4 and 5
(A) Surface plasmon resonance-based analyses of the affinity of binding of soluble, monomeric PD-1 to directly immobilized clone 19 and clone 2.
(B) Analysis of the blocking effects of the anti-PD-1 antibodies. In the blocking experiment, PD-1Fc was directly immobilized to a Biacore T200 sensor chip, into which the indicated ligands, PD-L1 or PD-L2 (0.35 mg/ml), were injected (first black bar) in order to establish the levels of ligand binding before addition of the anti-PD-1 antibodies. Note that the ligands completely dissociated before injection of the antibodies. The clone 19 or clone 2 antibodies (0.1 mg/ml) were then injected into the sensor chip, giving large, close-to-saturating amounts of binding. In a final step, the ligands were re-injected (0.35 mg/ml; second black bar) in order to observe the amounts of PD-L1 and PD-L2 binding in the presence of the antibodies. The absence of an increase in signal during the second ligand injection step indicated that clone 2, but not clone 19, blocked ligand binding to PD-1.

(C) Violin plots of CD45 (blue) and SHP2 (green) mask-based exclusion values comparing different antibody-mediated contacts: WT-clone 19 (N = 101 cells), WT-clone 2 (N = 110 cells), WT-Nivo_mIgG1 (N = 78 cells), WT-Nivo_Plus (N = 83 cells), and PD-1Δcyt-clone 19 (N = 122 cells), for cells expressing either PD-1WT (WT) or PD-1Δcyt (Δcyt), on bilayers comprising 95% POPC, 5% DGS-NTA(Ni), rather than 98% POPC, 2% DGS-NTA(Ni) as used in the experiments shown in Fig. 5, giving higher levels of antibody immobilization. The Kruskal-Wallis test with Dunn's multiple comparison follow-up testing was used to compare each group to the WT control.
(D) Representative regions of electron density used in modeling the clone 19 Fab-PD-1 structure. Section of the V_H DEBA β-sheet of the clone 19 Fab (left panel), and PD-1 (blue) and clone 19 Fab (green) interface resides (right panel; mature polypeptide numbering). The $2F_{obs}–F_{calc}$ maps (blue mesh) were contoured at 0.7 sigma.

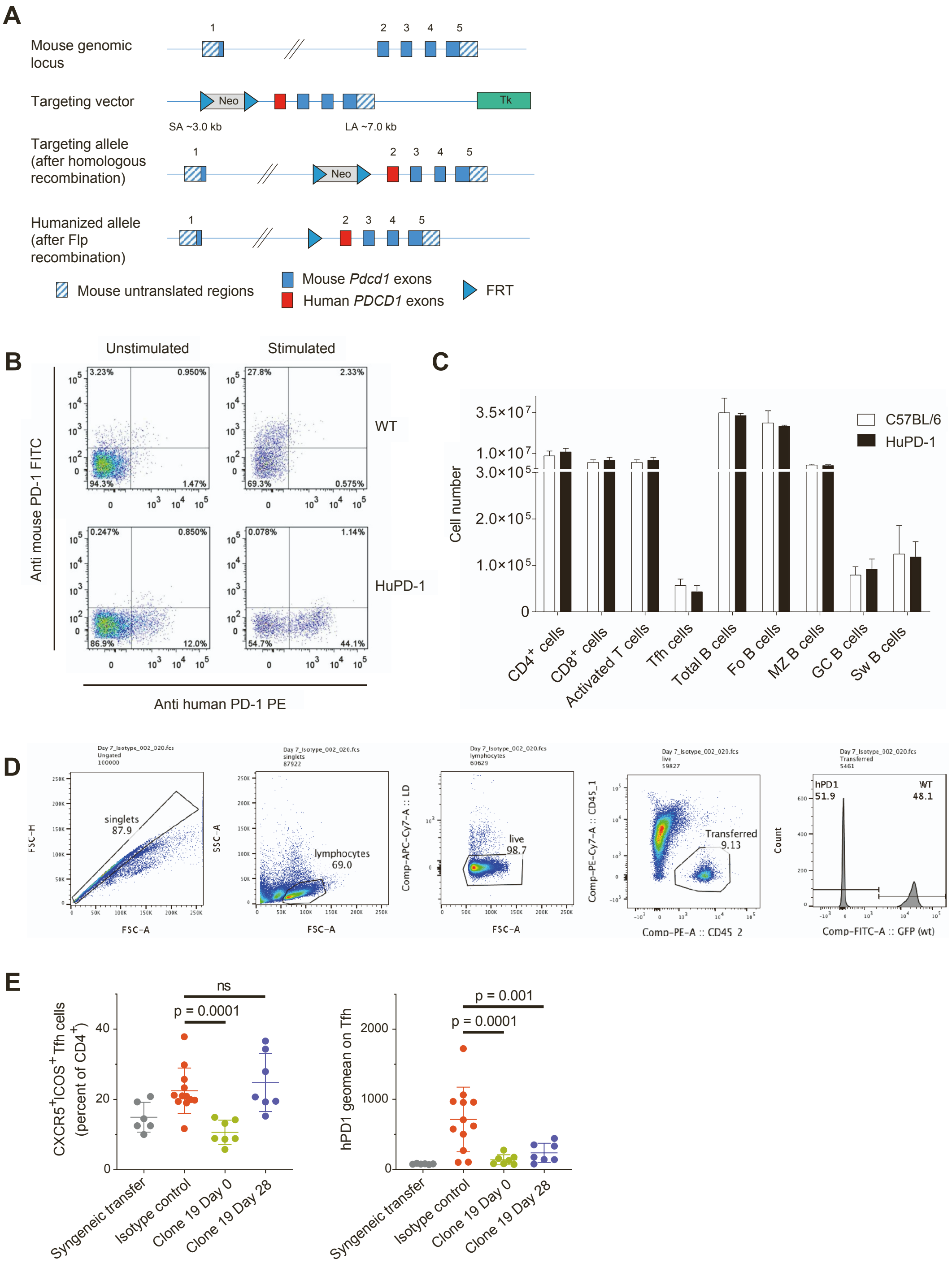

Figure S7 | In vivo experiments, related to Figure 6

(A) Strategy for generation of the humanized allele in huPD-1 mice (adapted from ref 40). Murine *Pdcd1* exon 2, encoding the ECD of PD-1, was replaced by the human counterpart using targeted homologous recombination by electroporation of mouse C57BL/6N ES cells. Microinjection of blastocysts with positive ES clones was performed with implantation of blastocysts into foster mice. Chimeric offspring were bred to FLP transgenic mice and offspring with germline transmission of the knockin, plus somatic deletion of the frt flanked neomycin resistance cassette, were identified. Mice were bred to homozygosity of the humanized allele, with genotyping performed by PCR of digested ear punch samples with primers specific for *PDCD1* (human) or *Pdcd1* (mouse) exon 2.

(B) Flow cytometry plots showing upregulation of the humanized PD-1 protein on T cells upon in vitro stimulation. Splenocytes from WT C57BL/6 mice and homozygous huPD-1 mice were stimulated with 1 µg/ml anti-CD3 antibody. Cells were stained with FITC conjugated anti-mPD-1 and PE conjugated anti-human PD-1 antibodies. Weak expression of PD-1 by splenic T cells in the resting state (versus, e.g., PBMCs), was expected.
(C) Comparison of the naïve status of huPD-1 and C57BL/6 immune systems. Quantification of immune cell subsets in spleen by flow cytometry, gating for specific populations, was as follows: activated T cells ($CD3^+CD44^+ICOS^+$), Tfh ($CD4^+ICOS^+CXCR5^+$), Fo B cells ($B220^+CD23^{hi}CD21^{int}$), MZ B cells ($B220^+CD23^{lo}CD21^{hi}$), GC B cells ($B220^+CD95^+GL7^+$), and Sw B cells ($B220^+IgG^+$). Data were pooled from three independent experiments with at least two mice per group (mean+/-SD).
(D) Flow cytometry gating strategy for the OT-II adoptive transfer study. Transferred versus recipient cells were separated based on the CD45 allelic marker, and huPD-1 versus WT PD-1 expressing cells within the transferred population were separated based on GFP expression.
(E) Evidence that clone 19 IgG1 is a non-depleting antibody. Fraction of $CD4^+$ T cells expressing CXCR5 and ICOS, i.e., Tfh cells, in mice on Day 30 following treatments on Days 0 or 28 with 10 mg/kg clone 19 IgG1 anti-PD-1 or isotype control antibody, in the setting of the SLE model (left panel, see Fig. 6). Levels of expression of PD-1 by the Tfh cells (right panel). Treatment with clone 19 for 2 days, i.e., the time during which depletion by CDC-ADCC would occur, leads to downregulation of PD-1 only, confirming that the receptor has been engaged and that the antibody is non-depleting. Each point represents an individual mouse. Error bars shown represent SD. Data are representative of two independent experiments. One-way ANOVA with Dunnett's multiple comparison follow-up testing comparing each group to the isotype control, was used for statistical comparisons.

Supplementary tables

Table S1 | Non-linear regression/binding model*-based analysis of the agonistic and costimulatory activities of JJ316 and JJ319 antibodies, related to Figures 1 and 2

	No anti-CD28	Isotype	JJ316	JJ319
**DO11.10**				
**Maximum**	Constrained	Constrained	Constrained	Constrained
**n**	0.9894	0.945	0.8109	0.8006
**EC50 (µg/mL)**	28.64	32.25	213.8	1.492****
**Minimum**	-9.275	-6.292	654.4†	22.4
**Yae5b3k**				
**Maximum**	Constrained	Constrained	Constrained	Constrained
**n**	1.383	1.301	0.8145	0.6415
**EC50 (µg/mL)**	29.82	20.67	66.94	2.762**
**Minimum**	-2.176	1.455	202.1***	9.15
**TCR^+ BW**				
**Maximum**	722.9	890.6	309.4	833.3
**n**	0.8151	0.4672	2.398	3.553
**EC50 (µg/mL)**	1.888	3.312	0.008186	0.009791‡
**Minimum**	-2.135	-28.46	529.7§	6.886

* Model: [IL-2] = Maximum * [KT3]n/(EC50n + [KT3]n) + Minimum; where Maximum = [IL-2] at maximal [KT3]; EC50 = [KT3] that gives half-maximal [IL-2]; n = Hill slope; Minimum = [IL-2] at [KT3] = 0
** Different from isotype, p = 0.0023 (F-test)
*** Different from isotype, p < 0.0001 (F-test)
**** Different from isotype, p < 0.0001 (F-test)
† Different from isotype, p < 0.0001 (F-test)
‡ Different from isotype, p < 0.0001 (F-test)
§ Different from isotype, p = 0.0002 (F-test)

Table S2 | Data collection and refinement statistics for the PD-1-clone 19 Fab crystal structure, related to Figure 5

Space group	P 1
Unit cell	a = 52.16 Å, b = 53.79 Å, c = 103.00 Å, α = 104.80°, β = 101.83°, γ = 92.57°
Wavelength (Å)	0.979499
Resolution range (Å)	51.85 - 2.03 (2.06 - 2.03)
Completeness (%)	98.03 (95.11)
Multiplicity	3.63 (3.71)
CC-half	0.9918 (0.2220)
I/sigma	11.11 (1.07)
Rmerge(I)	0.1322 (1.2855)
Anomalous completeness (%)	96.89 (92.69)
Anomalous multiplicity	1.82 (1.88)
Reflections used in refinement	66773 (6591)
Reflections used for R-free	3086 (304)
R-work	0.2141 (0.3314)
R-free	0.2725 (0.3511)
Number of non-hydrogen atoms	8443
Macromolecules	8166
Ligands	28
Solvent	249
Protein residues	1058
RMS (bonds)	0.015
RMS (angles)	2.08
Ramachandran favored (%)	96.13
Ramachandran allowed (%)	3.29
Ramachandran outliers (%)	0.58
Rotamer outliers (%)	6.80
Clash score	6.44
Average B-factor	35.84
Macromolecules	35.97
Ligands	61.24
Solvent	28.84

Table S3 | Comparison of the interfaces formed with PD-1 by anti-PD-1 antibodies revealed by crystal structural studies, related to Figure 5

		**Clone 19** (PDB: 8eq6)	**Nivolumab** (PDB: 5WT9)	**Pembrolizumab** (PDB: 5B8C)
**Buried area (Å^2)**	V_L	288.7	290.6	551.8
	V_H	484.2	571.3	662.1
	PD-1	781.5	926	1170.9
**No. of contacting residues**	V_L	5	4	11
	V_H	11	12	16
	PD-1	15	11	23
**No. of charged contacting residues**	V_L	0	0	2
	V_H	3	3	2
	PD-1	5	3	6
**No. of polar contacting residues**	V_L	5	2	7
	V_H	8	11	11
	PD-1	10	6	13
**No. of hydrophobic contacting residues**	V_L	3	4	8
	V_H	9	6	11
	PD-1	8	5	11
**No. of aromatic contacting residues**	V_L	3	1	4
	V_H	5	3	4
	PD-1	0	0	2
**Shape complementarity *(Sc)*[1]**		0.72	0.81	0.68

Supplementary reference

1. Lawrence, M.C., and Colman, P.M. (1993) Shape complementarity at protein/protein interfaces. J. Mol. Biol. *234*, 946-950.
