# Supplementary material for: Antibody agonists trigger immune receptor signaling through local exclusion of receptor-type protein tyrosine phosphatases
Source: Immunity. Author manuscript; Available in PMC 2026 Feb 24. (PMC7618792; doi:10.1016/j.immuni.2024.01.007)
Supplement: Supplementary Materials [file EMS212518-supplement-Supplementary_Materials.zip › 1-s2.0-S1074761324000335-mmc6.pdf]

Immunity

Article

Antibody agonists trigger immune receptor signaling through local exclusion of receptor-type protein tyrosine phosphatases

Graphical abstract

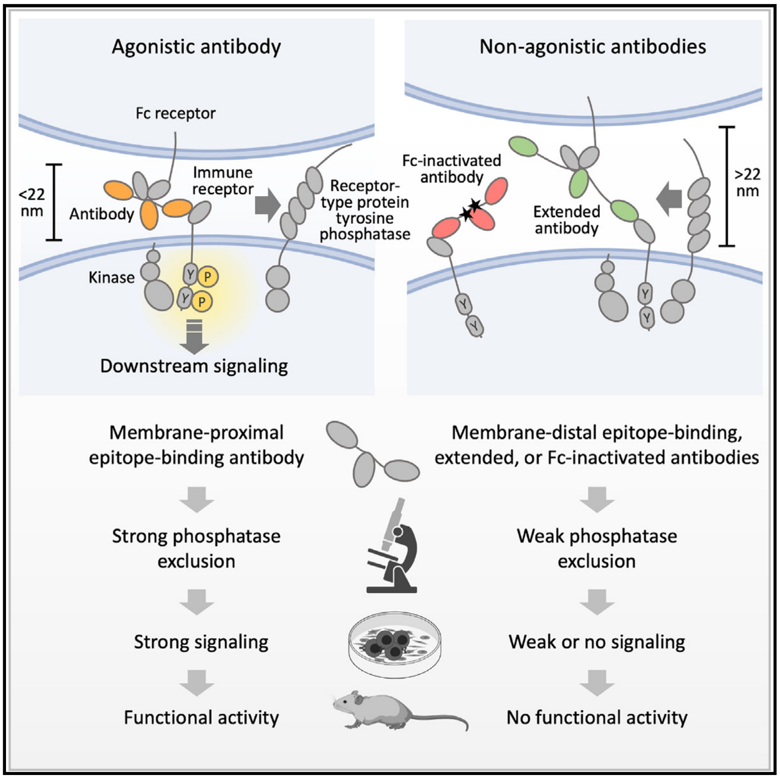

Highlights

- Local, steric phosphatase exclusion by antibodies drives immune receptor signaling
- Agonists targeting activating and inhibitory receptors can be rationally designed
- A bespoke PD-1 agonist exemplifies a new class of treatments for autoimmunity
- Antibody engineering that limits agonism improves immune checkpoint receptor blockade

Authors

Anna H. Lippert, Christopher Paluch, Meike Gaglioni, ..., Richard J. Cornall, David Klenerman, Simon J. Davis

Correspondence

richard.cornall@ndm.ox.ac.uk (R.J.C.),
dk10012@cam.ac.uk (D.K.),
simon.davis@imm.ox.ac.uk (S.J.D.)

In brief

Antibodies can initiate immune receptor signaling. Lippert et al. provide mechanistic insight into this effect, showing that antibody agonists trigger receptor signaling by locally excluding receptor-type protein tyrosine phosphatases from sites of antibody-receptor engagement. This finding provides a framework for the engineering of therapeutic antibodies.

Lippert et al., 2024, Immunity 57, 256–270
February 13, 2024 © 2024 The Author(s). Published by Elsevier Inc.
https://doi.org/10.1016/j.immuni.2024.01.007

CellPress
OPEN ACCESS

Immunity

Article

Antibody agonists trigger immune receptor signaling through local exclusion of receptor-type protein tyrosine phosphatases

Anna H. Lippert,[1,9] Christopher Paluch,[2,3,4,5,9] Meike Gaglioni,[2,3,9] Mai T. Vuong,[2,3,9] James McColl,[1,9] Edward Jenkins,[2,3] Martin Fellermeyer,[2,3] Joseph Clarke,[2,3] Sumana Sharma,[2,3] Sara Moreira da Silva,[5] Billur Akkaya,[2,3,4] Consuelo Anzilotti,[2,3,4] Sara H. Morgan,[2,3] Claire F. Jessup,[2,3] Markus Körbel,[1] Uzi Gileadi,[2] Judith Leitner,[6] Rachel Knox,[2,3] Mami Chirifu,[2,3] Jiandong Huo,[2,3] Susan Yu,[2,3] Nicole Ashman,[2,3] Yuan Lui,[2,3] Ian Wilkinson,[7] Kathrine E. Attfield,[2,8] Lars Fugger,[2,8] Nathan J. Robertson,[5] Christopher J. Lynch,[5] Lynne Murray,[5] Peter Steinberger,[6] Ana Mafalda Santos,[2,3] Steven F. Lee,[1] Richard J. Cornall,[2,4,*] David Klenerman,[1,*] and Simon J. Davis[2,3,10,*]
[1]Department of Chemistry, University of Cambridge, Cambridge, UK
[2]MRC Human Immunology Unit, John Radcliffe Hospital, University of Oxford, Oxford, UK
[3]Radcliffe Department of Medicine, John Radcliffe Hospital, University of Oxford, Oxford, UK
[4]Nuffield Department of Medicine, University of Oxford, Oxford, UK
[5]MiroBio Ltd, Winchester House, Oxford Science Park, Oxford, UK
[6]Division of Immune Receptors and T cell Activation, Institute of Immunology, Medical University of Vienna, Vienna, Austria
[7]Absolute Antibody Ltd, Redcar, Cleveland, UK
[8]Oxford Centre for Neuroinflammation, Nuffield Department of Clinical Neurosciences, John Radcliffe Hospital, University of Oxford, Oxford, UK
[9]These authors contributed equally
[10]Lead contact
*Correspondence: richard.cornall@ndm.ox.ac.uk (R.J.C.), dk10012@cam.ac.uk (D.K.), simon.davis@imm.ox.ac.uk (S.J.D.)
https://doi.org/10.1016/j.immuni.2024.01.007

SUMMARY

Antibodies can block immune receptor engagement or trigger the receptor machinery to initiate signaling. We hypothesized that antibody agonists trigger signaling by sterically excluding large receptor-type protein tyrosine phosphatases (RPTPs) such as CD45 from sites of receptor engagement. An agonist targeting the costimulatory receptor CD28 produced signals that depended on antibody immobilization and were sensitive to the sizes of the receptor, the RPTPs, and the antibody itself. Although both the agonist and a non-agonistic anti-CD28 antibody locally excluded CD45, the agonistic antibody was more effective. An anti-PD-1 antibody that bound membrane proximally excluded CD45, triggered Src homology 2 domain-containing phosphatase 2 recruitment, and suppressed systemic lupus erythematosus and delayed-type hypersensitivity in experimental models. Paradoxically, nivolumab and pembrolizumab, anti-PD-1-blocking antibodies used clinically, also excluded CD45 and were agonistic in certain settings. Reducing these agonistic effects using antibody engineering improved PD-1 blockade. These findings establish a framework for developing new and improved therapies for autoimmunity and cancer.

INTRODUCTION

Decisions leading to lymphocyte activation or suppression depend not just on antigen recognition by antigen receptors but also on other, related receptors that tune the cells to vital cues in their environment.[1,2] Collectively, these "immune receptors" have (1) small extracellular domains (ECDs; <20 nm) that bind ligands anchored to the surfaces of other cells, and (2) unstructured cytosolic regions with multiple tyrosine residues that are phosphorylated and dephosphorylated by membrane-associated, extrinsic tyrosine kinases, e.g., Lck, and large receptor-type protein tyrosine phosphatases (RPTPs), such as CD45, respectively.[3] The use of antibodies to block signaling by the inhibitory immune receptors PD-1 and CTLA-4, which enhances T cell activation, has transformed cancer treatment, producing durable remissions in otherwise refractory disease.[4–7] As many as 100 immune receptors regulate immunity,[3] suggesting that there is considerable scope for modulating immune responses by targeting these receptors.

Antibodies can also be very potent activators of receptor signaling[8,9] as revealed during the phase I clinical trial of an antibody agonist (TGN1412) that bound the activating immune receptor, CD28, which resulted in cytokine-release syndrome, severe lymphopenia, and life-threatening multi-organ failure in trial volunteers.[10] More promisingly, an agonist targeting the related costimulatory receptor ICOS has robust anti-tumor activity in

 © 2024 The Author(s). Published by Elsevier Inc.
This is an open access article under the CC BY license (http://creativecommons.org/licenses/by/4.0/).

CellPress
OPEN ACCESS

preclinical models and a favorable safety profile.[11] Similarly, inhibitory agonists generated against immune checkpoints are effective in animal models of autoimmunity[12] and, accordingly, are now beginning to enter the clinic.[13] However, there has been no framework for systematically identifying antibody agonists, or optimizing their signaling potential in therapeutic settings where it would be advantageous, or reducing it when this would be helpful.[14,15] It is not even certain that the immune checkpoint blocking antibodies used for cancer immunotherapy[16] are optimally configured. Anti-PD-1 antibodies such as nivolumab (Opdivo) and pembrolizumab (Keytruda) suppress signaling by blocking ligand binding,[17] but pre-clinical data suggest that the Fc regions of non-depleting, inhibitory Fc receptor (FcR)-binding PD-1-blocking antibodies constrain their therapeutic efficacy, perhaps through counter-productive signaling.[18,19] Anti-PD-1 antibodies can trigger inhibitory signaling, with membrane-proximal binders having the largest effects, but why this is so is uncertain.[20]

Here, we tested whether antibody-induced immune receptor signaling can be explained by extending the kinetic-segregation model of immune receptor triggering.[21] In the manner proposed for their native ligands, we previously suggested[22] that the binding of surface-anchored agonistic antibodies to immune receptors creates steric constraints that locally reduce the access of RPTPs to the receptors, favoring net increases in their phosphorylation. Supporting this proposal, our analysis of CD28-binding antibodies indicated that agonists need to be anchored to apposing surfaces and that signaling depends on the sizes of both the RPTPs and the complexes formed by the antibodies and receptors. Moreover, strong agonists were better at excluding RPTPs than weaker-signaling antibodies. These findings allowed us to design a potent anti-PD-1 agonist that suppressed immune reactions in experimental models. Our work suggests that a fuller understanding of their signaling effects would help to improve and extend the therapeutic utility of antibodies.

RESULTS

Properties of differentially signaling anti-CD28 antibodies

Our analysis of antibody agonism was based on comparisons of the mouse (m) anti-rat CD28 antibodies, JJ316 and JJ319.[9] JJ316 is a strong CD28 agonist, inducing primary $CD4^+$ and $CD8^+$ T cell proliferation *in vitro*, and $CD4^+$ T cell lymphocytosis and splenomegaly *in vivo*, in the absence of T cell receptor (TCR) engagement. By contrast, JJ319 is inactive *in vivo* and requires co-ligation of the TCR to initiate signaling *in vitro*. JJ316-induced receptor triggering results in phosphorylation of the exchange factor Vav and adaptor SLP-76, and activation of the phospholipase C and protein kinase Cθ-nuclear factor κB (NF-κB) pathways.[23] Although this does not require TCR engagement, constitutive proximal TCR signaling is needed,[24] as in the case of an anti-human (h) CD28 antibody agonist.[25] The epitopes of JJ316 and JJ319 map to the C″D loop on the "side" of the CD28 homodimer and to a region adjacent to the ligand-binding site at the "top" of the receptor, respectively (Figure S1A).[26] The anti-hCD28 antibodies 5.11A1, the murine precursor of TGN1412, and 7.3B6, a partial agonist, bind in analogous positions, forming complexes differing in length by ~6 nm along an axis orthogonal to the membrane (Figure S1B[22,26]). These length differences were proposed to lead to the differential exclusion of RPTPs by strong and partial or weak agonists (Figure 1A).[22] JJ316 and JJ319 Fabs bound with similar affinity (K_D ~95 nM) and kinetics to CD28 expressed as an immunoglobulin G1 (IgG1) Fc fusion protein (Figure S1C). However, JJ316 bound with a 10-fold larger apparent K_D to cell-expressed CD28 than JJ319 (100 vs. 10 nM), due to a smaller on-rate (Figure S1D). This likely reflects differences in the accessibility of the two epitopes on the surface-expressed receptor.

We generated murine lymphocytic cell lines expressing a chimeric form of CD28 consisting of the ECD of rat CD28 and the transmembrane and cytosolic regions of mCD28. In assays of antibody-induced signaling,[9,26] performed here on glass to facilitate imaging, cells were incubated for 15′ with JJ316 or JJ319 antibodies and then allowed to settle onto surfaces pre-coated with secondary antibodies (e.g., donkey anti-mIgG [DAM]). Levels of bound JJ316 and JJ319 were similar following the 15′ antibody incubations (Figure S1E). Using interleukin-2 (IL-2) production as a readout of signaling,[9,26] JJ319 reduced the EC50 of mitogenic KT3 (anti-CD3ε) antibody-induced signaling by the DO11.10 T cell hybridoma expressing the chimeric form of CD28, consistent with it being a partial agonist (Figure 1B; Table S1). By contrast, JJ316 was active in the absence of the KT3 antibody (Figures 1B and 1C). A second T cell hybridoma, Yae5b3k, and a TCR-expressing derivative of the BW5147 (BW) thymoma yielded similar data (Figures 1D and 1E). JJ316-induced IL-2 secretion by BW cells was sensitive to the levels of CD28 expression and wholly TCR dependent (Figure 1F). Notably, JJ316 also triggered IL-2 production by BW cells expressing a truncated, signaling-disabled form of CD28 (tCD28) when the TCR was expressed at high levels (Figure S1F). This is consistent with other work showing that if the RPTP CD45 is excluded over large enough regions of cellular contact, the TCR can be triggered without TCR ligands being present[28] (see Figure S1G for a fuller explanation for TCR signaling in the absence of ligands). To avoid this, we used BW cells expressing intermediate levels of the TCR and physiological levels of CD28 for our signaling experiments (see Figure 1F legend). Agonist-induced IL-2 production by these cells was not reliant on strong calcium signaling, indicating that CD28 agonists only weakly activate the TCR-proximal signaling pathways on which they depend (Figure 1G), as noted previously.[24,25] Nevertheless, DAM antibody adsorption used as a marker of surface contact and CD69 expression as a measure of activation revealed that most of the JJ316- and JJ319-treated cells contacted the surface (Figures 1H and S1H) and, in the presence of JJ316, upregulated CD69 (Figures 1I, 1J, and S1I), indicating that JJ316 initiates downstream signaling efficiently.

Steric requirements of antibody-induced signaling

Previously, a chimeric form of Lck linked to the ECD of CD45 (CD45RABCLck; Figure 2A, left panel), which is excluded from cellular contacts, was used to show that antibody-induced TCR signaling depends on the kinases present in regions of contact from which large proteins are excluded.[28] Whereas IL-2 production by JJ316-treated TCR^+-$CD28^+$ BW cells was enhanced by over-expressing a compact form of Lck, over-expression of

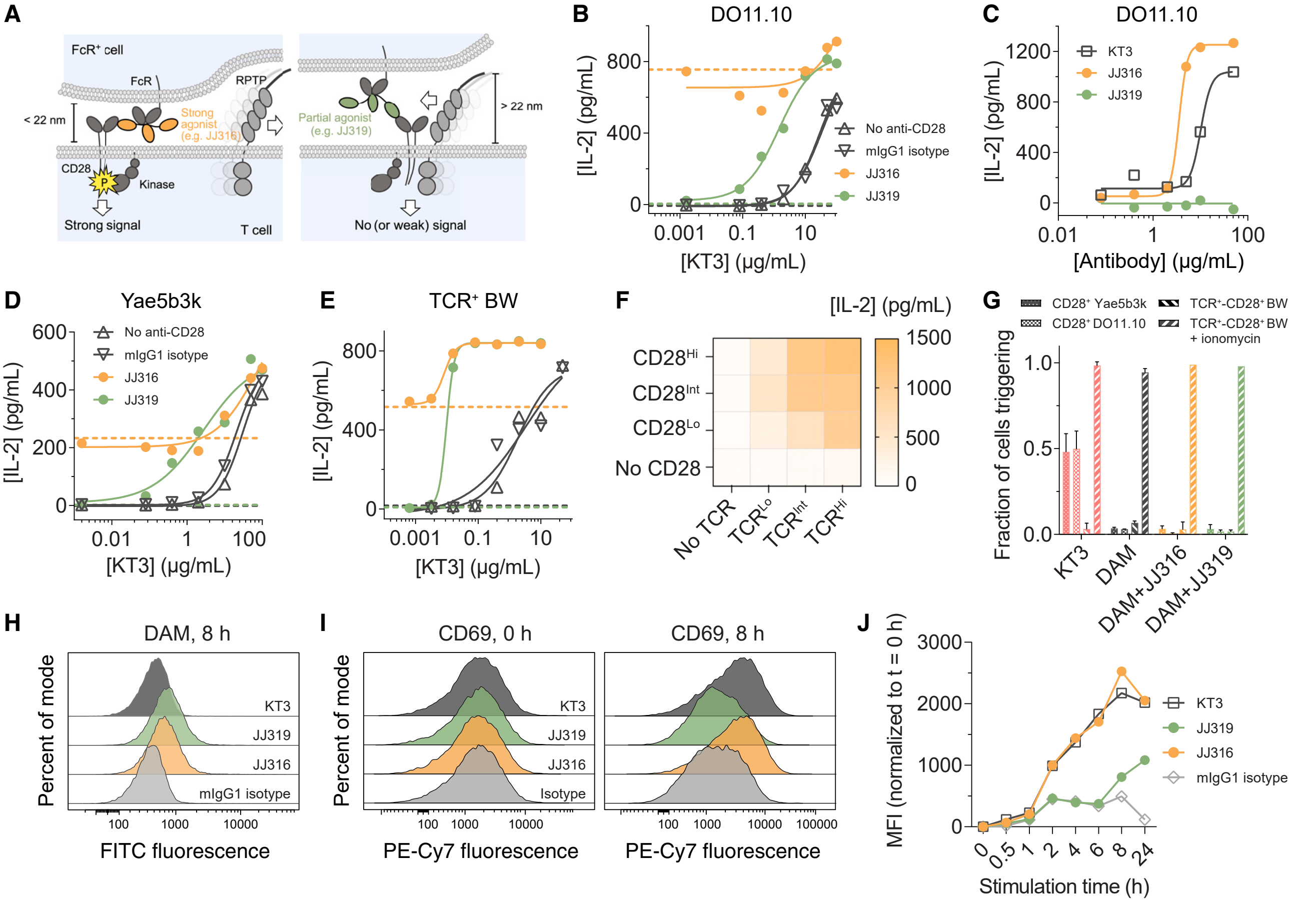

Figure 1. Signaling by agonistic antibodies

(A) The kinetic-segregation model-based explanation for antibody signaling. By binding receptors (e.g., CD28) close to the membrane, FcR-engaged strong antibody agonists (e.g., JJ316; left) create small (<22 nm) gaps between apposing cells that locally exclude large RPTPs, which would otherwise oppose receptor phosphorylation by kinases. By contrast, partial agonists (e.g., JJ319; right) that bind to the "tops" of the receptors generate larger (>22 nm) gaps that less efficiently exclude the RPTPs, producing much weaker signaling.

(B) IL-2 production induced by indirectly immobilized JJ316 (orange) and JJ319 (green) anti-CD28 antibodies (10 μg/mL), either in the absence (dashed lines) or the presence of increasing amounts of immobilized mitogenic KT3 anti-CD3ε antibody (solid lines), in DO11.10 murine T-hybridoma cells expressing a rat-mouse chimeric form of CD28. The black dashed line indicates the amount of IL-2 produced in the absence of antibody.

(C) Titration of the JJ316, JJ319, and KT3 antibodies.

(D and E) IL-2 production by Yae5b3k (D) and TCR-expressing (TCR⁺) BW (E) cells induced by indirectly immobilized JJ316 and JJ319 anti-CD28 antibodies (see B).

(F) Effects of varying the expression of the TCR (~225 [Lo, low], 500 [Int, intermediate], and 1,160 [Hi, high] receptors/cell) and full-length CD28 (~2,380 [Lo], ~11,030 [Int], and ~38,660 [Hi] receptors/cell), on signaling by BW cells induced by JJ316 (10 μg/mL). BW cells expressing intermediate TCR and CD28 levels were used for experiments in Figures 1G–1J, 2, S1H, and S1I. Primary T cells are estimated to express ~9,000 copies of CD28.[27]

(G) Calcium signaling responses of CD28-transduced DO11.10, Yae5b3k, and TCR⁺ BW cell lines loaded with Fluo-4 and stimulated with KT3 directly coated onto glass coverslips, or with JJ316 or JJ319 antibody indirectly immobilized via DAM; 1 μM ionomycin was used to confirm the signaling capacity of the TCR⁺ BW cells. Error bars are standard deviations (SDs).

(H) Staining of cells for DAM adsorption from DAM antibody-coated surfaces following culture for 8 h under the conditions described in (B), in the presence of the indicated antibodies at 10 μg/mL, using a FITC-labeled rabbit anti-donkey IgG antibody.

(I) Staining of cells for CD69 expression prior to (0 h) and following an 8 h culture as in (H), using a PE-Cy7-labeled anti-mCD69 antibody.

(J) Time course of CD69 expression, measured as in (I), during a 24 h culture as in (H); MFI, mean fluorescence intensity.

Signaling data were fitted to a binding model as indicated in Table S1. Values for IL-2 produced in the absence of antibodies, indicated by broken lines in (B), (D), and (E), were included in the fitting and analysis. The data are representative of 2–4 independent replicate experiments.

the CD45-Lck construct did not produce a similar increase in signaling (Figure 2A, right panel). Like antibody-triggered TCR signaling, therefore, JJ316-induced signaling by CD28 relies on kinases present in cellular contacts that exclude large proteins including CD45.

We addressed whether RPTP exclusion from such contacts suffices to explain antibody signaling. First, we determined whether activating antibodies must be anchored to surfaces, i.e., to create steric barriers to RPTP diffusion. We found that immobilized, but not soluble, bivalent JJ316 Fab′$_2$ induced IL-2 production by

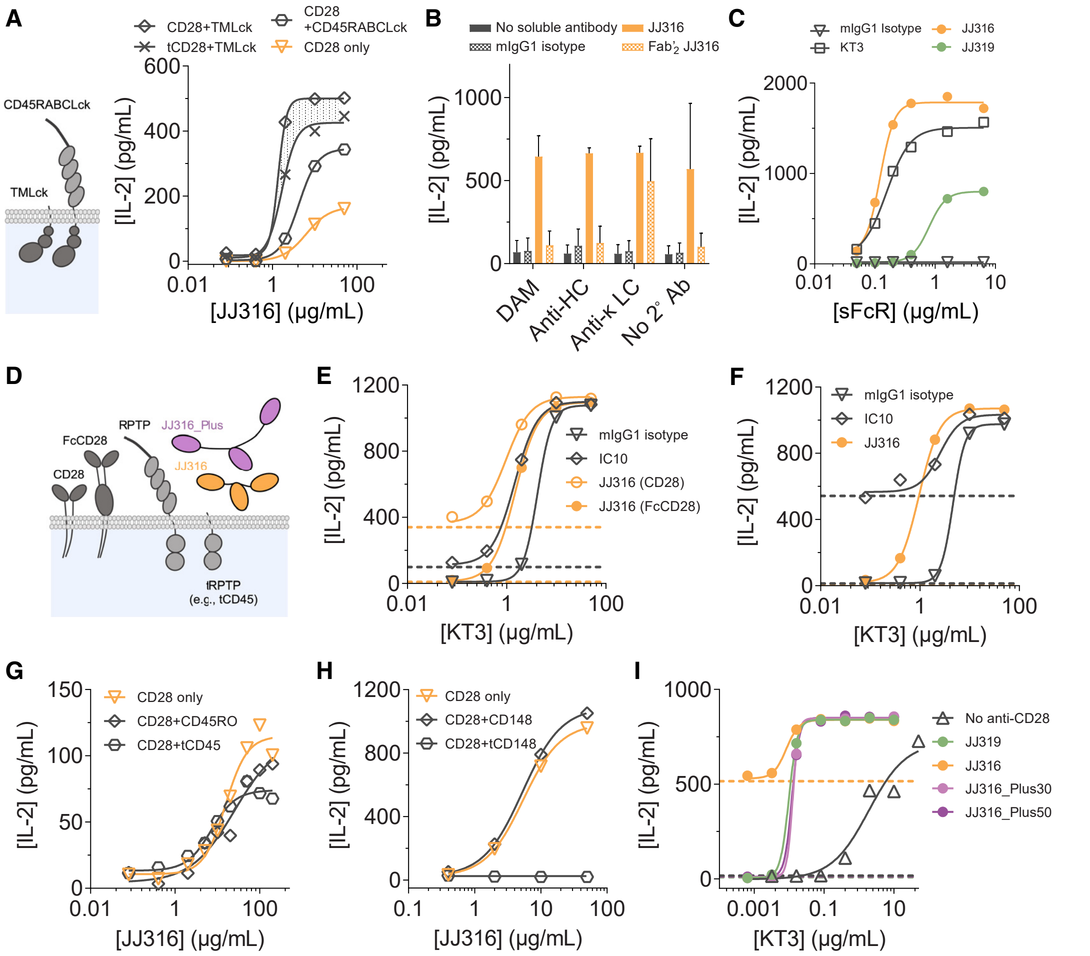

Figure 2. Dependence of signaling by agonistic antibodies on their immobilization and on kinase, receptor, RPTP, and antibody dimensions

(A) IL-2 production by BW cells expressing intermediate levels of CD28 (or tCD28) and the TCR (see Figure 1F), treated with JJ316 antibody following expression of compact or extended forms of hemagglutinin (HA)-tagged Lck (left); Lck expression levels were comparable at MFI values of 4,071 and 3,471 measured with a PE-conjugated anti-HA antibody, respectively. The stippling (right) marks the contribution of CD28 to the increased signaling in the presence of TMLck. Figure S1G explains why there is signaling in the presence of tCD28.

(B) Signaling effects of immobilized and soluble forms of intact or Fab′$_2$ fragments of the JJ316 antibody (at 10 μg/mL), measured with TCR$^+$ BW cells expressing intermediate levels of CD28 (HC, heavy chain; LC, light chain; 2°, secondary). Error bars represent SD.

(C) Effects of JJ316, JJ319, and KT3 antibodies coupled indirectly at high levels to Ni-NTA-coated plastic via histidine-tagged sFcR, on signaling by CD28-expressing TCR$^+$ BW cells.

(D) Schematic showing how the dimensions of CD28, the RPTPs CD45 and CD148, and the JJ316 antibody were altered.

(E and F) Signaling effects of JJ316 and IC10 (anti-hIgG1 Fc) antibodies on TCR$^+$ BW cells expressing intermediate (E) and high (F) levels of a form of CD28 (FcCD28) extended via the insertion of the Fc region of hIgG1 at the junction between the extracellular and transmembrane regions of the receptor. The intermediate and high levels of expression of FcCD28 were comparable to those for CD28 in Figure 1F.

(G and H) Effect of co-expressing truncated forms of the RPTPs CD45 (G) and CD148 (H) on signaling by CD28-expressing TCR$^+$ BW cells.

(I) Effects on signaling of extending the hinge region of JJ316 with 30 or 50 residues of mucin-like sequence.

Signaling data were fitted to a binding model as indicated in Table S1. Values for IL-2 produced in the absence of antibodies, indicated by broken lines in (E), (F), and (I), were included in the fitting and analysis. The data are representative of 2–4 independent replicate experiments.

TCR$^+$-CD28$^+$ BW cells (Figure 2B), confirming the anchorage-dependence of antibody-induced signaling and that receptor cross-linking is insufficient to trigger signaling. Notably, whole JJ316 antibody was also active in solution, which is explained by the cells presenting JJ316 to one another in an Fc-dependent manner. Importantly, JJ319 induced IL-2 production by TCR$^+$-CD28$^+$ BW cells in the absence of TCR ligation (Figure 2C) when it was presented at very high levels on plastic surfaces via a soluble form of FcγR2b (sFcR; Figure S2A), emphasizing the quantitative nature of the signaling effects of the antibodies.

To confirm that surface attachment creates steric constraints required for signaling, we examined the effects of varying the dimensions of (1) the receptor, (2) the RPTPs CD45 and CD148, and (3) the agonistic antibody itself (Figure 2D). First, the length of the receptor was increased by ∼75 Å by inserting the Fc region of hIgG1 at the junction between the extracellular and

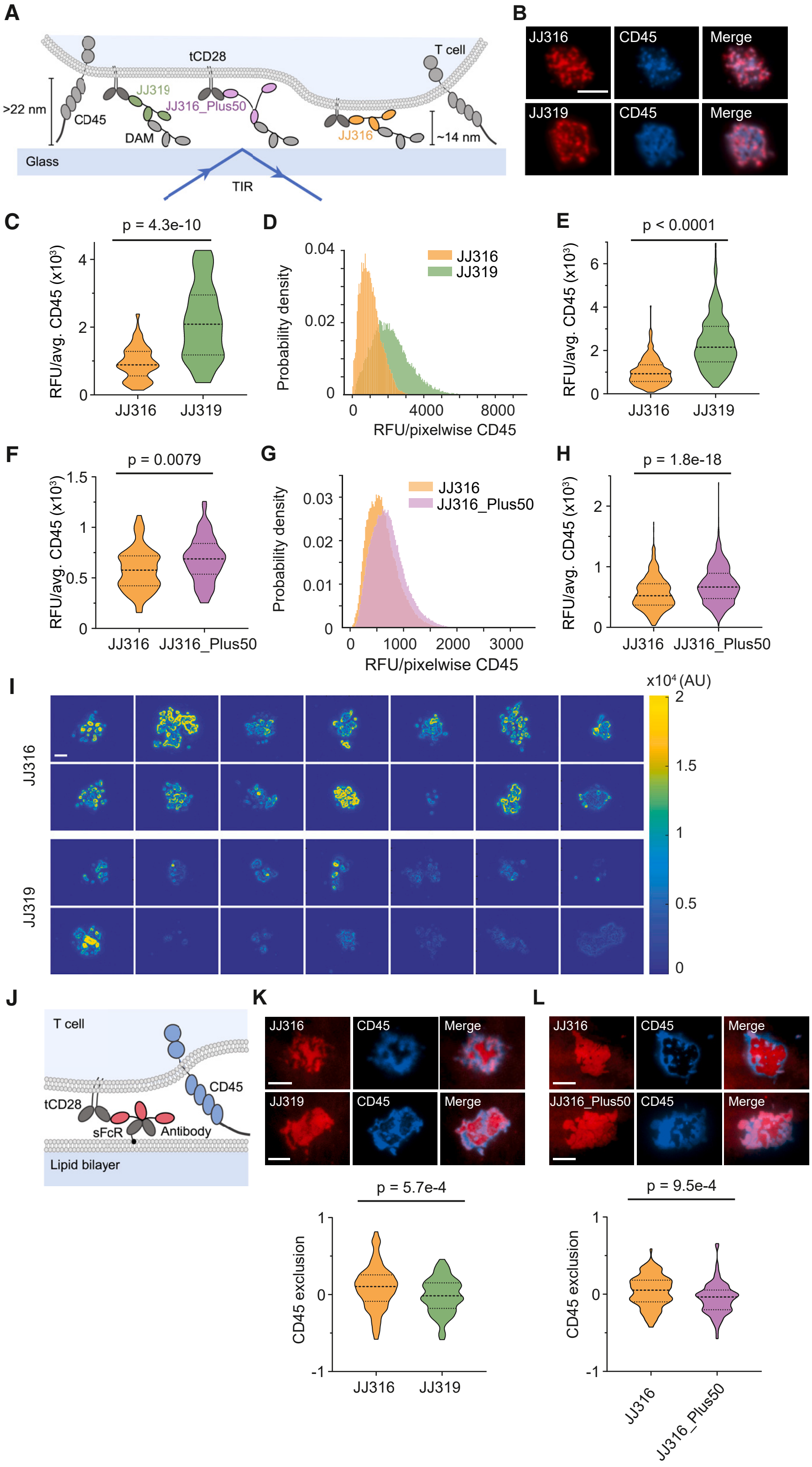

Figure 3. Anti-CD28 antibodies locally exclude CD45 from sites of contact

(A) Schematic showing the setting for analyzing the effects of anti-CD28 antibodies on CD45 distribution at contacts of T cells with DAM-coated glass surfaces, using TIRF imaging.

(legend continued on next page)

transmembrane regions of CD28 (creating “FcCD28”). JJ316 binding to FcCD28 was faster than to CD28 (Figure S2B), but JJ316 was then costimulatory rather than agonistic for TCR+ BW cells expressing the extended receptor (Figure 2E). An anti-hIgG1 Fc antibody (IC10) that bound the CH2 domain of FcCD28, i.e., closer to the membrane than JJ316, was weakly (Figure 2E) or highly (Figure 2F) agonistic, depending on the level of FcCD28 expression. Second, co-expression of mCD45 lacking its ECD (i.e., “tCD45”) in TCR+-CD28+ BW cells suppressed JJ316-induced IL-2 production by 30%–40% (Figure 2G). Truncated mCD148 (i.e., “tCD148”) was an even more potent suppressor of signaling (Figure 2H). Third, we inserted 30 or 50 residues of mucin-like mCD43 sequence into the hinge region of JJ316 (creating “JJ316_Plus” antibodies). This increased the Stoke’s radius, i.e., length of JJ316 (Figure S2B), without affecting its binding capacity for cell-expressed CD28 (Figure S2C), its ability to bind to purified CD28 (Figure S2D), or its affinity for sFcR (Figure S2E), indicating that the structure of the antibody was otherwise unaffected. Instead of being agonistic, both JJ316_Plus30 and JJ316_Plus50 were costimulatory (Figure 2I). These data imply that the intensity of signaling induced by anti-CD28 antibody-receptor complexes is governed by steric effects that determine their accessibility by RPTPs.

Antibody agonists locally exclude RPTPs from bound receptors

To establish a direct link between signaling complex architecture and RPTP distribution, we tested whether antibody agonists are better at locally excluding RPTPs than weaker-signaling antibodies, using total internal reflection fluorescence (TIRF) imaging. To avoid the confounding effects of signaling-induced changes in protein organization,[29] we analyzed TCR-deficient BW cells expressing signaling-disabled tCD28 (Figure 3A). CD45, but not CD148, was studied because CD148 is weakly expressed on resting T cells[30] and unlikely to contribute to early CD28 signaling.

TCR-deficient tCD28+ BW cells were imaged on DAM-coated coverslips following 15′ incubations with the antibodies, mimicking the conditions of the IL-2 signaling assays. In TIRF images of fluorescent antibody and CellMask-labeled cells, antibody fluorescence was strongly correlated with membrane staining (Figures S3A and S3D). This allowed us to use antibody fluorescence intensity to identify regions of cell contact. Within a few minutes of contact with the surface, cells labeled with fluorescent JJ316 or JJ319 antibodies and anti-CD45 Fab fragments had spread, and antibody and CD45 staining across the contact had become non-uniform, although there was considerable overlap in the two signals (Figure 3B; line scans are shown in Figure S4A; Video S1). We used image masking to measure differences in CD45 fluorescence in the regions of contact marked by antibody fluorescence. An “antibody” mask was created by intensity-based thresholding (>40% of max. intensity), allowing average and per-pixel CD45 intensities to be measured across the entire region of antibody-mediated contact (Figure S3B). An antibody “high” sub mask (>60% of max. intensity) was used to measure CD45 fluorescence in regions of close contact, marked by increased antibody fluorescence (Figure S3C). CD45 intensities across the entire contact, both on average (Figure 3C) and on a per-pixel basis (Figure 3D), and in regions of close contact (Figure 3E), were lower for JJ316- than for JJ319-treated cells. Similar results were obtained for images analyzed using an alternative (i.e., Otsu) method to create antibody masks (Figure S4D). The distribution of contact sizes was similar for both antibodies, and there was no correlation between contact size and CD45 fluorescence (Figure S4E). By all measures, CD45 fluorescence was also higher in the presence of the extended form of JJ316, i.e., JJ316_Plus50 (Figures 3F–3H), which produced weaker signaling than JJ316 (Figure 2I). At a lower level of tCD28 expression comparable to that of CD28 in BW cells used in the IL-2 release assays (TCR^{Int}-CD28^{Int} cells, Figure 1F), the average CD45 intensity per cell was similar for the two antibodies, but the CD45 fluorescence measured on a per-pixel basis and in regions of close contact was lower for JJ316- versus JJ319-treated cells (Figure S4F). These data indicate that JJ316 is better at excluding CD45 from regions of antibody-receptor complex formation than JJ319, an effect more easily observed at higher tCD28 expression.

In a bespoke, orthogonal approach we call “gradient vector distance (GVD)” analysis, we used the fluorescence data to draw inferences about the behavior of CD45 at length scales below the diffraction limit. For each pixel in a fluorescence image, local gradients in CD45 and antibody fluorescence were measured using the intensities in the surrounding eight pixels (Figure S5A). The gradients were then used to create scalar vectors in each channel for each pixel, which were then subtracted. For large differences, which indicated that the antibody and CD45 fluorescence signals were strongly anti-correlated, i.e., that the antibodies and CD45 were exhibiting a tendency to

(B) TIRF images of tCD28-expressing BW cells labeled with Alexa Fluor 647-tagged JJ316 or JJ319 antibody (red) and Alexa Fluor 488-tagged YW62.3.20 anti-CD45 Fab fragments (blue), interacting with DAM-coated coverslips. Note that antibody fluorescence was strongly correlated with membrane (CellMask) staining (Figure S3A), so dark regions in the antibody channel correspond to parts of the cell outside the evanescent field.

(C–E) Average CD45 fluorescence intensities for each cell (C), histograms showing the probability density for the pixelwise CD45 fluorescence intensity for all cells (D), and average CD45 intensities in regions of high antibody fluorescence (E; N = 46 cells [JJ316], N = 49 cells [JJ319]). In the violin plots, dashed lines indicate the median, and dotted lines the quartiles.

(F–H) As in (C)–(E), comparing the effects of JJ316 antibody (N = 65 cells) and JJ316 with a 50-residue extension (JJ316_Plus50; N = 69 cells).

(I) GVD analysis of CD45 versus antibody distribution. The degree to which the antibodies and CD45 tend to localize in different regions is indicated by the color scale (yellow, high; blue, low). AU, arbitrary units. See Figure S5 for a description and for simulation-based validation of the GVD method.

(J) Schematic showing the setting for analyzing the effects of anti-CD28 antibodies on CD45 distribution at contacts of T cells with sFcR-antibody-presenting SLBs.

(K and L) Mask-based analysis of CD45 exclusion from regions of antibody accumulation for (K) cells that interacted with JJ316 (N = 142 cells) versus JJ319 antibody (N = 133 cells), and (L) cells that interacted with JJ316 (N = 114 cells) versus JJ316_Plus50 antibody (N = 95 cells), on the sFcR-antibody-presenting SLBs. Scale bars are 5 μm. Data were combined from experiments performed over 3 separate days. A two-sample Student’s t test was used for statistical comparisons. For these experiments, tCD28 expression matched that of TCR^{Int}-CD28^{Hi} BW cells (see Figure 1F).

accumulate in different regions, the pixels were re-colored yellow, and for small differences indicating that the antibodies and CD45 were behaving similarly, the pixels were re-colored blue (Figure S5A). The analysis indicated that, across a population of cells, JJ316 produced more strongly anti-correlated (yellow) signals than JJ319 (Figure 3I), indicating that the antibodies were acting locally to effect differences in CD45 exclusion, likely on length scales below the diffraction limit. More analysis of the data revealed that only the CD45 fluorescence intensity differed: there were no differences in JJ316 and JJ319 antibody accumulation or in the degree of antibody-induced anti-correlation (Figure S5B). Simulations supported this interpretation of the data (see Figure S5C).

CD45 redistribution was also examined in the more physiological setting of supported lipid bilayers (SLBs). We prepared SLBs presenting sFcR, which were then loaded with JJ316 or JJ319 antibodies (Figure 3J). When TCR-deficient tCD28$^+$ BW cells formed contacts with the SLBs, the sFcR-antibody ligands organized into very clear regions of antibody-bound CD28 accumulation and CD45 exclusion (Figure 3K; Video S2, line scans; Figures S4B and S4C). Re-organization on this scale was likely not possible on glass surfaces because antibody diffusion was constrained by DAM immobilization. For the SLB images, however, a "CD45" mask could be used to directly measure CD45 fluorescence inside and outside contacts identified by antibody fluorescence, allowing calculation of CD45 "exclusion" (Figure S3E):

$$\text{Exclusion} = 1 - (\text{Avg CD45}_{\text{in}}) / (\text{Avg CD45}_{\text{out}}).$$

According to this metric, in this more physiological setting, JJ316 was also significantly better at excluding CD45 than JJ319 (Figure 3K) and JJ316_Plus (Figure 3L). The diffusional behavior of CD28, which was measurable in the more distinct contacts formed on SLBs, was comparable in the presence of JJ316 and JJ319 (Figure S5D), indicating that signaling is not explained in this instance by slowed diffusion of the receptor in the presence of the antibodies.[31]

Agonistic signaling by anti-PD-1 antibodies

The dependence of agonistic signaling on local RPTP exclusion suggested that all immune receptors might, in principle, be agonizable. To confirm this, we produced an anti-PD-1 agonist that triggers signaling by binding PD-1 membrane proximally. A mIgG1 anti-PD-1 antibody, clone 19, bound with high affinity (Figure S6A) to the base of the folded region of the receptor (Figure 4A) and did not block PD-L1 or PD-L2 binding (Figure S6B). A second IgG1 antibody, clone 2, bound with comparable affinity (Figure S6A) closer to the top of PD-1 (Figure 4A) and blocked both ligands (Figure S6B). Noting that anti-CD28 antibodies are all either strong or partial agonists,[26] we anticipated that antibodies binding to small receptors could be a special case insofar as they would all exclude RPTPs to some extent and have a degree of signaling activity, including anti-PD-1 antibodies used clinically to block PD-1 signaling.[6,16] To address this in our assays, we generated a chimeric antibody consisting of the variable domains of the PD-1-blocking antibody nivolumab[32] fused to the constant regions of mIgG1 (Nivo_mIgG1).

Attached to SLBs via sFcR, clone 19, clone 2, and Nivo_mIgG1 excluded CD45 from bilayer contacts formed by truncated PD-1-expressing TCR-deficient BW cells (Figures 4B and 4C). Clone 19 had a larger effect than clone 2, further confirming that the level of RPTP exclusion is determined by epitope position. Clone 19 also caused a more significant reduction in interferon γ (IFNγ) production than clone 2 in an *in vitro* activation assay with human peripheral blood mononuclear cells (PBMCs; Figure 4D), indicating that it is agonistic and that its greater ability to exclude CD45 correlates with its more effective initiation of PD-1 signaling. Although Nivo_mIgG1 did not reduce IFNγ production by human PBMCs (Figure 4D), the antibody strongly suppressed T cell activation in a reporter assay (Figure 4E). In this assay,[33] PD-1 expressing Jurkat T cells that produce luciferase under the control of a nuclear factor of activated T cells (NFAT) response element are cultured with "T cell stimulator" (TCS) cells expressing both a TCR-engaging anti-CD3 (OKT3) construct and full-length mFcγR2b. In this setting, in contrast to the unmutated antibody, a form of Nivo_mIgG1 mutated at D265 to block FcR binding (Nivo_D265A),[34] was not agonistic (Figure 4E). Similarly, Nivo_mIgG1 extended in the hinge region with 50 residues of mucin-like mCD43 sequence (Nivo_Plus), which excluded CD45 very inefficiently on bilayers (Figure 4C), was also less agonistic (Figure 4E). When the TCS cells expressed PD-L1 rather than mFcγR2b, all three forms of nivolumab and clone 2 enhanced T cell activation equally (Figure 4F). For TCS cells expressing both PD-L1 and mFcγR2b, only Nivo_D265A strongly enhanced T cell activation (Figure 4G). In the absence of PD-L1 and FcRs, none of the antibodies had blocking or agonistic effects (Figure 4H). Importantly, in the presence of hFcγR2b, nivolumab and pembrolizumab hIgG4 biosimilars exhibited strong agonistic activity, as did a humanized IgG4 version of clone 19 (Figure 4I). These experiments showed (1) that epitope position affects the extent of PD-1 signaling by anti-PD-1 antibodies, (2) that the antibodies must be immobilized (e.g., on FcRs), (3) that signaling is sensitive to the dimensions of the complex formed by the anti-PD-1 antibody and the receptor, and (4) that stronger-signaling anti-PD-1 antibodies are better at excluding CD45 than weaker-signaling antibodies. Anti-PD-1 antibody agonists therefore function in the manner of mitogenic anti-CD28 antibodies.

Receptor triggering by anti-PD-1 antibodies

To confirm that anti-PD-1 antibody agonists initiate signaling by triggering PD-1 phosphorylation, we used the recruitment of the cytosolic Src homology 2 (SH2) domain-containing phosphatase (SHP)2 to the phosphorylated receptor via its tandem SH2 domains[35–37] as a reporter. A fluorescent form of SHP2 was expressed along with PD-1 in Jurkat T cells lacking TCRs to avoid, once again, the confounding effects of signaling-induced changes in protein re-organization. The cells were then placed onto sFcR-functionalized SLBs presenting anti-PD-1 antibody (Figure 5A). Three-color TIRF imaging of the PD-1$^+$-TCR$^-$ cells revealed strong SHP2 recruitment, detected as "negative exclusion" in our analysis, in regions of local antibody accumulation and CD45 exclusion, in a manner requiring the presence of the cytosolic tail of the receptor (Figures 5B and 5C; Video S3). Clone 2 and Nivo_mIgG1 each produced weaker SHP2 recruitment than clone 19; however, Nivo_Plus produced the weakest accumulation (Figures 5B and 5C). At higher levels of antibody immobilization, clone 2 induced more SHP2 accumulation, further

CellPress
OPEN ACCESS

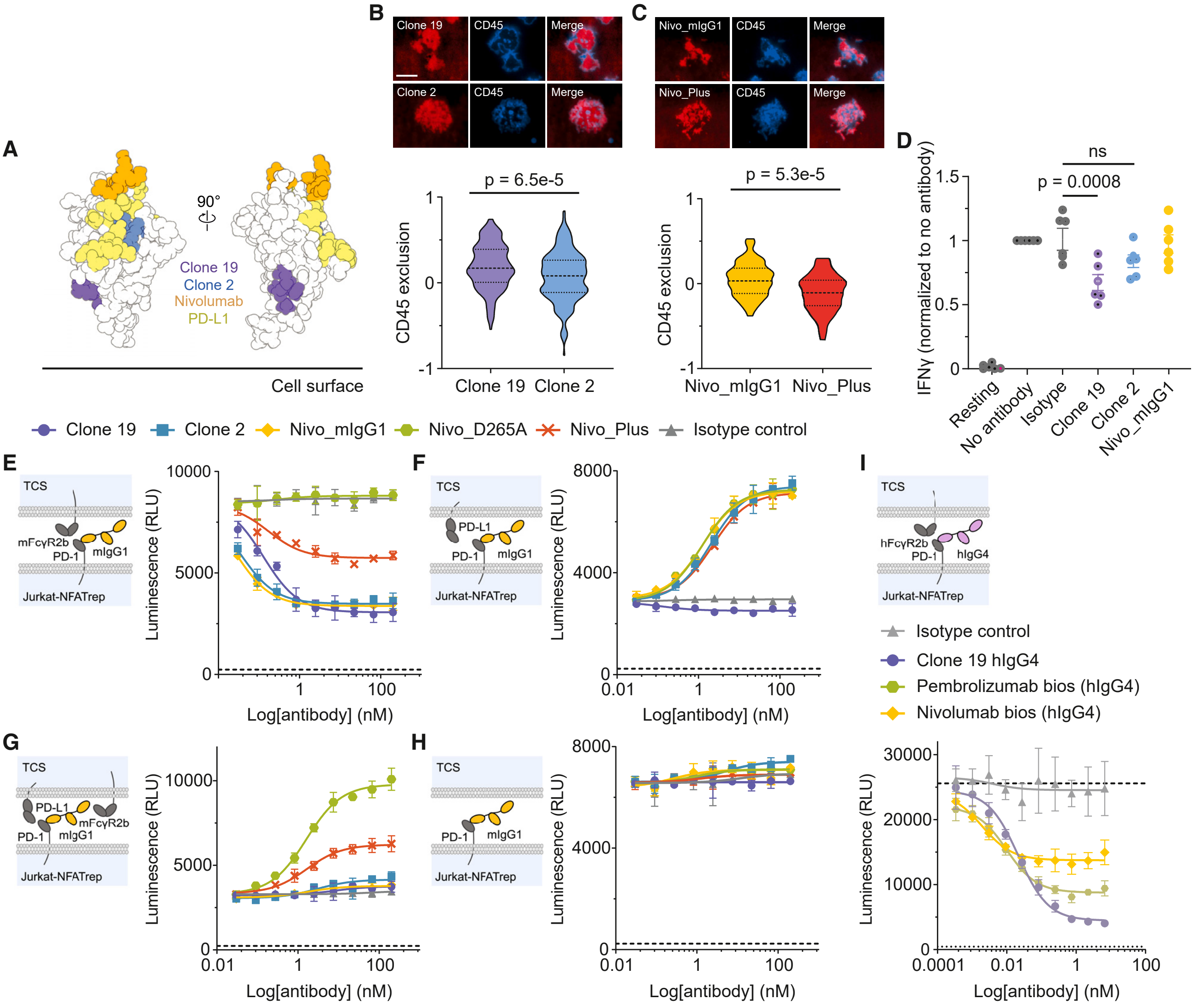

Figure 4. Effects of anti-PD-1 antibodies on CD45 exclusion and signaling *in vitro*

(A) Positions of the epitopes of the clone 19 (purple), clone 2 (blue), and nivolumab (orange) anti-PD-1 antibodies and of the binding site for PD-L1 (yellow) on PD-1, relative to the membrane.

(B and C) Mask-based analysis of CD45 exclusion from regions of high antibody fluorescence intensity for (B) cells treated with clone 19 (N = 204 cells) and clone 2 (N = 212 cells), and (C) cells treated with nivolumab expressed as a mIgG1 antibody (Nivo_mIgG1; N = 58 cells) or as an extended mIgG1 (Nivo_Plus; N = 61 cells). The setting for the imaging experiments was analogous to that in Figure 3J. In the violin plots, dashed lines indicate the median, and dotted lines the quartiles. Scale bar is 5 μm. Data were combined from experiments performed over 3 separate days. A two-sample Student's t test was used for statistical comparisons.

(D) Impact of anti-PD-1 antibodies on IFNγ production by human PBMCs activated with anti-CD3 and anti-CD28 antibodies. Each symbol represents a different healthy donor, with IFNγ levels normalized to the "no antibody" condition for each donor. One-way ANOVA with Dunnett's multiple comparison follow-up testing was used to compare each group to the mIgG1 isotype control.

(E–H) Effects of clone 19, clone 2, Nivo_mIgG1, Nivo_D265A, and Nivo_Plus on T cell activation in a co-culture NFAT reporter system. Jurkat T cells expressing hPD-1 and a luciferase reporter driven by an NFAT response element (NFATrep) were cultured with TCS cells expressing an anti-CD3 (OKT3) scFv antibody construct. Signaling effects of PD-1 antibodies were measured in four settings: Jurkat PD-1 cells cultured with (E) TCS cells expressing mFcγR2b, (F) TCS cells expressing PD-L1, (G) TCS cells expressing PD-L1 and mFcγR2b, and (H) unmodified TCS cells. The dashed horizontal line in each plot indicates the level of luciferase production by resting Jurkat T cells.

(I) Effects of hIgG4 isotype PD-1 antibodies, i.e., humanized clone 19 and nivolumab and pembrolizumab biosimilars (bios), on T cell activation in the reporter system with PD-1-expressing Jurkat T cells cultured with TCS cells expressing hFcγR2b.

Error bars shown represent SD.

confirming the quantitative nature of antibody-induced signaling (Figure S6C; see also Figure 2C). The extent of local phosphatase exclusion and SHP2 recruitment matched that produced by a soluble form of the PD-1 ligand, PD-L1 (sPD-L1), immobilized on the SLB (Figures 5B, 5D, and 5E). The effects were tyrosine phosphorylation-dependent because SHP2 recruitment

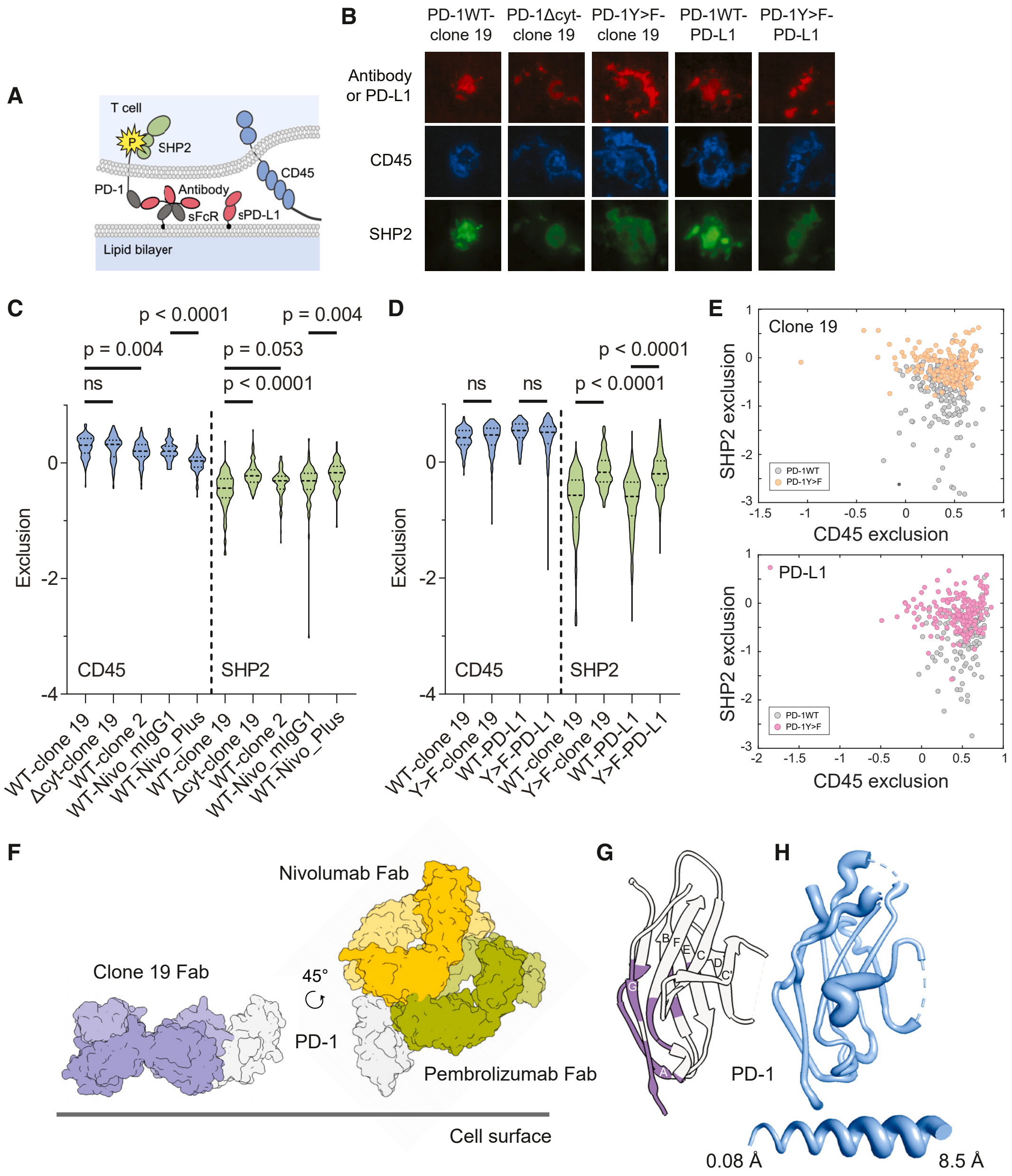

Figure 5. Signaling effects of PD-1 agonists

(A) Schematic showing the setting for analyzing the effects of anti-PD-1 antibodies on CD45 and SHP2 distribution at contacts of T cells with sFcR-antibody- or sPD-L1-presenting SLBs, using TIRF imaging.

(B) TIRF images of TCR^{-} Jurkat T cells expressing a fluorescent form of SHP2 (SHP2Halo) and wild-type PD-1 (PD-1WT), PD-1 lacking its cytosolic domain (PD-1Δcyt), or PD-1 with its cytosolic tyrosine residues mutated to phenylalanine (PD-1Y>F). The cells were incubated with Janelia Fluor HaloTag 646 ligand to label SHP2Halo (green) and Alexa Fluor 555-tagged Gap8.3 anti-CD45 Fab fragments (blue) before interacting with Alexa Fluor 647-tagged clone 19 (presented by sFcR) or sPD-L1 (red) on SLBs.

(C) Violin plots of CD45 (blue) and SHP2 (green) mask-based exclusion values comparing different antibody-mediated contacts: WT-clone 19 (N = 101 cells), PD-1Δcyt-clone 19 (N = 122 cells), WT-clone 2 (N = 110 cells), WT-Nivo_mIgG1 (N = 78 cells), and WT-Nivo_Plus (N = 83 cells), for cells expressing either PD-1WT (WT) or PD-1Δcyt (Δcyt).

(D) Violin plots of CD45 (blue) and SHP2 (green) exclusion values comparing clone 19 and PD-L1 mediated contacts: WT-clone 19 (N = 193 cells), Y>F-clone 19 (N = 173 cells), WT-PD-L1 (N = 136 cells), and Y>F-PD-L1 (N = 168 cells), for cells expressing either PD-1WT (WT) or PD-1Y>F (Y>F). In (C) and (D), the Kruskal-Wallis test with Dunn's multiple comparison follow-up testing was used to compare each group to the WT control.

(E) Dot plots comparing the CD45 and SHP2 exclusion values for cells expressing PD-1WT (gray), PD-1Y>F (orange), or PD-1Y>F (pink), following clone 19 (top) or PD-L1 (bottom) mediated contact.

(F) Structure of the PD-1 (white)-clone 19 Fab (purple) complex (left; PDB: 8eq6) compared with the positioning of nivolumab (orange; PDB: 5WT9) and pembrolizumab (green; PDB: 5JXE) Fab fragments bound to PD-1 (right). The position of PD-1 in the right versus the left panel differs by a 45° anti-clockwise rotation in the plane of the page.

(legend continued on next page)

was significantly reduced after the cytosolic tyrosines were mutated to phenylalanine (Figures 5B, 5D, and 5E). These data indicate that anti-PD-1 antibody agonists initiate inhibitory signaling by triggering PD-1 phosphorylation, at levels comparable to that induced by a native ligand and in inverse proportion to the dimensions of the immobilized antibody-receptor complex.

To shed more light on how clone 19 triggers signaling, we undertook a crystallographic analysis of the agonist-receptor complex (data collection and refinement statistics, Table S2; example electron density, Figure S6D). Confirming the mutational data (Figure 4A), clone 19 bound at the base of PD-1, in contrast to nivolumab and pembrolizumab that bind overlapping sites toward the top (Figure 5F).[38,39] Strikingly, the distance of the epitopes of clone 19, pembrolizumab, and nivolumab from the membrane correlated (inversely) with each antibody's signaling capacity in the reporter assay (Figure 4I). The clone 19 epitope comprises membrane-proximal segments of the A, F, and G β strands (Figure 5G) and is unremarkable versus those of other protein antigen-binding antibodies (detailed comparisons with nivolumab and pembrolizumab are made in Table S3). Importantly, the β barrel comprising the core of PD-1 was unchanged by Fab binding (root-mean-square deviation = 0.74 Å for 53 equivalent β strand residues in the Fab-bound and apo PD-1 structures; Figure 5H), indicating that signaling induced by clone 19 is not accompanied by large-scale structural rearrangements of the receptor.

In vivo effects of the anti-PD-1 antibodies

To test the PD-1 antibodies *in vivo*, we generated "humanized" PD-1 (huPD-1) C57BL/6 mice by replacing the *Pdcd1* exon encoding the ligand-binding domain of mPD-1 with the equivalent human *PDCD1* exon (Figure S7A). These mice expressed the chimeric receptor under the appropriate transcriptional control (Figure S7B) and developed normal immune systems (Figure S7C[40]). The huPD-1 mice were then crossed with an ovalbumin-specific OT-II CD4$^+$ TCR transgenic line (to homozygosity for huPD-1 and heterozygosity for the OT-II transgene), to create a humanized strain with CD4$^+$ T cells of defined TCR specificity. Consistent with the *in vitro* data obtained with the reporter assay, clone 19 and Nivo_mIgG1 both suppressed the antigen-specific expansion of humanized OT-II T cells from these mice following transfer to allelically marked hosts (Figures 6A and S7D). By contrast, both Nivo_Plus and Nivo_D265A boosted T cell expansion (Figure 6A), indicating that they are better inhibitors of PD-1 signaling and confirming the dependence of antibody signaling *in vivo* on antibody-receptor complex dimensions and FcR immobilization, respectively.

Finally, since a non-ligand-blocking PD-1 agonist could have therapeutic utility, we tested the effect of clone 19 in murine models of autoimmunity and inflammation. Clone 19 strongly suppressed keyhole limpet hemocyanin (KLH)-induced delayed-type hypersensitivity (DTH) in huPD-1 mice (Figure 6B) and was also highly suppressive in a cell transfer model of systemic lupus erythematosus (SLE; Figure 6C), a model in which PD-1 expressing T-follicular helper (Tfh) cells are key drivers of disease.[41] Notably, clone 19 did not reduce the number of splenic Tfh cells measured 2 days following antibody injection despite the high levels of expression of PD-1 by these cells (Figure S7E) and their known susceptibility to elimination with depleting antibodies,[42] indicating that the IgG1 antibody is non-depleting. Presumably, the size of the complex[43] and the low activating/inhibitory ratio of mIgG1[44] combined to prevent depletion. These data suggest that the use of a non-depleting, non-ligand-blocking, membrane-proximally binding agonistic PD-1 antibody could be an effective therapy for autoimmune diseases. Owing to the weaker-signaling activities of nivolumab and pembrolizumab, these antibodies were not tested in the disease models.

DISCUSSION

We found that immune receptor signaling triggered by antibodies relies on local RPTP exclusion. Signaling required antibody immobilization and was sensitive to the position of an antibody's epitope relative to the membrane and the sizes of RPTPs, indicating that RPTP exclusion is a steric effect. Engineering small changes in antibody or receptor size altered the degree of agonism or partial agonism, consistent with biophysical studies showing that "height" differences of just 4 nm may effect protein re-organization at cell-cell contacts.[45–47] Strong agonists were better at excluding phosphatases, although the large signaling differences resulted from relatively modest changes in RPTP redistribution: a strong agonist excluded CD45 1.1- to 1.9-fold more effectively than a weaker-signaling antibody on glass, and ~6-fold better on bilayers. The cells interacted similarly with the immobilized antibodies, suggesting that the higher level of CD45 exclusion produced by strong agonists, perhaps over multiple encounters, increases the probability of receptor triggering beyond a threshold needed for secondary signaling events to occur. Signaling in T cells therefore appears to be sensitive to relatively small changes in receptor phosphorylation. Elsewhere, we have shown that 1.7-fold depletion of CD45 suffices to initiate strong TCR signaling.[48]

These data strengthen the case that the kinetic-segregation model[21] accounts for receptor triggering. Nevertheless, other explanations for how anti-CD28 and anti-PD-1 antibodies could trigger signaling must be considered. Antibodies can modulate signaling in an epitope position-dependent manner by blocking structural transitions in large, flexible proteins such as ErbB receptors.[49] However, the crystal structure of the complex of the ECD of PD-1 with clone 19 showing that the structural core of the ECD was unchanged by antibody binding rules this out as a general explanation for antibody-induced signaling. Since the Fab$'_2$ form of the JJ316 antibody was inactive in solution despite being capable of cross-linking CD28 dimers, it also seems unlikely that receptor aggregation per se explains the agonistic effects we observed. Finally, neither of these alternative mechanisms, nor force-dependent signaling[50] account for

(G) Ribbon representation of PD-1 with the clone 19 epitope marked in purple.
(H) Structural differences between apo PD-1 (PDB: 3RRQ) and PD-1 in the clone 19 Fab-PD-1 complex, mapped onto PD-1 from the Fab-PD-1 complex. Thickness of the putty cartoon representation corresponds to the distance between equivalent Cα atoms after superposition. Distances vary between 0.08 and 8.5 Å.

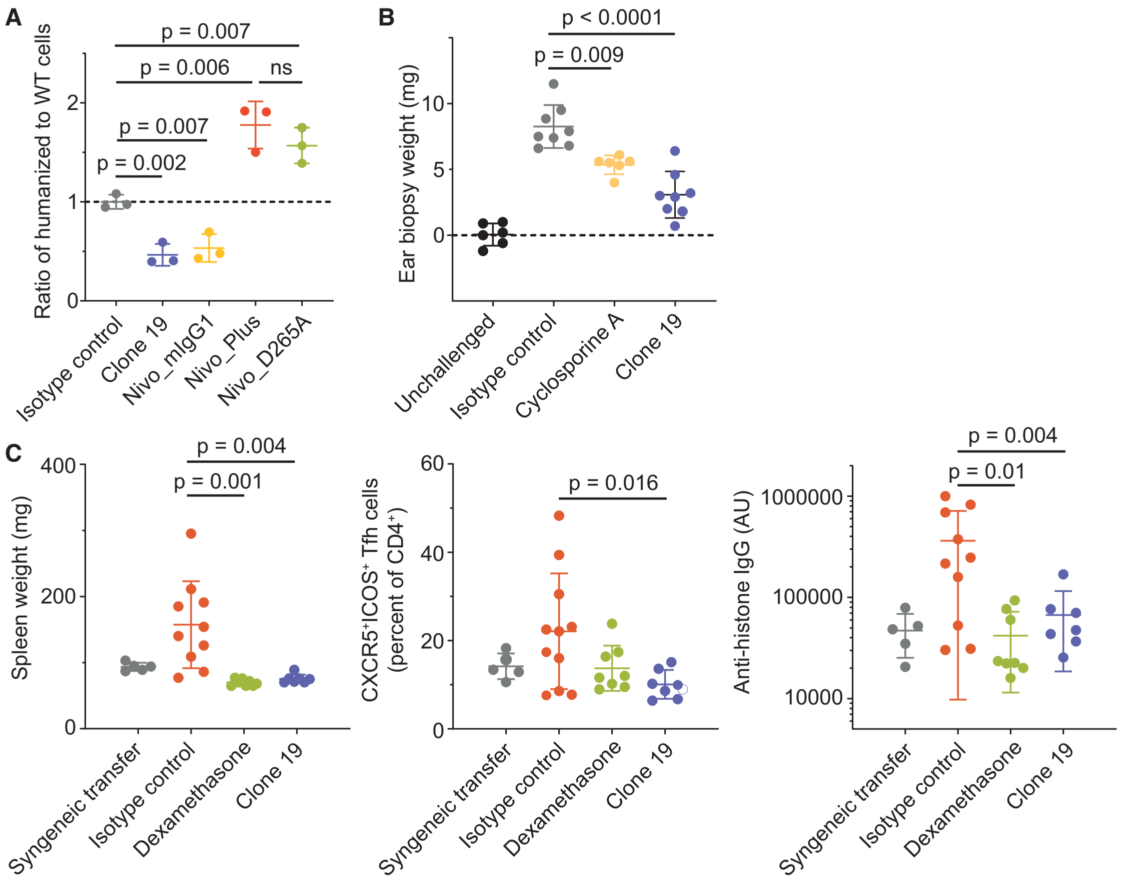

Figure 6. ***In vivo*** **effects of anti-PD-1 antibodies**

(A) Effects of clone 19 and nivolumab derivatives on the antigen-specific expansion of humanized vs. WT PD-1 expressing and non-expressing CD4 T cells from OT-II mice following immunization with ovalbumin. A 50:50 mix of cells was injected into recipient mice on day 0, which were then immunized intraperitoneally (IP) with 100 μg of ovalbumin on day 1 and treated IP with 200 μg of isotype control or PD-1 antibodies on day 2. The ratio of cells in the spleens of the mice on day 8, quantified by flow cytometry and normalized to the average ratio in isotype control treated mice (dashed line), is shown.

(B) Effects of clone 19 on KLH-induced DTH. Mice were immunized with KLH antigen on day 0, 1 h after treatment with anti-PD-1 or isotype control antibody (10 mg/kg), and then challenged intradermally with KLH in one ear on day 5. The difference in biopsy weight between the challenged and unchallenged ear (dashed line) in different treatment groups measured on day 6 is shown.

(C) Effects of clone 19 on the BM12 transfer model of SLE. Splenocytes from huPD-1 mice were transferred IP into BM12 recipient mice, which were then treated with 10 mg/kg anti-PD-1 or isotype control antibody the next day. On day 35, markers of SLE disease severity were assessed including splenomegaly (left), Tfh cell expansion in the spleen quantified by flow cytometry (middle), and serum auto-antibody levels assessed by ELISA (right).

Each point represents an individual mouse. Error bars shown represent SD. For each model, data are representative of two independent experiments. One-way ANOVA with Dunnett's multiple comparison follow-up testing comparing each group to the isotype control, was used for statistical comparisons.

why antibody-induced signaling depends on the dimensions of either the receptor, the antibody, or the RPTPs, or why signaling and RPTP exclusion are strongly correlated. It needs to be emphasized, however, that this explanation for antibody-induced signaling applies only to RPTP-sensitive immune receptors, and not to other classes of receptors, e.g., tumor necrosis factor receptor superfamily proteins that are triggered by local clustering and can be readily agonized with antibodies.[51]

Our CD28- and PD-1-based observations suggest that any receptor whose signaling is antagonized by large RPTPs could in principle be agonized with FcR-engaging, membrane-proximally receptor binding antibodies. Given that there are so many inhibitory immune receptors,[3,52] there could be considerable scope for increasing the "inhibitory pressure" on the immune system in therapeutic settings using antibodies like our anti-PD-1 agonist, clone 19, as was implied by the activity of this antibody *in vivo* in the contexts of both DTH and SLE. The epitope dependence of PD-1-based agonism mirrors, to a degree, the work of Suzuki et al.[20] who found that membrane-proximally binding anti-PD-1 antibodies are agonistic. However, in contrast to Suzuki et al., we found that blocking anti-PD-1 antibodies were also agonistic, albeit more weakly, in a variety of *in vitro* and *in vivo* assays. We would generally expect all antibodies that bind to small receptors or to the membrane-proximal regions of larger receptors to have detectable agonistic activity given sufficiently sensitive assays if the receptors are RPTP sensitive.

The potential negative impact of Fc-FcR interactions on the utility of immune checkpoint blocking antibodies is a matter of considerable clinical importance.[53] Mutated, FcR non-binding,

CellPress
OPEN ACCESS

PD-1-blocking antibodies are more effective in pre-clinical tumor models versus non-depleting, native mIgG1 antibodies, an effect that is inhibitory receptor (FcγR2b) dependent.[18] In a vaccination setting, FcR non-binding anti-PD-1 antibodies support better proliferation of antigen-specific T cells than unmodified mIgG1.[54] Importantly, this effect extends to FcR-humanized mice and PD-1-blocking antibodies of the hIgG4 isotype.[54] For non-depleting antibodies of the type now in clinical use, Dahan et al. attributed this behavior to unexplained, epitope-dependent agonistic signaling by the antibodies,[18] effects that now have an explanation. Nivolumab, produced in the form of mIgG1, while a generally weaker agonist than clone 19, excluded phosphatases and was a potent agonist *in vivo* and in certain settings *in vitro*, an effect ameliorated by lengthening the hinge region of the antibody or preventing FcR binding. The human IgG4 isotype was chosen for the clinical development of the first PD-1-blocking antibodies presumably due to its limited engagement of human Fcγ receptors,[55] but this ability might have been under-estimated. Human IgG4 binds human Fcγ receptors with reasonable affinity (5 μM–30 nM[56]). Moreover, the antibody used in the first-in-human trial of an anti-CD28 agonist, TGN1412, was an IgG4, and FcγR2b promoted its activity.[57] We also found that nivolumab and pembrolizumab IgG4 biosimilars bound a human FcR with sufficient affinity to support their *in vitro* agonistic activity.

PD-1-blocking antibodies are very successful in the clinic, but responses are only seen in a subset of cancer types and a fraction of patients within those types. It seems possible that in some instances when PD-1 blockers are not effective, the failure is due in part to inefficient blockade of the pathway caused by counterproductive antibody agonism, perhaps correlating with the availability of FcRs in the tumor microenvironment. BeiGene has developed a high-affinity hIgG4 PD-1-blocking antibody, Tislelizumab, that does not bind Fcγ receptors and has better efficacy in a tumor xenograft model than an FcR-engaging antibody,[19] and is currently in phase III clinical trials.[58] The results of these trials are eagerly awaited. An additional advantage of this antibody is that it might not be sequestered by macrophage expressed FcRs.[59]

The present work offers a framework for optimizing the design of therapeutic antibodies targeting immune receptors. The signaling principle implies that, for agonists generally, it should be possible to titrate the levels of phosphatase exclusion (and therefore signaling) through the choice of suitable epitopes that alter the "gap" between apposing cells. In this way, the risks associated with over-stimulating activating receptors could be mitigated, allowing this important class of targets to be exploited safely. It is noteworthy that the anti-ICOS agonist presently in clinical trials[11] binds the receptor's ligand-binding site[60] in the manner of weakly-signaling anti-CD28 antibodies,[22,26] perhaps explaining its favorable safety profile. Since it also follows that FcRs would be triggered by engaging the Fc regions of antibodies in this setting,[43,61] it is also an important consideration that agonists may signal bi-directionally, perhaps offering additional therapeutic benefit. For blocking antibodies, the principal goal will be to avoid RPTP exclusion. However, given the strength of tonic signaling by immune checkpoints,[62] it might be helpful to wholly exclude receptors from cell-cell contacts using appropriately engineered antibodies. This fuller understanding of their signaling properties should enhance the safety and broaden and improve the clinical utility of antibodies.

Limitations of the study

We studied RPTP re-organization on model surfaces only and are yet to confirm that it occurs in the context of authentic cell-cell contacts. Importantly, however, our anti-PD-1 antibodies were agonistic *in vivo*, i.e., in the context of physiological receptor expression and bona fide cell-cell contact. That this relied on steric effects of the type demonstrated *in vitro* was indicated by the reduced inhibitory signaling observed when the size of one of our antibodies (Nivo_mIgG) was increased or it was prevented from interacting with FcRs *in vivo*. Although Nivo_mIgG was agonistic *in vivo*, and nivolumab and pembrolizumab biosimilars both suppressed T cell activation in *in vitro* assays utilizing a human FcR, the extent to which blocking antibodies of this type have any agonistic activity in humans is presently uncertain.

STAR★METHODS

Detailed methods are provided in the online version of this paper and include the following:

- KEY RESOURCES TABLE
- RESOURCE AVAILABILITY
  - Lead contact
  - Materials availability
  - Data and code availability
- EXPERIMENTAL MODEL AND STUDY PARTICIPANT DETAILS
  - Cell culture and cell lines
  - Peripheral blood mononuclear cells
  - Mouse strains
- METHOD DETAILS
  - Flow cytometry
  - Lentiviral transduction of cell lines
  - Protein and antibody production
  - PD-1 and clone 19 Fab expression
  - Crystallization
  - Diffraction data collection and processing
  - Structure determination and refinement
  - In vitro stimulation (IL-2 and CD69) assays
  - Calcium release assay
  - Bilayer preparation
  - Imaging T cell contact with glass or bilayers
  - Image analysis
  - Simulations
  - Diffusion analysis
  - Surface plasmon resonance methods
  - Jurkat reporter assay
  - PBMC in vitro activation assay
  - OT-II adoptive transfer assay
  - KLH-induced DTH model
  - BM12 transfer model of SLE
- QUANTIFICATION AND STATISTICAL ANALYSIS
  - Statistical analysis

SUPPLEMENTAL INFORMATION

Supplemental information can be found online at https://doi.org/10.1016/j.immuni.2024.01.007.

ACKNOWLEDGMENTS

The authors are grateful to Heather L. Brouwer and Janet A. Fennelly for technical assistance and to Professor Omer Dushek for advice on data analysis. This study was funded by grants from the United Kingdom Medical Research Council (grant MC_UU-12010/4 to S.J.D. and R.J.C.), the Wellcome Trust (grant 207547/Z/17/Z to S.J.D.), and the Royal Society (Research Professorship RP150066 to D.K.).

AUTHOR CONTRIBUTIONS

The experimental strategy was formulated by S.J.D., D.K., and R.J.C., and the methodology was developed by S.F.L., A.H.L., C.P., E.J., C.A., L.F., and P.S. The experimental work was undertaken and/or the data analyzed by A.H.L., C.P., M.G., M.T.V., J.M.C., E.J., M.F., J.C., S.S., S.M.d.S., B.A., C.A., S.H.M., C.F.J., M.K., U.G., J.L., R.K., M.C., J.H., S.Y., N.A., Y.L., I.W., K.E.A., N.J.R., C.J.L., L.M., and A.M.S. The work was supervised by S.J.D., D.K., R.J.C., P.S., and A.M.S., and funding was acquired by S.J.D., D.K., and R.J.C. The paper was written by S.J.D. with A.H.L. and C.P. and revised by all the authors. R.J.C. and D.K. take responsibility for the *in vivo* and imaging experiments, respectively. S.J.D. conceived the study and is responsible for the *in vitro* work.

DECLARATION OF INTERESTS

S.J.D. is named as an inventor on US and European patents covering the use of anti-PD-1 antibodies. S.J.D., R.J.C., and C.P. were founders of and held equity in MiroBio Ltd, which is now owned by Gilead Sciences. S.J.D. and R.J.C. are paid consultants for Gilead Sciences.

Received: October 11, 2022
Revised: November 30, 2023
Accepted: January 9, 2024
Published: February 13, 2024

REFERENCES

1. Rudd, C.E., Taylor, A., and Schneider, H. (2009). CD28 and CTLA-4 coreceptor expression and signal transduction. Immunol. Rev. *229*, 12–26.
2. Chen, L., and Flies, D.B. (2013). Molecular mechanisms of T cell co-stimulation and co-inhibition. Nat. Rev. Immunol. *13*, 227–242.
3. Dushek, O., Goyette, J., and van der Merwe, P.A. (2012). Non-catalytic tyrosine-phosphorylated receptors. Immunol. Rev. *250*, 258–276.
4. Iwai, Y., Hamanishi, J., Chamoto, K., and Honjo, T. (2017). Cancer immunotherapies targeting the PD-1 signaling pathway. J. Biomed. Sci. *24*, 26.
5. Sharma, P., and Allison, J.P. (2015). The future of immune checkpoint therapy. Science *348*, 56–61.
6. Baumeister, S.H., Freeman, G.J., Dranoff, G., and Sharpe, A.H. (2016). Coinhibitory Pathways in Immunotherapy for Cancer. Annu. Rev. Immunol. *34*, 539–573.
7. Wolchok, J.D. (2015). PD-1 Blockers. Cell *162*, 937.
8. Van Wauwe, J.P., De Mey, J.R., and Goossens, J.G. (1980). OKT3: a monoclonal anti-human T lymphocyte antibody with potent mitogenic properties. J. Immunol. *124*, 2708–2713.
9. Tacke, M., Hanke, G., Hanke, T., and Hünig, T. (1997). CD28-mediated induction of proliferation in resting T cells in vitro and in vivo without engagement of the T cell receptor: evidence for functionally distinct forms of CD28. Eur. J. Immunol. *27*, 239–247.
10. Horvath, C., Andrews, L., Baumann, A., Black, L., Blanset, D., Cavagnaro, J., Hastings, K.L., Hutto, D.L., MacLachlan, T.K., Milton, M., et al. (2012). Storm forecasting: additional lessons from the CD28 superagonist TGN1412 trial. ; author reply 740. Nat. Rev. Immunol. *12*, 740.
11. Hanson, A., Elpek, K., Duong, E., Shallberg, L., Fan, M., Johnson, C., Wallace, M., Mabry, G.R., Sazinsky, S., Pepper, L., et al. (2020). ICOS agonism by JTX-2011 (vopratelimab) requires initial T cell priming and Fc cross-linking for optimal T cell activation and anti-tumor immunity in preclinical models. PLoS One *15*, e0239595.
12. Paluch, C., Santos, A.M., Anzilotti, C., Cornall, R.J., and Davis, S.J. (2018). Immune Checkpoints as Therapeutic Targets in Autoimmunity. Front. Immunol. *9*, 2306.
13. Tuttle, J., Drescher, E., Simón-Campos, J.A., Emery, P., Greenwald, M., Kivitz, A., Rha, H., Yachi, P., Kiley, C., and Nirula, A. (2023). A Phase 2 Trial of Peresolimab for Adults with Rheumatoid Arthritis. N. Engl. J. Med. *388*, 1853–1862.
14. Mayes, P.A., Hance, K.W., and Hoos, A. (2018). The promise and challenges of immune agonist antibody development in cancer. Nat. Rev. Drug Discov. *17*, 509–527.
15. Carter, P.J., and Lazar, G.A. (2018). Next generation antibody drugs: pursuit of the 'high-hanging fruit'. Nat. Rev. Drug Discov. *17*, 197–223.
16. Prasad, V., and Kaestner, V. (2017). Nivolumab and pembrolizumab: Monoclonal antibodies against programmed cell death-1 (PD-1) that are interchangeable. Semin. Oncol. *44*, 132–135.
17. Chamoto, K., Al-Habsi, M., and Honjo, T. (2017). Role of PD-1 in Immunity and Diseases. Curr. Top. Microbiol. Immunol. *410*, 75–97.
18. Dahan, R., Sega, E., Engelhardt, J., Selby, M., Korman, A.J., and Ravetch, J.V. (2015). FcγRs Modulate the Anti-tumor Activity of Antibodies Targeting the PD-1/PD-L1 Axis. Cancer Cell *28*, 285–295.
19. Zhang, T., Song, X., Xu, L., Ma, J., Zhang, Y., Gong, W., Zhang, Y., Zhou, X., Wang, Z., Wang, Y., et al. (2018). The binding of an anti-PD-1 antibody to FcγRIota has a profound impact on its biological functions. Cancer Immunol. Immunother. *67*, 1079–1090.
20. Suzuki, K., Tajima, M., Tokumaru, Y., Oshiro, Y., Nagata, S., Kamada, H., Kihara, M., Nakano, K., Honjo, T., and Ohta, A. (2023). Anti-PD-1 antibodies recognizing the membrane-proximal region are PD-1 agonists that can down-regulate inflammatory diseases. Sci. Immunol. *8*, eadd4947.
21. Davis, S.J., and van der Merwe, P.A. (2006). The kinetic-segregation model: TCR triggering and beyond. Nat. Immunol. *7*, 803–809.
22. Evans, E.J., Esnouf, R.M., Manso-Sancho, R., Gilbert, R.J., James, J.R., Yu, C., Fennelly, J.A., Vowles, C., Hanke, T., Walse, B., et al. (2005). Crystal structure of a soluble CD28-Fab complex. Nat. Immunol. *6*, 271–279.
23. Dennehy, K.M., Kerstan, A., Bischof, A., Park, J.H., Na, S.Y., and Hünig, T. (2003). Mitogenic signals through CD28 activate the protein kinase Ctheta-NF-kappaB pathway in primary peripheral T cells. Int. Immunol. *15*, 655–663.
24. Dennehy, K.M., Elias, F., Na, S.Y., Fischer, K.D., Hünig, T., and Lühder, F. (2007). Mitogenic CD28 signals require the exchange factor Vav1 to enhance TCR signaling at the SLP-76-Vav-Itk signalosome. J. Immunol. *178*, 1363–1371.
25. Levin, S.E., Zhang, C., Kadlecek, T.A., Shokat, K.M., and Weiss, A. (2008). Inhibition of ZAP-70 kinase activity via an analog-sensitive allele blocks T cell receptor and CD28 superagonist signaling. J. Biol. Chem. *283*, 15419–15430.
26. Lühder, F., Huang, Y., Dennehy, K.M., Guntermann, C., Müller, I., Winkler, E., Kerkau, T., Ikemizu, S., Davis, S.J., Hanke, T., et al. (2003). Topological requirements and signaling properties of T cell-activating, anti-CD28 antibody superagonists. J. Exp. Med. *197*, 955–966.
27. Jansson, A., Barnes, E., Klenerman, P., Harlén, M., Sørensen, P., Davis, S.J., and Nilsson, P. (2005). A Theoretical Framework for Quantitative Analysis of the Molecular Basis of Costimulation. J. Immunol. *175*, 1575–1585.
28. Chang, V.T., Fernandes, R.A., Ganzinger, K.A., Lee, S.F., Siebold, C., McColl, J., Jönsson, P., Palayret, M., Harlos, K., Coles, C.H., et al. (2016). Initiation of T cell signaling by CD45 segregation at 'close contacts'. Nat. Immunol. *17*, 574–582.
29. Leupin, O., Zaru, R., Laroche, T., Müller, S., and Valitutti, S. (2000). Exclusion of CD45 from the T-cell receptor signaling area in antigen-stimulated T lymphocytes. Curr. Biol. *10*, 277–280.

CellPress
OPEN ACCESS

30. Tangye, S.G., Phillips, J.H., Lanier, L.L., de Vries, J.E., and Aversa, G. (1998). CD148: a receptor-type protein tyrosine phosphatase involved in the regulation of human T cell activation. J. Immunol. *161*, 3249–3255.

31. Chen, K.Y., Jenkins, E., Körbel, M., Ponjavic, A., Lippert, A.H., Santos, A.M., Ashman, N., O'Brien-Ball, C., McBride, J., Klenerman, D., et al. (2021). Trapping or slowing the diffusion of T cell receptors at close contacts initiates T cell signaling. Proc. Natl. Acad. Sci. USA *118*. e2024250118.

32. Wang, C., Thudium, K.B., Han, M., Wang, X.T., Huang, H., Feingersh, D., Garcia, C., Wu, Y., Kuhne, M., Srinivasan, M., et al. (2014). In vitro characterization of the anti-PD-1 antibody nivolumab, BMS-936558, and in vivo toxicology in non-human primates. Cancer Immunol. Res. *2*, 846–856.

33. Leitner, J., Kuschei, W., Grabmeier-Pfistershammer, K., Woitek, R., Kriehuber, E., Majdic, O., Zlabinger, G., Pickl, W.F., and Steinberger, P. (2010). T cell stimulator cells, an efficient and versatile cellular system to assess the role of costimulatory ligands in the activation of human T cells. J. Immunol. Methods *362*, 131–141.

34. Clynes, R.A., Towers, T.L., Presta, L.G., and Ravetch, J.V. (2000). Inhibitory Fc receptors modulate in vivo cytotoxicity against tumor targets. Nat. Med. *6*, 443–446.

35. Latchman, Y., Wood, C.R., Chernova, T., Chaudhary, D., Borde, M., Chernova, I., Iwai, Y., Long, A.J., Brown, J.A., Nunes, R., et al. (2001). PD-L2 is a second ligand for PD-1 and inhibits T cell activation. Nat. Immunol. *2*, 261–268.

36. Okazaki, T., Maeda, A., Nishimura, H., Kurosaki, T., and Honjo, T. (2001). PD-1 immunoreceptor inhibits B cell receptor-mediated signaling by recruiting src homology 2-domain-containing tyrosine phosphatase 2 to phosphotyrosine. Proc. Natl. Acad. Sci. USA *98*, 13866–13871.

37. Xu, X., Hou, B., Fulzele, A., Masubuchi, T., Zhao, Y., Wu, Z., Hu, Y., Jiang, Y., Ma, Y., Wang, H., et al. (2020). PD-1 and BTLA regulate T cell signaling differentially and only partially through SHP1 and SHP2. J. Cell Biol. *219*, e201905085.

38. Tan, S., Zhang, H., Chai, Y., Song, H., Tong, Z., Wang, Q., Qi, J., Wong, G., Zhu, X., Liu, W.J., et al. (2017). An unexpected N-terminal loop in PD-1 dominates binding by nivolumab. Nat. Commun. *8*, 14369.

39. Na, Z., Yeo, S.P., Bharath, S.R., Bowler, M.W., Balıkçı, E., Wang, C.I., and Song, H. (2017). Structural basis for blocking PD-1-mediated immune suppression by therapeutic antibody pembrolizumab. Cell Res. *27*, 147–150.

40. Akkaya, B. (2012). Modulation of the PD-1 Pathway by Inhibitory Antibody Superagonists (Nuffield Department of Medicine, University of Oxford).

41. Klarquist, J., and Janssen, E.M. (2015). The bm12 Inducible Model of Systemic Lupus Erythematosus (SLE) in C57BL/6 Mice. J. Vis. Exp. e53319.

42. Mittereder, N., Kuta, E., Bhat, G., Dacosta, K., Cheng, L.I., Herbst, R., and Carlesso, G. (2016). Loss of Immune Tolerance Is Controlled by ICOS in Sle1 Mice. J. Immunol. *197*, 491–503.

43. Bakalar, M.H., Joffe, A.M., Schmid, E.M., Son, S., Podolski, M., and Fletcher, D.A. (2018). Size-Dependent Segregation Controls Macrophage Phagocytosis of Antibody-Opsonized Targets. Cell *174*, 131–142.e13.

44. Nimmerjahn, F., and Ravetch, J.V. (2005). Divergent immunoglobulin g subclass activity through selective Fc receptor binding. Science *310*, 1510–1512.

45. Alakoskela, J.-M., Koner, A.L., Rudnicka, D., Köhler, K., Howarth, M., and Davis, D.M. (2011). Mechanisms for Size-Dependent Protein Segregation at Immune Synapses Assessed with Molecular Rulers. Biophys. J. *100*, 2865–2874.

46. Schmid, E.M., Bakalar, M.H., Choudhuri, K., Weichsel, J., Ann, H.S., Geissler, P.L., Dustin, M.L., and Fletcher, D.A. (2016). Size-dependent protein segregation at membrane interfaces. Nat. Phys. *12*, 704–711.

47. Kulenkampff, K., Lippert, A.H., McColl, J., Santos, A.M., Ponjavic, A., Jenkins, E., Humphrey, J., Winkel, A., Franze, K., Lee, S.F., et al. (2020). The Costs of Close Contacts: Visualizing the Energy Landscape of Cell Contacts at the Nanoscale. Biophys. J. *118*, 1261–1269.

48. Jenkins, E., Körbel, M., O'Brien-Ball, C., McColl, J., Chen, K.Y., Kotowski, M., Humphrey, J., Lippert, A.H., Brouwer, H., Santos, A.M., et al. (2023). Antigen discrimination by T cells relies on size-constrained microvillar contact. Nat. Commun. *14*, 1611.

49. Schmitz, K.R., and Ferguson, K.M. (2009). Interaction of antibodies with ErbB receptor extracellular regions. Exp. Cell Res. *315*, 659–670.

50. Feng, Y., Reinherz, E.L., and Lang, M.J. (2018). αβ T Cell Receptor Mechanosensing Forces out Serial Engagement. Trends Immunol. *39*, 596–609.

51. Dadas, O., Ertay, A., and Cragg, M.S. (2023). Delivering co-stimulatory tumor necrosis factor receptor agonism for cancer immunotherapy: past, current and future perspectives. Front. Immunol. *14*, 1147467.

52. Sinclair, N.R. (1999). Why so many coinhibitory receptors? Scand. J. Immunol. *50*, 10–13.

53. Chen, X., Song, X., Li, K., and Zhang, T. (2019). FcγR-Binding Is an Important Functional Attribute for Immune Checkpoint Antibodies in Cancer Immunotherapy. Front. Immunol. *10*, 292.

54. Moreno-Vicente, J., Willoughby, J.E., Taylor, M.C., Booth, S.G., English, V.L., Williams, E.L., Penfold, C.A., Mockridge, C.I., Inzhelevskaya, T., Kim, J., et al. (2022). Fc-null anti-PD-1 monoclonal antibodies deliver optimal checkpoint blockade in diverse immune environments. J. Immunother. Cancer *10*, e003735.

55. Jiang, X.R., Song, A., Bergelson, S., Arroll, T., Parekh, B., May, K., Chung, S., Strouse, R., Mire-Sluis, A., and Schenerman, M. (2011). Advances in the assessment and control of the effector functions of therapeutic antibodies. Nat. Rev. Drug Discov. *10*, 101–111.

56. Bruhns, P., Iannascoli, B., England, P., Mancardi, D.A., Fernandez, N., Jorieux, S., and Daëron, M. (2009). Specificity and affinity of human Fcgamma receptors and their polymorphic variants for human IgG subclasses. Blood *113*, 3716–3725.

57. Hussain, K., Hargreaves, C.E., Roghanian, A., Oldham, R.J., Chan, H.T., Mockridge, C.I., Chowdhury, F., Frendéus, B., Harper, K.S., Strefford, J.C., et al. (2015). Upregulation of FcγRIIb on monocytes is necessary to promote the superagonist activity of TGN1412. Blood *125*, 102–110.

58. Shen, L., Kato, K., Kim, S.B., Ajani, J.A., Zhao, K., He, Z., Yu, X., Shu, Y., Luo, Q., Wang, J., et al. (2022). Tislelizumab Versus Chemotherapy as Second-Line Treatment for Advanced or Metastatic Esophageal Squamous Cell Carcinoma (RATIONALE-302): A Randomized Phase III Study. J. Clin. Oncol. *40*, 3065–3076.

59. Arlauckas, S.P., Garris, C.S., Kohler, R.H., Kitaoka, M., Cuccarese, M.F., Yang, K.S., Miller, M.A., Carlson, J.C., Freeman, G.J., Anthony, R.M., et al. (2017). In vivo imaging reveals a tumor-associated macrophage-mediated resistance pathway in anti-PD-1 therapy. Sci. Transl. Med. *9*, eaal3604.

60. Rujas, E., Cui, H., Sicard, T., Semesi, A., and Julien, J.P. (2020). Structural characterization of the ICOS/ICOS-L immune complex reveals high molecular mimicry by therapeutic antibodies. Nat. Commun. *11*, 5066.

61. Felce, J.H., Sezgin, E., Wane, M., Brouwer, H., Dustin, M.L., Eggeling, C., and Davis, S.J. (2018). CD45 exclusion– and cross-linking–based receptor signaling together broaden FcεRI reactivity. Sci. Signal. *11*, eaat0756.

62. Fernandes, R.A., Su, L., Nishiga, Y., Ren, J., Bhuiyan, A.M., Cheng, N., Kuo, C.J., Picton, L.K., Ohtsuki, S., Majzner, R.G., et al. (2020). Immune receptor inhibition through enforced phosphatase recruitment. Nature *586*, 779–784.

63. Yagüe, J., White, J., Coleclough, C., Kappler, J., Palmer, E., and Marrack, P. (1985). The T cell receptor: the alpha and beta chains define idiotype, and antigen and MHC specificity. Cell *42*, 81–87.

64. Liu, C.P., Parker, D., Kappler, J., and Marrack, P. (1997). Selection of Antigen-specific T Cells by a Single IEk Peptide Combination. J. Exp. Med. *186*, 1441–1450.

65. White, J., O'Brien, R.L., and Born, W.K. (2020). BW5147 and Derivatives for the Study of T Cells and their Antigen Receptors. Arch. Immunol. Ther. Exp. (Warsz) *68*, 15.

66. Weiss, A., Wiskocil, R.L., and Stobo, J.D. (1984). The role of T3 surface molecules in the activation of human T cells: a two-stimulus requirement for IL 2 production reflects events occurring at a pre-translational level. J. Immunol. *133*, 123–128.

67. Barnden, M.J., Allison, J., Heath, W.R., and Carbone, F.R. (1998). Defective TCR expression in transgenic mice constructed using cDNA-based alpha- and beta-chain genes under the control of heterologous regulatory elements. Immunol. Cell Biol. *76*, 34–40.

68. Yang, H., Rei, M., Brackenridge, S., Brenna, E., Sun, H., Abdulhaqq, S., Liu, M.K.P., Ma, W., Kurupati, P., Xu, X., et al. (2021). HLA-E-restricted, Gag-specific $CD8^+$ T cells can suppress HIV-1 infection, offering vaccine opportunities. Sci. Immunol. *6*, eabg1703.

69. Cheng, X., Veverka, V., Radhakrishnan, A., Waters, L.C., Muskett, F.W., Morgan, S.H., Huo, J., Yu, C., Evans, E.J., Leslie, A.J., et al. (2013). Structure and interactions of the human programmed cell death 1 receptor. J. Biol. Chem. *288*, 11771–11785.

70. Bokhove, M., Sadat Al Hosseini, H., Saito, T., Dioguardi, E., Gegenschatz-Schmid, K., Nishimura, K., Raj, I., de Sanctis, D., Han, L., and Jovine, L. (2016). Easy mammalian expression and crystallography of maltose-binding protein-fused human proteins. J. Struct. Biol. *194*, 1–7.

71. Chang, V.T., Crispin, M., Aricescu, A.R., Harvey, D.J., Nettleship, J.E., Fennelly, J.A., Yu, C., Boles, K.S., Evans, E.J., Stuart, D.I., et al. (2007). Glycoprotein structural genomics: solving the glycosylation problem. Structure *15*, 267–273.

72. McCoy, A.J., Grosse-Kunstleve, R.W., Adams, P.D., Winn, M.D., Storoni, L.C., and Read, R.J. (2007). Phaser crystallographic software. J. Appl. Crystallogr. *40*, 658–674.

73. Murshudov, G.N., Vagin, A.A., and Dodson, E.J. (1997). Refinement of Macromolecular Structures by the Maximum-Likelihood Method. Acta Crystallogr. D Biol. Crystallogr. *53*, 240–255.

74. Emsley, P., and Cowtan, K. (2004). Coot: model-building tools for molecular graphics. Acta Crystallogr. D Biol. Crystallogr. *60*, 2126–2132.

75. Langer, G., Cohen, S.X., Lamzin, V.S., and Perrakis, A. (2008). Automated macromolecular model building for X-ray crystallography using ARP/wARP version 7. Nat. Protoc. *3*, 1171–1179.

76. Fritzsche, M., Fernandes, R.A., Colin-York, H., Santos, A.M., Lee, S.F., Lagerholm, B.C., Davis, S.J., and Eggeling, C. (2015). CalQuo: automated, simultaneous single-cell and population-level quantification of global intracellular Ca2+ responses. Sci. Rep. *5*, 16487.

77. Edelstein, A.D., Tsuchida, M.A., Amodaj, N., Pinkard, H., Vale, R.D., and Stuurman, N. (2014). Advanced methods of microscope control using μManager software. J. Biol. Methods *1*, e10.

78. Harris, C.R., Millman, K.J., van der Walt, S.J., Gommers, R., Virtanen, P., Cournapeau, D., Wieser, E., Taylor, J., Berg, S., Smith, N.J., et al. (2020). Array programming with NumPy. Nature *585*, 357–362.

79. van der Walt, S., Schönberger, J.L., Nunez-Iglesias, J., Boulogne, F., Warner, J.D., Yager, N., Gouillart, E., and Yu, T.; scikit-image contributors (2014). scikit-image: image processing in Python. PeerJ *2*, e453.

80. Sage, D., Pham, T.A., Babcock, H., Lukes, T., Pengo, T., Chao, J., Velmurugan, R., Herbert, A., Agrawal, A., Colabrese, S., et al. (2019). Super-resolution fight club: assessment of 2D and 3D single-molecule localization microscopy software. Nat. Methods *16*, 387–395.

81. Weimann, L., Ganzinger, K.A., McColl, J., Irvine, K.L., Davis, S.J., Gay, N.J., Bryant, C.E., and Klenerman, D. (2013). A quantitative comparison of single-dye tracking analysis tools using Monte Carlo simulations. PLoS One *8*, e64287.

82. Persson, F., Lindén, M., Unoson, C., and Elf, J. (2013). Extracting intracellular diffusive states and transition rates from single-molecule tracking data. Nat. Methods *10*, 265–269.

83. Austin, J.W., Lu, P., Majumder, P., Ahmed, R., and Boss, J.M. (2014). STAT3, STAT4, NFATc1, and CTCF regulate PD-1 through multiple novel regulatory regions in murine T cells. J. Immunol. *192*, 4876–4886.

STAR★METHODS

KEY RESOURCES TABLE

REAGENT or RESOURCE	SOURCE	IDENTIFIER
Antibodies		
Anti-DAM (goat anti-donkey antibody) FITC conjugated	Bethyl Laboratories	Cat# A140-128F
Anti-HA-11 clone 16B12 PE conjugated	Biolegend	Cat# 901518
Anti-hCD28 clone CD28.2	Biolegend	Cat# 302934
Anti-hCD3 clone OKT3	Biolegend	Cat# 317326
Anti-hFc clone IC10 F890	Gift (Dr JR Young, Institute for Animal Health, Compton)	N/A
Anti-hPD-1 clone 19 and humanized clone 19	Made in-house	N/A
Anti-hPD-1 clone 2	Made in-house	N/A
Anti-hPD-1 nivolumab mIgG1	Absolute Antibody	Custom order
Anti-hPD-1 nivolumab mIgG1 + 50 aa mCD43	Absolute Antibody	Custom order
Anti-mCD3 clone KT3	Bio-Rad Laboratories Ltd	Cat# MCA500EL
Anti-mCD45 YW62.3.20 antibody fragment	Gift (Prof H Waldmann, Oxford)	N/A
Anti-mCD69 clone H1.2F3 PE-Cy7 conjugated	Biolegend	Cat# 104511
Anti-PDL1 clone 29E.2A3	Biolegend	Cat# 329745
Anti-PDL1 clone A20050B	Biolegend	Cat# 947804
Anti-rCD28 clone JJ316	BD Biosciences	Cat# 554992
Anti-rCD28 clone JJ316 + 30 aa mCD43	Absolute Antibody	N/A (custom order)
Anti-rCD28 clone JJ316 + 50 aa mCD43	Absolute Antibody	N/A (custom order)
Anti-rCD28 clone JJ319	Invitrogen	Cat# 16-0280-85
Anti-rCD28 clone JJ319 PE conjugated	eBioscience	Cat# 12-0280-83
Donkey anti-mIgG H+L	Jackson ImmunoResearch	Cat# 715-001-003
Human recombinant IL6	Biolegend	Cat# 570802
Mouse IgG1 Isotype control, clone MOPC-21	Biolegend	Cat# 400193
Bacterial and virus strains		
One Shot Top10 chemically competent *E. coli*	Invitrogen	Cat# C404010
Biological samples		
Human PBMCs were isolated from NHS Blood and Transplant (NHSBT) Service NCI leukocyte cones by Ficoll-Paque density gradient centrifugation	NHSBT Service Non-clinical issue	N/A
Chemicals, peptides, recombinant proteins and lipids		
96 well optical plate CVG sterile w/lid white	Thermo Scientific	Cat# 164590
Alexa Fluor 647 Antibody Labeling Kit	Invitrogen	Cat# A20186
Amicon Ultra-15 centrifugal filters	Merck, Millipore	Cat# UFC901096
Ampicillin	Sigma	Cat# A9518-25G
BD Cytofix/ Cytoperm Fixation/ Permeabilization Kit	BD Biosciences	Cat# 554714
BD Quantibrite Beads PE Quantification Kit	BD Biosciences	Cat# 340495
Bio-Glo Luciferase Assay Reagent	Promega	Cat# G7940
Corning 500mL Vacuum Filter/Storage Bottle System 0.22 μm pore 33.2 cm^2 PES membrane	Corning	Cat# 431097
Cover glasses, N°1	VWR International (Lutterworth, UK)	Size 1
CultureWell, chambered cover glass	Grace Bio Labs	CWCS-50R-1.0, 103350
Dexamethasone	Acros Organics	Cat# 230302500

(*Continued on next page*)

Continued

REAGENT or RESOURCE	SOURCE	IDENTIFIER
DGS-NTA(Ni) (1,2-dioleoyl-sn-glycero-3-[(N-(5-amino-1-carboxypentyl)iminodiacetic acid) succinyl] (nickel salt))	Avanti Polar Lipids	Cat #790404C
DMEM medium (for growing CHO-K1 cells)	Gibco	Cat# 10938-025
DMEM medium (for growing HEK293T cells)	Sigma	Cat# D5976
DMSO	New England Biolabs	Cat# B0515A
EIA/RIA Plate 96 well. No lid. Clear. Flat bottom. High Binding polystyrene	Costar	Cat# 3690
Express PES membrane filter Unit, 0.22 μm filter	Millipore	Cat# SLGP033RS
Filtropur S, 0.45 μm	Starsted	Cat# 83.1836
Fluo-4, AM, cell permeant	Invitrogen	Cat# F14201
Foetal Bovine Serum	Gibco	Cat# 10500-064
GeneJuice transfection reagent 5x 1mL	Merck	Cat# 70967-6
HBS-EP+ Buffer 10x	Cytiva	Cat# BR100669
HBS-P+ Buffer 10x	Cytiva	Cat# BR100671
Hepes-NaOH, 1 M	Sigma	Cat# H0887-100ML
Imidazole	Acros Organics	Cat# 122020020
Ionomycin	Sigma	Cat# I0634
Keyhole limpet hemocyanin (KLH)	Sigma	Cat# H7017
L-glutamine, 200 mM	Sigma	Cat# G7513-100ML
LB agar	Sigma	Cat# L7025-500TAB
LB broth	Sigma	Cat# L7275-500TAB
Methionine sulphoximine	Sigma	Cat# M5379-250MG
Mouse Uncoated IL-2 ELISA Kit	Invitrogen	Cat# 88-7024-88
Ni-NTA agarose	Qiagen	Cat# 30230
Nickel(II) sulfate hexahydrate	Sigma	Cat# N4882-250G
NTA Reagent Kit	Cytiva	Cat# 28995043
Optical glass-bottom dishes	World Precision Instruments	Cat# FD3510-100
Ovalbumin	Merck	Cat# A5378
Penicillin (5,000 U)/Streptomycin (5 mg)/Neomycin (10 mg)	Sigma	Cat# P4083-100ML
Phosphate buffered saline	Oxoid	Cat# BR0014G
POPC (1-palmitoyl-2-oleoyl-glycero-3-phosphocholine)	Avanti Polar Lipids	Cat# 850457C
Probenecid	Invitrogen	Cat# P36400
PureLink HiPure Plasmid Miniprep Kit	Invitrogen	Cat# K210003
RPMI Medium 1640	Gibco	Cat# 21875-034
Series S Sensor Chip NTA	Cytiva	Cat# 28994951
Series S Sensor Chip Protein A	Cytiva	Cat# 29127555
Sodium chloride	Sigma	Cat# S9888-1KG
Sodium pyruvate, 100 mM	Sigma	Cat# S8636-100ML
Tris-HCl pH 8.0, 1 M	Sigma	Cat# T3038-1L
Trypsin solution from porcine pancreas	Sigma	Cat# T4549-100ML
Rat-mouse CD28Fc		Insert sequence in Table S4
Full-length rat-mouse CD28 chimera		Insert sequence in Table S4
Truncated rat-mouse CD28 chimera		Insert sequence in Table S4
Rat-mouse CD45RABCLck chimeric form of mouse Lck		Insert sequence in Table S4
Transmembrane-anchored form of mouse Lck		Insert sequence in Table S4
Full-length rat-human-mouse FcCD28 chimera		Insert sequence in Table S4
Full-length rat-mouse CD45 chimera		Insert sequence in Table S4
Truncated mouse CD45 chimera		Insert sequence in Table S4

(*Continued on next page*)

CellPress
OPEN ACCESS

Continued		
REAGENT or RESOURCE	SOURCE	IDENTIFIER
Full-length rat-mouse CD148 chimera		Insert sequence in Table S4
Truncated rat-mouse CD148 chimera		Insert sequence in Table S4
Mouse sFcγR2b		Insert sequence in Table S4
mEOS3.2-tagged truncated chimeric rat-mouse CD28		Insert sequence in Table S4
Human-mouse PD-1-CD28 chimera		Insert sequence in Table S4
Anti-CD3 "T cell stimulator"		Insert sequence in Table S4
Full-length human PD-L1		Insert sequence in Table S4
Full-length mouse FcγR2b		Insert sequence in Table S4
Full-length human PD-1		Insert sequence in Table S4
Full-length human FcγR2b		Insert sequence in Table S4
Nivolumab H chain variable domain		Insert sequence in Table S4
Nivolumab κ chain variable domain		Insert sequence in Table S4
Mouse κ chain constant domain		Insert sequence in Table S4
Mutated (D265A) mouse IgG1 H chain constant region		Insert sequence in Table S4
Extended hinge mouse IgG1 H chain constant region		Insert sequence in Table S4
Full-length human SHP2 linked to HaloTag		Insert sequence in Table S4
Full-length human PD-1		Insert sequence in Table S4
Human PD-1 comprising a 6 aa long cytosolic tail		Insert sequence in Table S4
Human PD-1 with mutated ITIM and ITSM sequences (Y>F)		Insert sequence in Table S4
Human sPD-L1		Insert sequence in Table S4
Experimental models: Cell lines		
Hamster: CHO-K1	Lonza	N/A
Human: HEK293T	ATCC	ATCC CRL-3216
Human: Jurkat NFAT luciferase reporter cells expressing PD-1	Promega	Cat# J115A
Mouse: Yae5b3k	Gift (Prof P Marrack, Boulder)	N/A
Mouse: BW5147	ATCC	ATCC CRL-1588
Mouse: DO11.10	Gift (Prof P Marrack, Boulder)	N/A
Experimental models: Organisms/strains		
BM12 mice (B6(C)-H2-Ab1^{bm12}/KhEgJ)	The Jackson Laboratory	Strain # 001162
Humanized PD-1 mice on the C57BL/6 background (pdcd1^{tm1606Arte})	Taconic Biosciences	N/A
OT-II mice (B6.Cg-Tg(TcraTcrb)425Cbn/J)	The Jackson Laboratory	Strain # 004194
UBC-GFP mice (C57BL/6-Tg(UBC-GFP) 30Scha/J)	The Jackson Laboratory	Strain # 004353
Oligonucleotides		
pEYFP reverse: ACCAGGATGGGCACCAC	IDT; Sigma	Lab ID: 554
pHR forward: TGCTTCTCGCTTCTGTTCG	IDT; Sigma	Lab ID: 1166
pHR reverse: CCACATAGCGTAAAAGGAGC	IDT; Sigma	Lab ID: 1167
pHRi forward: CAACAAGTTACCGAGAAAG AAGAACTCAC	IDT; Sigma	Lab pHRi-F-mHSP
Recombinant DNA		
P8.91	Addgene	ID 187441
pEE14	Lonza	N/A
pHRi backbone	Addgene	ID 187602
pHRSin backbone	Addgene	ID 187353
pHRSin_IRESEmGFP backbone	Addgene	ID 187358
pMDG	Addgene	ID 187440

(*Continued on next page*)

Continued		
REAGENT or RESOURCE	SOURCE	IDENTIFIER
Software and algorithms		
Adobe Illustrator v26.4.1	Adobe	https://www.adobe.com/products/illustrator.html
ARP/wARP	EMBL	https://www.embl-hamburg.de/ARP/
Coot	MRC LMB	https://www2.mrc-lmb.cam.ac.uk/personal/pemsley/coot/
DIALS		https://dials.diamond.ac.uk/installation.html
FlowJo v10.8.1		https://www.flowjo.com/
GraphPad Prism v9.3.1	GraphPad	https://www.graphpad.com/
MATLAB R2022a	MathWorks	https://uk.mathworks.com/products/matlab.html
Phaser crystallographic software	Cambridge Institute for Medical Research	https://www.phaser.cimr.cam.ac.uk/index.php/Phaser_Crystallographic_Software
PyMOL v2.5.2	Schrödinger	https://pymol.org/2/
R studio		http://www.rstudio.com/
REFMAC5	MRC Laboratory of Molecular Biology	https://www2.mrc-lmb.cam.ac.uk/groups/murshudov/content/refmac/refmac.html
SnapGene v4.1.9	GSL Biotech	https://www.snapgene.com/
UCSF Chimera v1.16	UCSF Resource for Biocomputing, Visualization, and Informatics	https://www.rbvi.ucsf.edu/chimera/
Other		
DNA sequencing	Source Bioscience	N/A

RESOURCE AVAILABILITY

Lead contact

Further information and requests for resources and reagents should be directed to the lead contact Simon Davis (simon.davis@imm.ox.ac.uk).

Materials availability

Humanized PD-1 mice are currently exclusively licenced to MiroBio Ltd but may be available under a material transfer agreement upon agreement with MiroBio Ltd. All other newly generated materials described in this manuscript, or sequences required for their recombinant production, are available upon request.

Data and code availability

Structural data for the PD1-Fab complex have been deposited at RCSB PDB (PDB number 8EQ6) and are publicly available as of the date of publication. Raw flow cytometry, ELISA, surface plasmon resonance and microscopy data reported in this paper will be shared by the lead contact upon request. All other data are available in the main text and supplementary materials. Any additional information required to reanalyze the data reported in this paper is available from the lead contact upon request.

EXPERIMENTAL MODEL AND STUDY PARTICIPANT DETAILS

Cell culture and cell lines

Cell lines were grown at 37 °C in a 5% CO_2 atmosphere. Mouse leukemia T cell hybridomas DO11.10[63] and Yae5b3k,[64] the mouse thymoma BW5147,[65] and human Jurkat T cells[66] were cultured in Joklik-modified Minimum Essential Medium (JMEM; supplemented with 10% fetal calf serum (FCS), 10 mM HEPES, 1 mM sodium pyruvate, 2 mM L-glutamine, and antibiotics (50 units penicillin, 50 μg streptomycin, and 100 μg neomycin per mL)) and kept at a density between $1x10^5$ and $1x10^6$ cells per mL. Human embryonic kidney (HEK)-293T cells were grown in Dulbecco's MEM (DMEM, Gibco) supplemented with 10% FCS, 2 mM glutamine, and antibiotics (50 units penicillin and 50 μg streptomycin per mL). B-cell hybridomas for antibody production were cultured in Roswell Park Memorial Institute (RPMI)-1640 medium supplemented with 15% FCS, 10 mM HEPES, 1 mM sodium pyruvate, 2 mM L-glutamine, 100 μM hypoxanthine, 16 μM thymidine supplement, 100 μM non-essential amino acids, and antibiotics (50 units penicillin, 50 μg

streptomycin, and 100 μg neomycin per mL). When collecting supernatant for antibody purification, the FCS was replaced with 10% ultra-low-IgG FCS (Gibco). Primary human PBMCs were cultured in RPMI-1640 medium supplemented with 10% FCS, 10 mM HEPES, 1 mM sodium pyruvate, 2 mM L-glutamine, 100 μM non-essential amino acids, and antibiotics (50 units penicillin, 50 μg streptomycin, and 100 μg neomycin per mL).

Peripheral blood mononuclear cells

PBMCs were isolated from NHSBT Service NCI leukocyte cones by Ficoll-Paque density gradient centrifugation. No donor information was provided.

Mouse strains

Humanized PD-1 mice on the C57BL/6 background were produced by Taconic Biosciences as described in Figure S7A (and Akkaya[40]). These mice were also crossed onto OT-II TCR transgenic mice, which express a TCR specific for an ovalbumin peptide presented by MHC class II,[67] and therefore develop a CD4 T cell compartment greatly enriched for ovalbumin specific cells.

BM12 mice were obtained from The Jackson Laboratory.

All animal experiments were carried out in accordance with Animal (Scientific Procedures) Act 1986, with procedures reviewed by the Oxford University Animal Welfare and Ethical Review Body, and conducted under project licence PPL P79A4C5BA. Animals were housed in specific pathogen free conditions. All animals were housed in individually ventilated cages in social groups, provided with food and water ad-libitum and maintained on a 12 h light /12 h dark cycle (150–200 lux cool white LED light, measured at the cage floor). Adult mice (>8 weeks of age) were used for experiments, with gender and age matched between treatment groups as closely as possible. For cell transfer experiments donor and recipient mice of the same sex were used.

METHOD DETAILS

Flow cytometry

Fluorescence-activated cell sorting (FACS) was performed on BD FACS Celesta or Cyan APP (Dako) flow cytometers and analyzed using FlowJo software. Typically, cells were pelleted by centrifugation (180*g* RCF for 2') in 96 well U-bottom plates, washed with FACS buffer (phosphate-buffered saline (PBS), 2% FCS + 2 mM EDTA + 0.1% sodium azide), and then stained for 30' on ice by resuspension in 25 μl of antibody staining cocktail. Staining cocktails consisted of appropriate dilutions of each dye-conjugated antibody in FACS buffer. An anti-mCD16/32 antibody (Biolegend) was added at 1:50 dilution to block Fc receptor binding. Zombie NIR Fixable Viability Dye (Biolegend) was used as a live/dead marker. When secondary staining was required, cells were washed twice in FACS buffer prior to resuspension in secondary antibody stain for 30′ at 4 °C. Cells were then washed and either resuspended in FACS buffer for immediate data acquisition or resuspended in 2% paraformaldehyde/PBS-azide for fixation.

Lentiviral transduction of cell lines

Constructs (Table S4) were cloned into the pHR-SIN lentivirus expression vector or pSF-Lenti-SFFV-EMCV-Blast-SV40ori (OxGene). Lentivirus was generated by transiently transfecting HEK-293T cells grown in DMEM supplemented with antibiotics and L-glutamine, with the pMD.G and p8.91 packaging vectors and each of the pHR-SIN constructs, using Genejuice (Merck) according to the manufacturer's instructions. For constructs cloned into the pSF-Lenti vector, transfections were performed using ExceLenti LTX Lentivirus Packaging Mix (OxGene) with Lipofectamine 3000 (Invitrogen). Forty-eight hours later the virus was harvested and the HEK 293T cells removed by centrifugation at 3,000*g* for 5'. The supernatant, containing virus, was filtered with a 0.45 μL syringe filter and added to the relevant cells for infection. The following day the medium was replaced with fresh JMEM. After 5-7 days of culturing, expression of the gene of interest was examined by flow cytometry and the cells either used immediately or frozen. Prior to use, expression levels were confirmed by flow cytometry and, where necessary, quantitated using Quantibrite Beads (BD Biosciences). For some cell lines, stable cell clones were produced by limiting dilution cloning in conjunction with expression screening by flow cytometry. The TCR-deficient Jurkat cell line used for expression of PD-1 constructs was made using the LentiCRISPRV2 plasmid, as described in Yang et al.[68]

Protein and antibody production

Rat CD28Fc (Table S4), and PD-1Fc and soluble forms of PD-L1 and PD-L2 were expressed in Chinese hamster ovary-K1 (CHO-K1) cells using approaches described previously.[69] The C-terminally His$_6$-spacer-His$_6$ ("spacer-His") tagged sequences of the mFcγR2b and PD-L1 ECDs (yielding sFcR and sPD-L1, respectively; Table S4) were cloned into the pHR-SIN vector which was then used to generate lentiviruses as described in the preceding section. CHO-K1 cells were seeded into T75 flasks (Sigma-Aldrich) at 4x10^6 cells/mL in 16 mL DMEM supplemented with 10% FCS, 1 mM sodium pyruvate, 2 mM L-glutamine, 2% nucleoside, and amino acid supplement (0.35 mg/mL adenosine, 0.35 mg/mL cytidine, 0.35 mg/mL guanosine, 0.35 mg/mL uridine, 0.12 mg/mL thymidine, 3.1 mg/mL asparagine, 3.05 mg/mL glutamic acid, 1.78 mg/mL alanine, 2.65 mg/mL aspartic acid, and 2.3 mg/mL proline) and antibiotics. The CHO-K1 cell culture supernatant was discarded and replaced with the filtered virus-containing supernatant from the HEK 293T cells. Spacer-His tagged PD-L1 was made by adding lentivirus-containing supernatant to 2x10^6 fresh HEK 293T cells seeded into a T75 flask. The following day, the virus-containing cell culture supernatant was discarded and replaced with fresh medium as above. On Day 3, the cells (CHO-K1 or HEK 293T) were washed with PBS and trypsinized with 5 mL of 1x trypsin in PBS (from 50x Trypsin

Solution, Sigma-Aldrich) for 3-5' at 37 °C. Ten milliliters of medium were then added, and cells were harvested at 1200*g* for 5'. Cells were resuspended in 50 mL of medium supplemented as above and seeded into a T175 flask (Greiner flask, tissue culture treated, Sigma-Aldrich) and cultured. Supernatant was recovered and replaced every 2-3 days until 1 L had been collected. The protein was purified from the filtered supernatant using nickel-chelation (Ni-NTA agarose, QIAGEN) and fast protein liquid chromatography (FPLC) using a Superdex 200 HR column on an ÄKTA FPLC system (Cytiva).

Engineered variants of nivolumab and anti-CD28 antibodies, extended in the hinge region via insertions of 30 N-terminal residues of mCD43 ECD sequence (RTTMLPSTPHITAPSTSEAQNASPSVSVGS) or 50 N-terminal residues of CD43 ECD sequence (RTTMLPSTPHITAPSTSEAQNASPSVSVGSGTVDSKETISPWGQTTIPVS), were produced in HEK 293 cells by Absolute Antibody Ltd. Nivolumab and pembrolizumab hIgG4 biosimilars were obtained from MedChemExpress. mIgG1 isotype control antibody clone MOPC-21 was purchased from Biolegend. Anti-PD-1 antibodies clone 19 and clone 2 were purified from hybridoma supernatant using protein-G coupled Sepharose (Sigma-Aldrich) and FPLC. Fab fragments were prepared by digestion in 20 mM sodium phosphate, 10 mM EDTA, 20 mM cysteine-HCl pH 7.0, with immobilized papain slurry (Thermo Fisher). The Fab fragments were labeled as required with Alexa Fluor 488 or Alexa Fluor 647 dyes using Molecular Probes Antibody Labeling Kits (Thermo Fisher). Soluble, monomeric PD-1 was generated by proteolytic cleavage at a thrombin site introduced into PD-1Fc at the base of the stalk region of PD-1 (i.e., adjacent to the transmembrane region).

PD-1 and clone 19 Fab expression

A PD-1 ECD expression construct, comprising cDNA encoding residues 2-127 of the mature polypeptide was cloned into pcDNA3.4, alongside sequence encoding, N-terminally, the CD33 signal peptide and Maltose Binding Protein (MBP)[70] and, C-terminally, a PreScission protease site (Cytivia) and 10x histidine tag. To aid expression of soluble monomers, Cys93 of the ECD was mutated to Ser. Protein was expressed using the FreeStyle 293 Expression System (Thermo Fisher), supplemented with 1 μg/ml kifuensine (Abcam), to allow deglycosylation of the protein.[71] HEK293F cells were harvested after 3 days, and cells removed from the medium by centrifugation at 4000 rpm, following which the medium was diluted 1:1 with PBS and filtered using a 0.2 μm filter. Filtered medium was loaded onto a HISTRAP HP (Cytiva) column using an ÄKTA Start system pre-equilibrated with PBS. Following a 40 mM imidazole wash, histidine-tagged proteins were eluted using 500 mM imidazole and loaded onto a HiLoad 16/600 Superdex 200pg size exclusion chromatography (SEC) column in PBS. After SEC, the purified protein was digested with EndoHf (New England Biolabs). Following EndoHf digestion, the protein was re-purified as described above to remove the enzyme. The N-terminal MBP fusion and 10x-histidine tag were removed by overnight cleavage with PreScission protease. The digested MBP-his-PD-1 protein was passed over a combination of HISTRAP HP and GSTrap columns to deplete cleaved MBP-HIS, un-cleaved MBP-HIS-PD1 and the GST-tagged PreScission protease. The eluant was concentrated using 3K MWCO centrifuge filters and loaded onto a HiLoad 16/600 Superdex 200pg column. Following elution, the protein was concentrated as required using a 3K MWCO centrifuge filter (Thermo Fisher).

Sequences encoding chain-specific rabbit signal peptides and the humanized clone 19 variable domains with IgG1 constant domains were expressed from the Antibody-Expressing Positive Control Vector (Thermo Fisher), using the Gibco ExpiCHO Expression System Kit (Thermo Fisher). ExpiCHO cell medium was harvested after 10 days, cells removed by centrifugation at 4000 rpm, antibiotics added, and then filtered through 0.45 and 0.2μm filters. Filtered medium was loaded onto a MabSelect SuRe column (Thermo Fisher) using ÄKTA start system pre-equilibrated with PBS. Antibodies were eluted using 0.1 M citrate pH 3.2 and loaded onto a HiLoad 16/600 Superdex 200pg column in PBS. To produce Fab fragments, the purified antibody was digested using Immobilized Papain Agarose Resin (Thermo Fisher). The digested antibody was applied to a MabSelect SuRe column to remove cleaved Fc and un-cleaved IgG. The eluant was concentrated using 10K MWCO centrifuge filters and loaded onto a HiLoad 16/600 Superdex 200pg column prior to final concentration using a 10K MWCO centrifuge filter.

The Fab-PD-1 complex was generated by mixing the soluble PD-1 with Fab at a ~2:1 molar ratio and incubated at 4°C overnight. The complex was then loaded onto a HiLoad 16/600 Superdex 75pg column to separate unbound PD-1 from the complex. The complex was then concentrated using a 3K MWCO concentrator.

Crystallization

The Fab-PD-1 complex was crystallized by vapor diffusion at 20°C. The concentrated complex (~15 mg/ml) was mixed 1:1 with reservoir solution comprising 25% PEG 3350, 0.2 M $MgCl_2$, 0.1 M Bis-Tris pH 5.5. Needle-shaped seed crystals generated in this way were vortexed with Seed Beads (Hampton Research) and diluted with reservoir solution. Optimal crystals were obtained after mixing the protein 1:1 with reservoir solution comprising 20.5% PEG 3350, 0.4M $MgCl_2$, 0.1M Bis-Tris pH 5.5, with 20 nl seed solution. Crystals were equilibrated with cryo-protectant 40% PEG 3350 before harvesting.

Diffraction data collection and processing

Single crystals were mounted for data collection, flash-frozen and stored in liquid nitrogen with addition of cryoprotectant. Diffraction data from two crystals were collected at Diamond Light Source Beamline I04, yielding a 98.03% complete dataset to a final resolution of 2.03 Å. Data from individual crystals was integrated using Xia2/Dials. Data collection statistics are reported in Table S2.

Structure determination and refinement

The Fab-PD-1 complex structure was solved by molecular replacement using the program Phaser[72] and the PD-1 monomer (PDB 6k0y) and an Fab (PDB 6cnr) as input models. The asymmetric unit comprised two PD-1 and two Fab molecules. Initial refinement was carried out with REFMAC[73] using maximum-likelihood restrained refinement in combination with the “jelly-body” protocol. Manual model building was performed in Coot.[74] Finally, manual and automatic solvent building with ARP/wARP,[75] was used to produce the final model.

In vitro stimulation (IL-2 and CD69) assays

Thermo Fisher Nunc MicroWell 96-well optical-bottom plates with cover glass bases (glass plates) were wet-coated overnight at 4 °C (for IL-2 assay) and 6 h (for CD69 expression analysis) with DAM polyclonal secondary antibody at 500 μg/mL, or KT3 at the specified concentration diluted in 500 μg/mL DAM antibody, in glass-plate coating buffer (15 mM Na_2CO_3, 11 mM $NaHCO_3$, 0.2% (w/v) NaN_3) and washed three times with sterile PBS. The required number of cells for each cell line was harvested by centrifugation at 396*g* for 5′ and resuspended in appropriate volumes of fresh medium to achieve a density of $1x10^7$ cells/mL. Resuspended cells were then mixed 1:1 with medium containing the test antibodies at the desired concentrations (except for KT3, which had been pre-coated onto the plate; for KT3 stimulation or for the "no stimulation" condition, the cell suspension was mixed 1/2 with fresh medium). Cells were incubated at room temperature for 20-30’. Each condition was performed in technical duplicates or triplicates. To each well was added $5x10^5$ cells in 100 μL of the medium/antibody mixture. Wells were then incubated at 37 °C in 5% atmospheric CO_2 for 24-72 hours for IL-2 ELISA and 0-24 hours for CD69 expression analysis. The cell supernatant was analyzed for released IL-2 using the Mouse IL-2 ELISA Ready-SET-Go! Kit (eBioscience), according to the manufacturer’s instructions. For CD69 expression, cells were lifted off the glass plate by pipetting up and down and moved to a 96-well U-bottom plate. The glass plate was further washed with FACS wash buffer (PBS, 0.05% NaN_3) to capture any residual cells, which were combined with the cells in the U-bottom plate. The cells were then washed once again in FACS buffer and stained with a PE-Cy7 labeled anti-CD69 antibody (Biolegend), a FITC-labeled goat anti-DAM antibody (Bethyl Laboratories), and a dead/alive marker (eFluor780, eBioscience). The “0” time point was an approximation as the cells were added to the plate and then immediately removed but, with washing, this took ~5-10’. CD69 expression and DAM staining was measured by FACS on live cells. All cells were 90% viable except control cells treated with MOPC-21 antibody at 24 hours, which were only ~50% viable.

Calcium release assay

Optical glass-bottom dishes with 10 mm wells were coated with 10-500 μg/ml of antibody as required at 4 °C overnight. For the calcium release assay, BW, Yae5b3k and DO11.10 mouse lymphocytic cell lines were incubated with Fluo-4 as follows: 1×10^6 cells were washed in HBS and placed in a 1:1 mix of RPMI (no supplements) and HBS-probenecid (2.5 μM, pH 7.4) to which 20.8 μg/ml of Fluo-4 dye (final concentration) was added, and the cells left for 20’ at RT. The cells were then washed in HBS-probenecid and resuspended in the same buffer. Cells were then either gently placed onto the antibody-coated dishes directly or resuspended in 10 μg/ml antibody in HBS-probenecid and incubated for 30’, washed, and placed into a secondary antibody-coated dish. Calcium imaging was performed on a spinning disk confocal microscope (Zeiss Group) fitted with a spinning disk unit (Yogokawa Ltd), an AxioCam camera (Zeiss Group), and an incubator to allow imaging at 37 °C. Cells were imaged using a 10x air objective allowing a larger field of view (10^2-10^3 cells). Fluo-4 was excited using a 488 nm laser and the cells were imaged every 1 s. Fluorescence data was analyzed using MatLab-based CalQuo software.[76]

Bilayer preparation

For imaging, bilayers were prepared from a 1 mg/ml SUV solution, typically 95% POPC, 5% DGS-NTA(Ni) (98% POPC, 2% DGS-NTA(Ni) for the experiments shown in Figure 5; Avanti Polar Lipids) on piranha- (overnight) and argon plasma- (30’) cleaned glass slides. After 30’ of incubation with the SUVs, the glass slides were washed three times with PBS before adding purified spacer-His tagged sFcR to the bilayers at a final concentration of 15 μg/ml or, for the PD-1 signaling experiments (Figure 5), a mixture of spacer-His tagged sPD-L1 and sFcR (2.5 μg/ml sPD-L1 and 12.5 μg/ml sFcR). Following a 1 h incubation, the bilayers were washed three times with PBS, and either used immediately or after an antibody solution, i.e., anti-CD28 or anti-PD-1 IgG1 antibody, was added for 15’ at a final concentration of 15 μg/ml. Following the incubation with the antibodies, the bilayers were washed three times prior to addition of the cells. The antibody density was determined using fluctuation correlation spectroscopy and found to be between 100-200 molecules/μm^2. Due to the anticipated low affinity of IgG1 antibody binding to the sFcR, data acquisition was performed within 15’ at room temperature. The cells did not form contacts with SLBs in the absence of antibody.

Imaging T cell contact with glass or bilayers

For imaging T cell contacts with functionalized glass surfaces, $2.5x10^5$ cells in 50 μl RPMI were incubated for 15’ with 50 μg/ml anti-CD28 or anti-PD-1 antibody (labeled with Alexa Fluor 647 N-hydroxysuccinimide (NHS) ester dye conjugate) and 20 μg/ml of the Fab fragment of the anti-mCD45 YW62.3.20 antibody (a kind gift of Prof. Herman Waldmann, Oxford), labeled with the Alexa Fluor 488 NHS ester, for 15’ at 37°C. For the experiments with Jurkat T cells expressing PD-1, Fab fragments of the Gap 8.3 anti-hCD45 antibody, labeled with Alexa Fluor 555 NHS ester dye, were used. Labeling of SHP2 tagged at its C-terminus with a HaloTag (i.e., SHP2Halo), was undertaken using 100 nM Janelia Fluor HaloTag 646 Ligand. Following the antibody and Fab incubation, and cell labeling, the cells were washed three times in PBS prior to imaging on argon plasma cleaned glass slides coated for 15’ with

DAM antibody (at 500 μg/ml). This protocol allowed the imaging conditions to be comparable to the IL-2 stimulation assays. Special care was taken over the degree of labeling of the anti-CD45 Fab. For each set of experiments one preparation of anti-CD45 Fab was labeled with Alexa Fluor 647 NHS ester dye to ensure the degree of labeling remained unchanged for all conditions, for experiments typically lasting three days. For the bilayer imaging experiments, the fluorescently labeled anti-CD28 or anti-PD-1 antibodies were added directly to the bilayers whilst the cells were incubating with the labeled anti-CD45 Fab.

Imaging was performed using a custom-built TIRF microscope with a 100x 1.49NA Nikon TIRF objective. The lasers used for dual-color imaging operated at 488 nm (Spectra-Physics CDHR, 15 mW) and 633 nm (Melles-Griot, HeNe, 10 mW). Three-color imaging utilized 488 nm (Spectra Physics Cyan Laser), 561 nm (Oxxius Laserbox), and 638 nm (Cobolt 06-MLD) lasers. To minimize imaging artefacts arising from uneven illumination of the cells settling onto the functionalized glass surface, the laser illumination was measured using CHROMA autofluorescent plastic slides (CHROMA, 92001) and an area chosen for which the illumination intensity of both lasers differed by less than 5%. The emitted light was split onto an EMCCD camera (512 Delta Evolve, Photometrics) using a DualView2 (Photometrics) imaging system or a filter wheel with the following filters: dichroic, Di03-R405/488/561/635-t1-25x36; 488 nm, FF03-525/50-25; 561nm, BLP01-561R and FF01-587/35; and 647 nm, BLP01-635R-25 (Semrock). Image acquisition was controlled via μManager.[77] Image stacks of 10 (three-color) or 100 (two-color) frames were acquired at 50 ms exposure. Laser intensities were set to ensure no bleaching occurred during the imaging interval. All experiments were conducted during 15’ at room temperature to minimize the effects of the large off-rate of the anti-CD45 Fab. Recorded stacks were averaged prior to analysis.

Image analysis

Following data acquisition, the frames were averaged, and the dark count subtracted. To analyze the data taken on the glass surfaces, the antibody channels were thresholded using intensity-based thresholding. An "antibody mask" was generated using 0.4*maximum intensity, and a 0.6*maximum "high" antibody intensity mask was created to identify regions of high antibody fluorescence. The masks were used to evaluate the CD45 intensity in the two antibody regions as shown in Figure S3. For data taken on bilayers, both channels were background subtracted using rolling ball background subtraction in FiJi (radius = 50 pixel). CD45 masks and antibody masks were then created using thresholds of 0.2*maximum intensity (CD45) and 0.4*maximum intensity (antibody). Once the antibody and CD45 masks were created, they were used to compare CD45 fluorescence intensity "inside the contact ($CD45_{in}$)", i.e., within the antibody mask, or "outside the contact ($CD45_{out}$)", i.e., outside the antibody mask (see Figure S3). The average CD45 intensities in and out of the contact were used to calculate "CD45 exclusion" using the following formula:

CD45 exclusion = 1-($CD45_{in}$/$CD45_{out}$).

For the three-color imaging experiments, the three channels (SHP2Halo, CD45 Fab, and antibody or PD-L1) were averaged and background-subtracted as described above. To compare SHP2 accumulation and CD45 exclusion for each cell, three masks were created: the contact mask (threshold 0.6*maximum intensity of antibody or ligand), and CD45 and SHP2Halo masks (threshold 0.4*maximum intensity). The CD45 and SHP2Halo masks were summed to create a "cell mask". The cell mask was then compared to the contact masks and the CD45 and SHP2Halo intensities inside the contact mask ($CD45/SHP2Halo_{in}$) and outside the contact mask but inside the cell mask ($CD45/SHP2Halo_{out}$) were averaged. The levels of CD45 and SHP2 exclusion were then calculated as follows:

$$\text{CD45 exclusion} = 1\text{-}(\text{CD45}_{in} / \text{CD45}_{out})$$

$$\text{SHP2 exclusion} = 1\text{-}(\text{SHP2Halo}_{in} / \text{SHP2Halo}_{out}).$$

Simulations

Simulations of exclusion and accumulation were generated using Python and the packages NumPy[78] and scikit-image.[79] As ground truth data, a homogeneous intensity of 100 was assumed with a 16 nm pixel size. Circular contacts of user defined size were added. To simulate exclusion one image was generated with intensities 0-100 inside the contacts, to simulate accumulation a second image with the intensity inside the contacts set to 100*set_accumulation was used. For image formation the experimental point-spread function was approximated by a Gaussian blur with sigma 131 nm and pixels binned to a final pixel size of 160 nm. Image brightness was adjusted, and photon shot noise and detection by an EMCCD simulated using a previously published noise model.[80]

Diffusion analysis

For diffusion analysis, BW cells expressing tCD28 tagged with mEos3.2 were allowed to settle onto anti-CD28 functionalized sFcR-presenting SLBs. Images were taken at a frame rate of 54 ms with 5000 frames under weak, continuous 405 illumination (Odicforce), recording mEos fluorescence (using filters BLP02-561R-25 + FF01-580/14-25). SPT was performed using a bespoke SPT code written by Laura Weimann[81] and the tracks subsequently analyzed using Variational Bayes SPT (vbSPT; [82]) to determine the diffusion constants and number of populations, using at least 5000 tracks per data point (usually 1-2 cells). Here, only tracks longer than five frames were selected for analysis.

Surface plasmon resonance methods

All surface plasmon resonance-based assays were performed using a Biacore instrument (model 3000 or T200, Cytiva, as specified) at 37 °C with a running buffer of 10 mM Hepes sodium salt, 150 mM NaCl, 0.005% v/v Surfactant P20 (Cytiva), pH 7.4. To study the kinetics of JJ316 and JJ319 Fab binding to rat CD28, rat CD28Fc was immobilized directly to a CM5 sensor chip in a Biacore 3000 via amine coupling. The reference flow cell was immobilized with an IgG1 isotype control antibody. Antibody Fab was then injected over the two flow cells at a range of concentrations prepared by serial two-fold dilutions, at a flow rate of 30 μL/minute. The sensor chip was regenerated between injections with 5 μL of 10 mM glycine-HCl, pH 2.5 at 10 μL/minute. All data were fitted to a 1:1 binding model using Biacore 3000 Evaluation Software. To determine the kinetics of clone 2 and clone 19 antibody binding to PD-1, each antibody was directly coupled to a CM5 sensor chip in a Biacore T200. The reference flow cell was immobilized with an IgG1 isotype control antibody. Soluble, monomeric PD-1 was then injected over the two flow cells at a range of concentrations prepared by serial two-fold dilutions, at a flow rate of 30 μL/minute. Running buffer was also injected for background subtraction. All data were fitted to a 1:1 binding model using Biacore T200 Evaluation Software. To determine the blocking effects of clone 2 and clone 19 antibodies, PD-1Fc was directly coupled to a CM5 sensor chip in a Biacore 3000. The reference flow cell was immobilized with a IgG1 isotype control antibody; all samples (PD-L1, PD-L2, clone 2 or clone 19) were injected at a flow rate of 5 μL/minute. All binding data were plotted using GraphPad Prism 9.0.1.

Jurkat reporter assay

Experiments were carried out using PD-1 Jurkat reporter cells that express luciferase under an NFAT promoter (Promega), with T cell activation measured by quantification of luminescence. Experiments assessing mouse isotype antibodies were carried out with $5x10^4$ Jurkat cells/well, in a 96 well U-bottom plate, cocultured with $5x10^4$ BW5147 TCS cells expressing an OKT3 antibody-based activating construct with or without hPD-L1 and mFcγR2b, and with PD-1 antibodies or isotype control at the desired concentrations, in a total volume of 80 μL RPMI with 1% FCS. After 6 hours incubation in a humidified CO_2 incubator at 37 °C, plates were removed from the incubator and equilibrated to room temperature for 10'. The amount of luciferase produced was quantified using the Bio-Glo Luciferase Assay System (Promega); 80 μL of Bio-Glo Luciferase Assay Reagent was added to each well and plates were incubated for 10' at room temperature. Luminescence was quantified using a CLARIOstar Plus (BMG Labtech). Experiments involving human isotype antibodies were performed in essentially the same way, except the TCS cells used were HEK293T cells expressing the OKT3 antibody-based activating construct and hFcγR2b and were plated out the day before the assay at $4x10^4$ cells per well in a 96-well flat bottom plate. On the day of assay the medium was removed prior to adding reporter Jurkats and antibodies in a total volume of 80 μL RPMI with 1% FCS.

PBMC in vitro activation assay

PBMCs were isolated from NHSBT NCI leukocyte cones by Ficoll-Paque density gradient centrifugation. $1x10^5$ cells were plated per well in 96 well U-bottom plates, with 0.5 ng/ml soluble anti-CD3 (Biolegend), 0.5 ng/ml anti-CD28 (Biolegend), 20 ng/ml recombinant IL-6 (to upregulate PD-1 expression[83]), 2 μg/ml anti-PD-L1 (Biolegend), and 2 μg/ml anti-PD-L2 (Biolegend; to block baseline signaling through the PD-1 pathway resulting from natural ligand engagement). Anti-PD-1 antibody or isotype control were added to a final concentration of 2 μg/ml, in a total well volume of 200 μl. Cultures were incubated for 72 hours at 37 °C in a 5% CO_2 incubator then supernatants were collected and cytokines assessed using a BD/Th2 Cytometric Bead Array Kit (Becton Dickinson). Five technical replicate wells were carried out per condition per donor for the culture phase, then supernatants from replicate wells were pooled for bead array analysis.

OT-II adoptive transfer assay

Humanized PD-1 mice on the C57BL/6 background were produced by Taconic Biosciences as described in Figure S7A (and Akkaya[40]) and were crossed onto OT-II TCR transgenic mice, which express a TCR specific for an ovalbumin peptide presented by MHC class II,[67] and therefore develop a CD4 T cell compartment greatly enriched for ovalbumin specific cells. The mice were bred to homozygosity for the humanized PD-1 receptor and to heterozygosity for the OT-II transgene (huPD-1_OT-II). Separately, OT-II mice were crossed with C57BL/6 mice that constitutively express a GFP transgene under the control of the ubiquitin C (UBC) promoter (UBC-GFP_OT-II mice). CD4 T cells were purified by negative selection, using the MojoSort Mouse CD4 T cell Isolation Kit (Biolegend), from the spleens of hPD-1_OT-II transgenic mice and UBC-GFP_OT-II mice. On Day 0, these cells were mixed in a 1:1 ratio and 500,000 cells in 200 μl PBS injected intravenously per mouse into syngeneic CD45.1 allotype-marked recipient mice. On Day 1, recipient mice were immunized intraperitoneally with 100 μg ovalbumin in 50% Imject Alum in PBS (v/v). On Day 2, the mice were injected IP with 200 μg of treatment antibody in 200 μl PBS. On Day 8, the recipient mice were humanely killed and splenocytes assessed by flow cytometry. The population of transferred cells was identified as $CD45.2^+CD45.1^-$ and within this population the ratio of humanized (GFP^-) to non-humanized (GFP^+) cells was calculated and normalized to the ratio in the isotype control treated mice.

KLH-induced DTH model

This experiment was performed in 8-16 week old, humanized PD-1 C57BL/6 mice as follows. Day 0: Immunization with KLH/Complete Freund's Adjuvant. The emulsion was a mixture of antigen (KLH, Sigma) in PBS added to Complete Freund's Adjuvant (BD Biosciences) at a ratio of 1:1. The final concentration of KLH was 4 mg/mL and animals were immunized with 100 μL of immunization emulsion injected subcutaneously at 1-2 sites. The unchallenged control group received PBS alone. Day 0: Treatment with

antibodies. Animals were treated, 1 h prior to the immunization, with either mIgG1 isotype control (clone MOPC-21) or anti-PD-1 clone 19 mIgG1 intraperitoneally, at a single dose of 10 mg/kg. The unchallenged control group received PBS alone. Days 0-5: Treatment with cyclosporine A (CsA). Animals in the positive treatment control group were treated by oral gavage with CsA at a dose of 3 mg/kg once per day from Day 0-5. To prepare CsA, Sandimmune Neoral Solution (Novartis) was diluted to 0.3 mg/ml in 0.5% methylcellulose 400cp (Sigma). Day 5: Ear challenge with KLH in PBS. Five days after immunization, mice were challenged in the pinna of the left ear (under anesthetic) with 20 μL of 4 mg/mL antigen solution. The unchallenged control group received 20 μL of PBS in the pinna of the left ear. Day 6: Terminal procedures. Ear thickness was measured using digital calipers. After measuring ear thickness, animals were humanely killed and, postmortem, an 8 mm diameter circle was cut using a biopsy punch from the left and right ear of each animal from all groups. Ears were weighed on a precise analytical balance. Ear oedema was assessed as the difference between left (challenged) and right (control) ear weight.

BM12 transfer model of SLE

The protocol was adapted from the previously described BM12 transfer model.[41] Splenocytes from female humanized PD-1 mice on a C57BL/6 background were passed through a 70 μM nylon cell strainer to obtain a single cell suspension, pelleted and resuspended in PBS, and then transferred into female BM12 recipient mice (The Jackson Laboratory). A total of $4x10^7$ cells, resuspended in 200 μl PBS, were transferred per recipient mouse. A control group received $4x10^7$ splenocytes from syngeneic BM12 donor mice. Antibodies, diluted to 1 mg/ml in PBS were dosed intraperitoneally on Day 1, at 10 mg/kg. A positive treatment control group received dexamethasone from Days 1-35, administered in drinking water. To provide a dose of approximately 1 mg/kg/day, an average daily intake of 0.2 ml/g/day was assumed. Dexamethasone was first reconstituted at 10 mg/ml in 100% ethanol then diluted to 5 μg/ml in drinking water. On Day 35 mice were humanely killed, blood was collected into EDTA tubes, and spleens were removed and weighed using an analytical balance. EDTA tubes containing blood were centrifuged at 2000*g* for 10' to pellet cells and then plasma was pipetted off, diluted 1 in 10 in PBS and stored at -20 °C for subsequent autoantibody ELISAs. Anti-histone ELISA was performed using plates coated with histone from calf thymus (Sigma). Anti-dsDNA ELISA was performed on plates coated with activated deoxyribonucleic acid from calf thymus Type XV (Sigma), using DNA Coating Solution (Thermo Fisher). Goat anti-mIgG (Biolegend) was used to detect total IgG. Plasma collated from all untreated mice was used to produce a standard curve of arbitrary units allowing comparison of interpolated values between groups. Spleens were processed to single cell suspension and assessed by multi-color flow cytometry. Tfh cells were identified as $CD4^+CXCR5^+ICOS^+$ cells rather than using PD-1 as a marker, in case expression or staining of the receptor was impacted by in vivo antibody treatment.

QUANTIFICATION AND STATISTICAL ANALYSIS

Statistical analysis

All statistical analysis was carried out using GraphPad Prism software. Student's t-tests were used to compare two groups of parametric data. When relevant, one-way ANOVA tests were used to compare three or more groups, with Dunnett's multiple comparison follow-up tests performed comparing individual groups to the isotype control treated group. Statistics are included in figures, with a corrected P value of <0.05 defined as significant.

Immunity, Volume *57*

Supplemental information

Antibody agonists trigger immune receptor signaling through local exclusion of receptor-type protein tyrosine phosphatases

Anna H. Lippert, Christopher Paluch, Meike Gaglioni, Mai T. Vuong, James McColl, Edward Jenkins, Martin Fellermeyer, Joseph Clarke, Sumana Sharma, Sara Moreira da Silva, Billur Akkaya, Consuelo Anzilotti, Sara H. Morgan, Claire F. Jessup, Markus Körbel, Uzi Gileadi, Judith Leitner, Rachel Knox, Mami Chirifu, Jiandong Huo, Susan Yu, Nicole Ashman, Yuan Lui, Ian Wilkinson, Kathrine E. Attfield, Lars Fugger, Nathan J. Robertson, Christopher J. Lynch, Lynne Murray, Peter Steinberger, Ana Mafalda Santos, Steven F. Lee, Richard J. Cornall, David Klenerman, and Simon J. Davis

SUPPLEMENTARY INFORMATION

Antibody agonists trigger immune receptor signaling through local exclusion of receptor-type protein tyrosine phosphatases

Anna H. Lippert, Christopher Paluch, Meike Gaglioni, Mai T. Vuong, James McColl, Edward Jenkins, Martin Fellermeyer, Joseph Clarke, Sumana Sharma, Sara Moreira da Silva, Billur Akkaya, Consuelo Anzilotti, Sara H. Morgan, Claire F. Jessup, Markus Körbel, Uzi Gileadi, Judith Leitner, Rachel Knox, Mami Chirifu, Jiandong Huo, Susan Yu, Nicole Ashman, Yuan Lui, Ian Wilkinson, Kathrine E. Attfield, Lars Fugger, Nathan J. Robertson, Christopher J. Lynch, Lynne Murray, Peter Steinberger, Ana Mafalda Santos, Steven F. Lee, Richard J. Cornall, David Klenerman, and Simon J. Davis

Figures S1-S7

Tables S1-S3

Supplementary figures

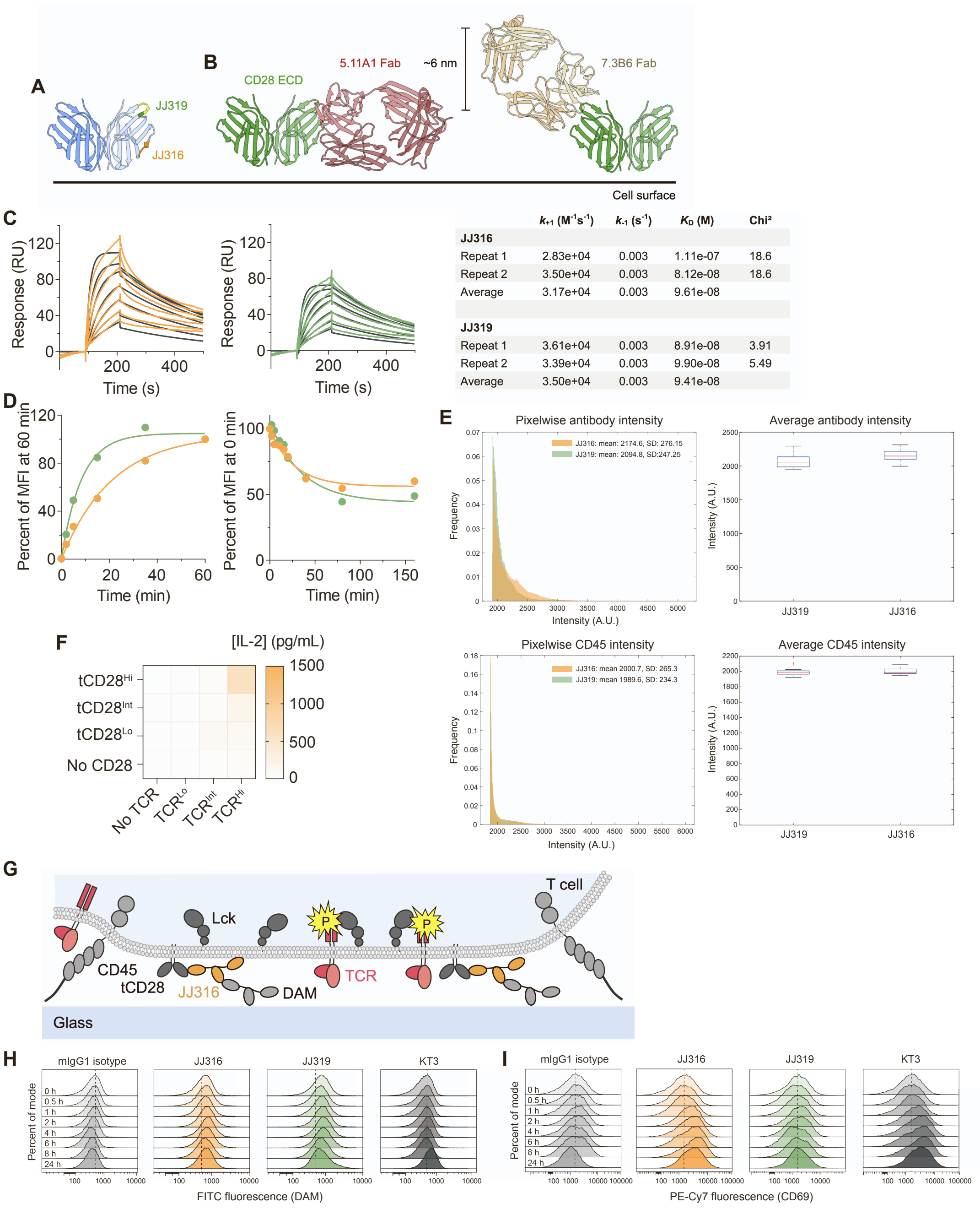

	k_{+1} ($M^{-1}s^{-1}$)	k_{-1} (s^{-1})	K_D (M)	Chi²
**JJ316**				
Repeat 1	2.83e+04	0.003	1.11e-07	18.6
Repeat 2	3.50e+04	0.003	8.12e-08	18.6
Average	3.17e+04	0.003	9.61e-08	
**JJ319**				
Repeat 1	3.61e+04	0.003	8.91e-08	3.91
Repeat 2	3.39e+04	0.003	9.90e-08	5.49
Average	3.50e+04	0.003	9.41e-08	

Figure S1 | Epitopes, binding properties, and signaling effects of anti-CD28 agonistic antibodies, related to Figure 1

(A) The positions of the JJ316 (orange) and JJ319 (green) anti-rCD28 antibody epitopes identified using mutational analysis by Luhder *et al.* [26], relative to the cell surface, shown on the structure of the hCD28 ECD. The JJ316 epitope was identified by substituting residues 60-65 of the C''D loop of mCD28 with the equivalent rat sequence, and the JJ319 epitope via a single substitution (of V98). For reference, the ligand binding MYPPPY motif is shown in yellow.

(B) Crystal structure of the complex of CD28 ECD with the Fab fragment of the 5.11A1 anti-hCD28 antibody, the murine precursor of TGN1412 (adapted from ref. [22]; left). Cryo-electron microscopy [22] showed that a non-mitogenic anti-hCD28 antibody, 7.3B6, bound to the "top" of the CD28 ECD, forming a ~6 nm "taller" complex (measured along an axis orthogonal to the membrane; right).

(C) Example sensorgrams and summary of the affinity and kinetic properties for the binding of Fab fragments of JJ316 and JJ319 antibodies to immobilized rCD28 ECD expressed in the form of a fusion protein with mIgG1 Fc. Measurements were done at 37 °C using a Biacore 3000 machine; the ligand immobilization level was ~300 RU. Analyte concentrations varied from 9.33×10^{-8} to 1.2×10^{-6} M. Black lines in the sensorgrams represent fits of a 1:1 Langmuir binding model to the data.
(D) Flow cytometry-based analysis of the binding of Alexa Fluor 647 labeled antibodies to CD28-expressing BW cells at 37 °C. Data represent averages of three independent repeats. The curves on the left were fitted with an association model: MFI = MFI_{max} * (1-exp(-1*(k_{+1}*L+k_{-1})*time)), where L is the antibody concentration (in M). The curves on the right were fitted with a dissociation model: MFI = (MFI_0 – NS) * exp(-k_{-1}*time) + NS, where NS is non-specific binding at infinite times. The association rates for JJ316 and JJ319 binding to CD28 (3.3×10^5 and 2.9×10^6 $M^{-1}s^{-1}$, respectively) were significantly different (p = 0.0003; F-test) but not the dissociation rates (3.67×10^{-2} s^{-1} and 2.84×10^{-2} s^{-1}; p = 0.3578). Curve fitting was done in GraphPad Prism.
(E) Antibody and Fab labeling levels. Pixelwise and per-cell averaged fluorescence intensities greater than background + 2 SD, for TCR-deficient $tCD28^+$ BW cells incubated with Alexa Fluor 647 labeled JJ316 (orange) or JJ319 (green) antibody, and Alexa Fluor 488 tagged anti-CD45 Fab. Images were taken at the mid-plane of the cells under epi-illumination, following a 15’ incubation with the fluorescent antibodies or Fab. Degree of labeling (dyes/molecule) was 6.9 (JJ316) and 6.4 (JJ319); N = 19 cells (JJ316) and 20 cells (JJ319). Boxes indicate the 25% and 75% quartile, the red horizontal line the median, and whiskers the 1.5 x IQR.
(F) Effects of varying the expression of the TCR and tCD28, a non-signaling form of CD28 lacking its cytosolic region, on signaling by BW cells induced by JJ316 antibody (10 µg/ml).
(G) Proposed mechanism of signaling induced by antibody binding to tCD28 expressed in the presence of high levels of TCR. The formation of a large contact by the binding of immobilized JJ316 to tCD28 leads to exclusion of CD45 over a large area, greatly reducing the likelihood that TCRs constitutively phosphorylated by kinases will be exposed to phosphatases, e.g., CD45, that would otherwise quench signaling.
(H) Time course for staining of cells for DAM adsorption from DAM antibody-coated surfaces during culture for 24 h on these surfaces in the presence of the indicated antibodies at 10 µg/mL, using a FITC-labeled rabbit anti-donkey IgG antibody. The dashed line corresponds to the peak-level staining in the presence of the mIgG1 isotype control.
(I) Staining of cells for CD69 expression following a 24 h culture as in (H), using a PE-Cy7-labeled anti-mCD69 antibody.

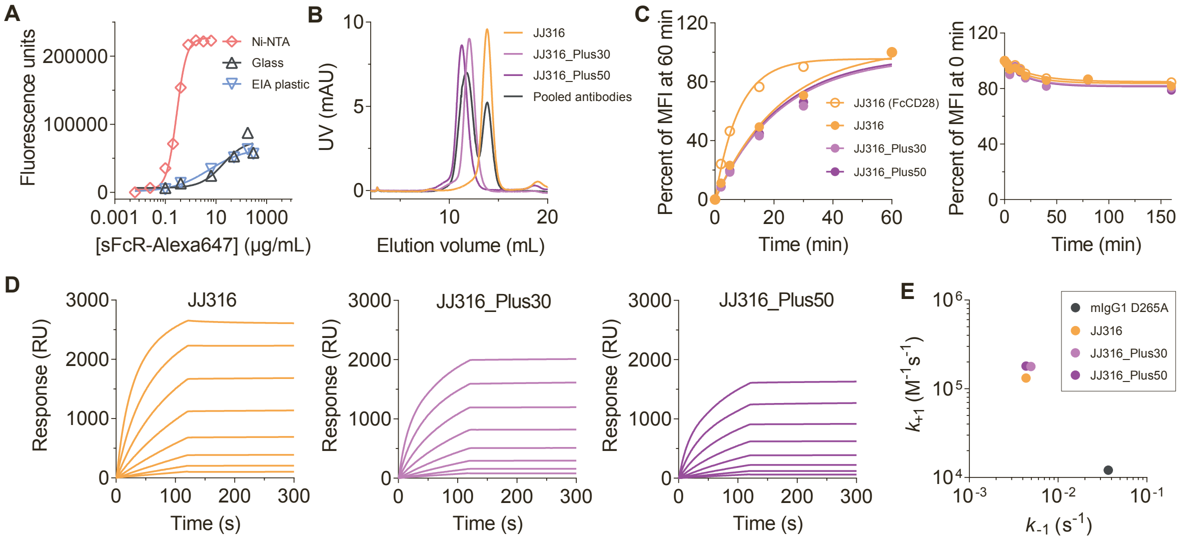

Figure S2 | High-level antibody immobilization and characterization of the effects of extending mIgG1 antibodies with mucin-like sequence, related to Figure 2

(A) Ni-NTA plastic immobilizes His-tagged proteins at very high levels. Histidine (6x)-tagged mFcγR2b ECD was labeled with Alexa Fluor 647 and then allowed to bind to Ni-NTA-coated plastic, or anti-FcR antibody-coated EIA plastic or glass. Fluorescence at 647 nm was used to determine the amount of binding.

(B) Thirty ("Plus30") or 50 ("Plus50") residues of mucin-like sequence from the mCD43 ECD were inserted into the hinge regions of JJ316 antibody. Fifty micrograms of unmodified JJ316, JJ316_Plus30, and JJ316_Plus50 were subjected to size exclusion chromatography on a Sepharose S200 column, either alone or as a mixture.

(C) Flow cytometry was used to follow the binding of the native and hinge-extended Alexa Fluor 647 labeled antibodies to CD28 and FcCD28-expressing BW cells at 37 °C. Data represent averages of three independent repeats. Curves were fitted as in Fig. S1D.

(D) Example surface plasmon resonance-derived sensorgrams showing the binding of JJ316 and extended variants to immobilized rCD28 ECD expressed in the form of a fusion protein with mIgG1 Fc. Measurements were done at 37 °C; ligand immobilization levels were ~3000 RU. Analyte concentrations varied from 1 x 10^{-1} to 7.8 x 10^{-4} mg/ml.

(E) Kinetic analysis of JJ316 and hinge-extended antibody binding to mFcγR2b ECD. Measurements were done at 37 °C; antibodies were immobilized at ~2250 RU. Analyte concentrations varied from 1.60 x 10^{-2} to 6.70 x 10^{-5} mg/ml. Biacore Evaluation Software was used to fit a 1:1 binding model. Calculated on- and off-rates are shown, compared to MOPC-21 mIgG1 D265A used as a negative control.

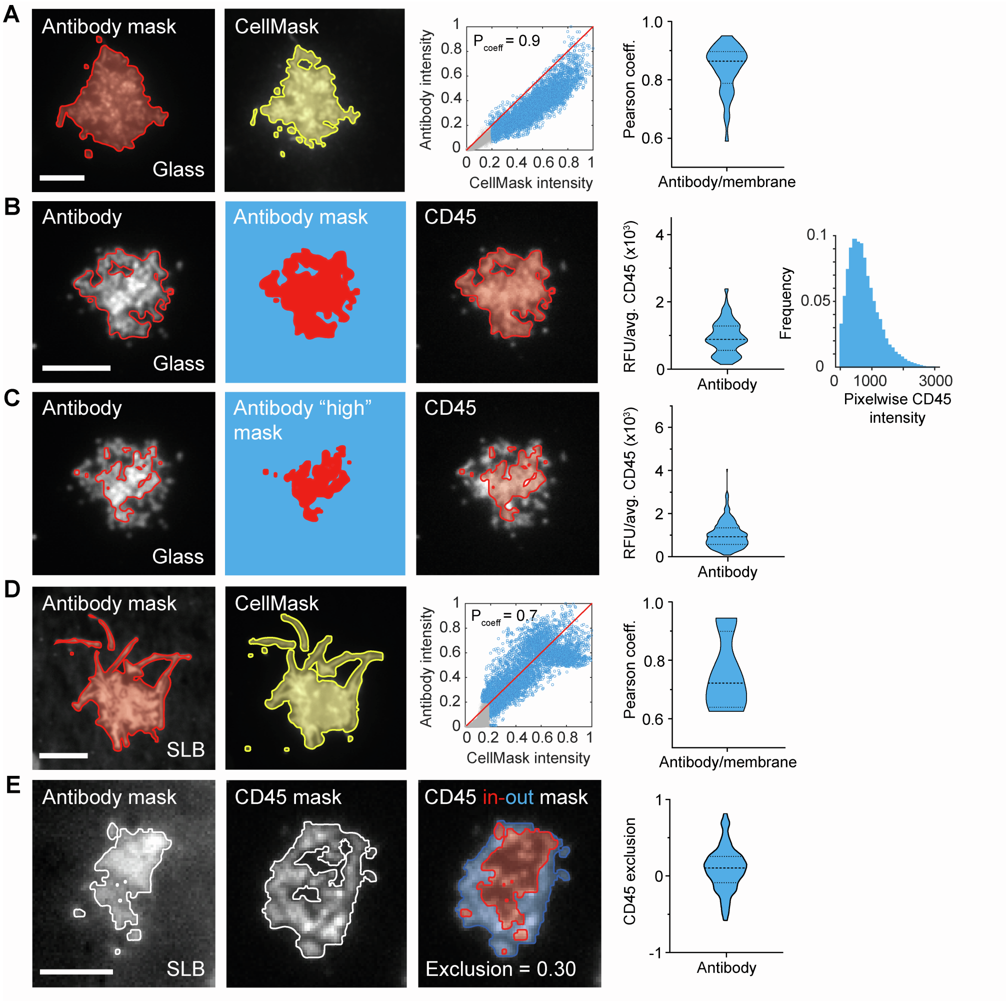

Figure S3 | Fluorescence intensity analysis, related to Figure 3

(A) TIRF images of antibody-treated cells interacting with DAM-coated glass coverslips, following staining with fluorescent anti-CD28 antibody (red) and CellMask (yellow; left panels). Relationship between the pixelwise fluorescence intensities in the antibody and CellMask channels, and correlation calculated using Pearson's coefficient and plotted as a violin plot (right panels; with the violin plot showing 25% and 75% quantiles (dotted lines) and median (dashed line)).

(B) TIRF images of antibody fluorescence for antibody-treated cells interacting with DAM-coated glass coverslips were intensity-thresholded (40% of max. intensity, left panel) to create antibody masks (red, middle panel). These masks were overlayed onto CD45 fluorescence images (right panel) allowing measurements of the average CD45 fluorescence intensity across regions delineated by the antibody masks, presented as violin plots and as histograms of the pixelwise CD45 fluorescence intensities.

(C) Intensity thresholding (60% of max. intensity) was used to also generate masks delineating regions of "high" antibody fluorescence, allowing levels of CD45 fluorescence in these regions to be determined.

(D) Correlation between the pixelwise fluorescence intensities in antibody (red) and CellMask (yellow) channels, for cells interacting with SLBs.

(E) Antibody (40% max. intensity) and CD45 (20% max. intensity) masks (white), for antibody-treated cells interacting with SLBs, were generated by intensity thresholding and overlayed, creating two masks allowing CD45 intensities inside ($CD45_{in}$, red) and outside ($CD45_{out}$, blue) regions of high antibody fluorescence to be measured. Violin plots were used to present cell-wise "exclusion" values (Exclusion = 1 − (Avg $CD45_{in}$/Avg $CD45_{out}$).

Scale bars, 5 µm.

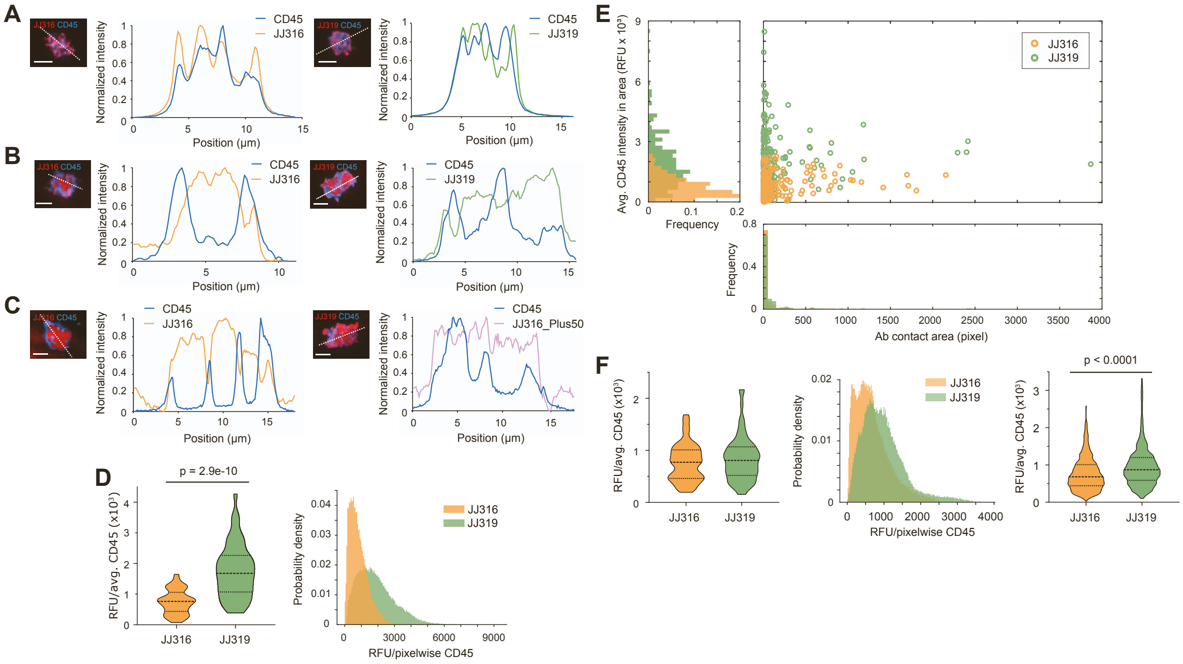

Figure S4 | Fluorescence imaging-based analysis of antibody and CD45 redistribution, related to Figure 3

(A-C) Line scans showing normalized antibody and CD45 fluorescence intensity values from Figure 3B,K, and L in the regions indicated by the dotted lines in TIRF images of tCD28⁺ BW cells treated with Alexa Fluor 647 tagged JJ316, JJ319, or JJ316_Plus50 antibody (red) and Alexa Fluor 488 labeled anti-CD45 Fab fragments (blue). The cells were interacting with DAM-coated coverslips (A) or FcR-antibody presenting SLBs (B,C). Scale bars, 5 µm.

(D) CD45 distribution analyzed using Otsu-based thresholding. Antibody and CD45 masks were created for TIRF images of JJ316- and JJ319-treated tCD28⁺ BW cells interacting with DAM-coated glass surfaces using the Otsu method. Violin plots (left panel) show cell-wise average CD45 intensity values; dotted lines indicate the 25% and 75% quartile and the dashed line the median. The histogram (right panel) shows the probability density function for the pixelwise CD45 intensity for all cells.

(E) Contact size and CD45 exclusion. The scatter plot and histograms show contact sizes and corresponding average CD45 fluorescence intensities for JJ316 (orange)- and JJ319 (green)-interacting tCD28-expressing BW cells.

(F) CD45 distribution for BW cells expressing tCD28 at levels corresponding to that of CD28 expressed by TCR^{Int}-$CD28^{Int}$ BW cells (see Fig. 1F), treated with JJ316 and JJ319 antibody. Violin plots (left panel) compare the average CD45 fluorescence intensities for individual cells (N = 76 cells (JJ316), N = 61 cells (JJ319)). The probability density functions (middle panel) show the pixelwise CD45 fluorescence intensity for all cells. Violin plots (right panel) show the average CD45 intensities in regions of antibody fluorescence. Dotted lines indicate the 25% and 75% quartile and the dashed line the median.

In (D) and (F) two-sample Student's t-tests were used for statistical comparisons.

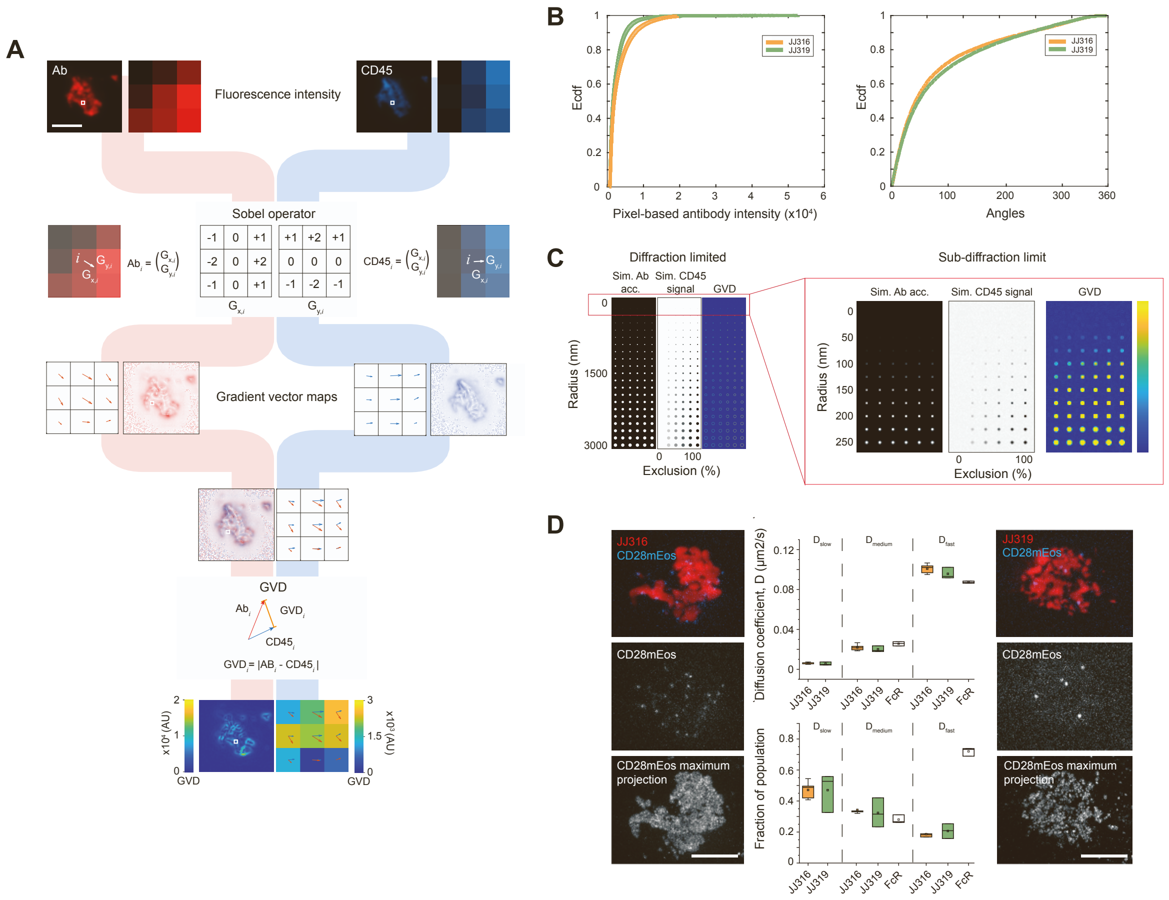

Figure S5 | Gradient vector distance and diffusion analyses, related to Figure 3

(A) For GVD analysis, the CD45 and antibody channels (top) were multiplied with the Sobel operator generating directional gradient vectors Gx and Gy for each pixel (middle). These gradient vector maps were then subtracted from each other, and the GVDs produced were color-coded (bottom). In the top panels, the raw data for each fluorescence channel is shown (left), alongside a subset of 9 pixels (right). For comparisons between two conditions, the fluorescence intensities in the antibody channel were divided by the degree of labeling of the antibody, in order to correct for differences. Scale bar, 5µm.

(B) Sources of differences in the GVD analysis shown in Fig. 3I. The panel on the left shows the empirical cumulative distribution function (Ecdf) for antibody fluorescence intensity, i.e., the pixelwise intensity for values greater than 1.5 x background for antibody intensities measured for JJ316- (orange) and JJ319- (green) treated tCD28^{+} BW cells. The right-hand panel shows the Ecdf of the angles between the vectors assigned to the intensity gradients in the antibody and CD45 fluorescence channels. Degrees of labeling of the antibodies (dyes/molecule) were 9.1 (JJ316) and 7.1 (JJ319).

(C) Simulations show that GVD analysis allows detection of molecular exclusion on sub diffraction-limited length scales. Simulations of antibody accumulation and varying CD45 exclusion, and the resulting GVD plots are shown. The sizes of the contacts and levels of CD45 exclusion were varied. For large regions of accumulation and exclusion, the GVD analysis functioned as an edge detector whereas at sub diffraction-limited length scales, the GVD plot highlights regions of local CD45 versus antibody exclusion. Signal-to-noise ratios were matched to the data shown in Fig. 3I. The simulations were generated on a 16 nm grid assuming homogeneous fluorescence with contacts excluding (CD45) or accumulating (antibody) signal, Gaussian-blurred with a sigma of 131 nm to simulate image formation, and downsampled to a pixel size of 160 nm to match the original data.

(D) CD28 diffusion analysis. BW cells expressing mEos-tagged tCD28 were allowed to settle onto mFcγR2b ECD-presenting bilayers which were preloaded with Alexa Fluor 647 labeled JJ316 or JJ319 antibody. Fluorescence images show antibody fluorescence (red, top) overlayed with a single frame of diffusing CD28mEos (blue, middle) and CD28mEos maximum projection (bottom). Scale bars, 5 µm. Boxplots show diffusion coefficients (upper) and fractions (lower) of slow, medium and fast diffusing populations on antibody presenting and FcR-only bilayers. Data correspond to ~5000 tracks taken from 10 cells each (JJ316- and JJ319-presenting SLBs), or 6 cells (FcR-only SLBs). Boxes indicate the 25% and 75% quartile, horizontal line the median, the square the mean, and whiskers the 1.5 x IQR.

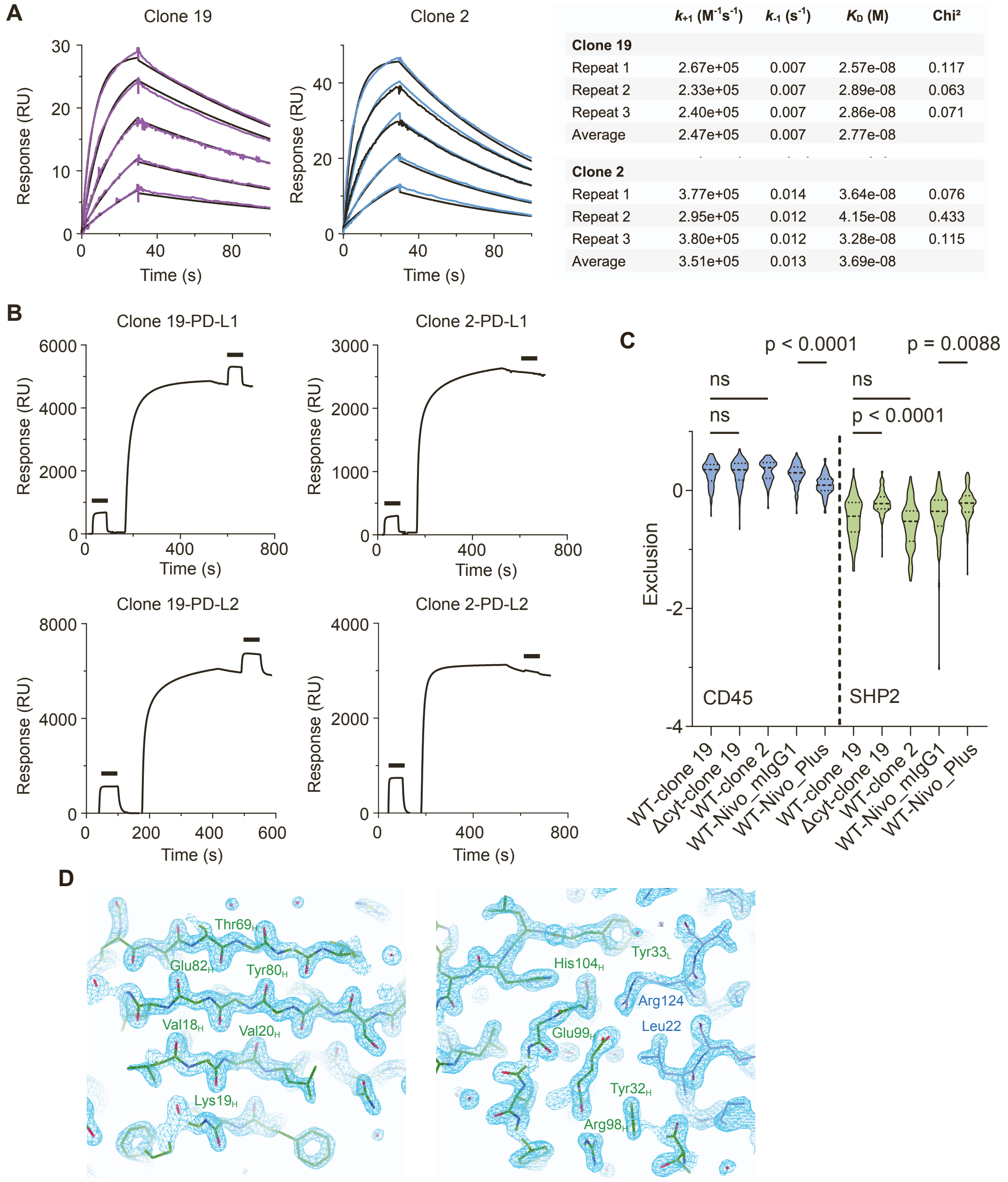

Figure S6 | Anti-PD-1 antibody binding to PD-1, related to Figures 4 and 5

(A) Surface plasmon resonance-based analyses of the affinity of binding of soluble, monomeric PD-1 to directly immobilized clone 19 and clone 2.

(B) Analysis of the blocking effects of the anti-PD-1 antibodies. In the blocking experiment, PD-1Fc was directly immobilized to a Biacore T200 sensor chip, into which the indicated ligands, PD-L1 or PD-L2 (0.35 mg/ml), were injected (first black bar) in order to establish the levels of ligand binding before addition of the anti-PD-1 antibodies. Note that the ligands completely dissociated before injection of the antibodies. The clone 19 or clone 2 antibodies (0.1 mg/ml) were then injected into the sensor chip, giving large, close-to-saturating amounts of binding. In a final step, the ligands were re-injected (0.35 mg/ml; second black bar) in order to observe the amounts of PD-L1 and PD-L2 binding in the presence of the antibodies. The absence of an increase in signal during the second ligand injection step indicated that clone 2, but not clone 19, blocked ligand binding to PD-1.

(C) Violin plots of CD45 (blue) and SHP2 (green) mask-based exclusion values comparing different antibody-mediated contacts: WT-clone 19 (N = 101 cells), WT-clone 2 (N = 110 cells), WT-Nivo_mIgG1 (N = 78 cells), WT-Nivo_Plus (N = 83 cells), and PD-1Δcyt-clone 19 (N = 122 cells), for cells expressing either PD-1WT (WT) or PD-1Δcyt (Δcyt), on bilayers comprising 95% POPC, 5% DGS-NTA(Ni), rather than 98% POPC, 2% DGS-NTA(Ni) as used in the experiments shown in Fig. 5, giving higher levels of antibody immobilization. The Kruskal-Wallis test with Dunn's multiple comparison follow-up testing was used to compare each group to the WT control.
(D) Representative regions of electron density used in modeling the clone 19 Fab-PD-1 structure. Section of the V_H DEBA β-sheet of the clone 19 Fab (left panel), and PD-1 (blue) and clone 19 Fab (green) interface resides (right panel; mature polypeptide numbering). The $2F_{obs}–F_{calc}$ maps (blue mesh) were contoured at 0.7 sigma.

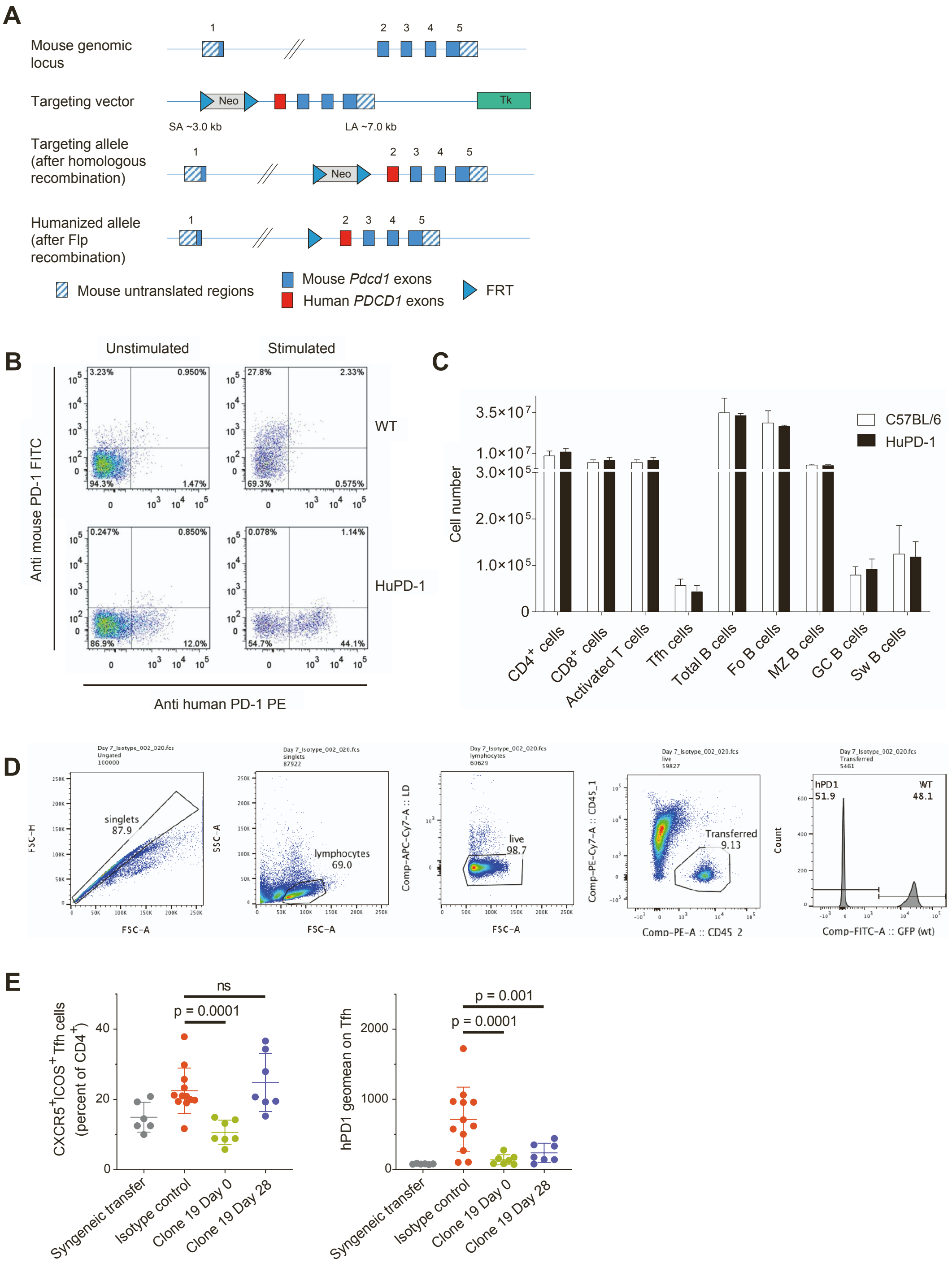

Figure S7 | In vivo experiments, related to Figure 6

(A) Strategy for generation of the humanized allele in huPD-1 mice (adapted from ref 40). Murine *Pdcd1* exon 2, encoding the ECD of PD-1, was replaced by the human counterpart using targeted homologous recombination by electroporation of mouse C57BL/6N ES cells. Microinjection of blastocysts with positive ES clones was performed with implantation of blastocysts into foster mice. Chimeric offspring were bred to FLP transgenic mice and offspring with germline transmission of the knockin, plus somatic deletion of the frt flanked neomycin resistance cassette, were identified. Mice were bred to homozygosity of the humanized allele, with genotyping performed by PCR of digested ear punch samples with primers specific for *PDCD1* (human) or *Pdcd1* (mouse) exon 2.

(B) Flow cytometry plots showing upregulation of the humanized PD-1 protein on T cells upon in vitro stimulation. Splenocytes from WT C57BL/6 mice and homozygous huPD-1 mice were stimulated with 1 µg/ml anti-CD3 antibody. Cells were stained with FITC conjugated anti-mPD-1 and PE conjugated anti-human PD-1 antibodies. Weak expression of PD-1 by splenic T cells in the resting state (versus, e.g., PBMCs), was expected.
(C) Comparison of the naïve status of huPD-1 and C57BL/6 immune systems. Quantification of immune cell subsets in spleen by flow cytometry, gating for specific populations, was as follows: activated T cells ($CD3^+CD44^+ICOS^+$), Tfh ($CD4^+ICOS^+CXCR5^+$), Fo B cells ($B220^+CD23^{hi}CD21^{int}$), MZ B cells ($B220^+CD23^{lo}CD21^{hi}$), GC B cells ($B220^+CD95^+GL7^+$), and Sw B cells ($B220^+IgG^+$). Data were pooled from three independent experiments with at least two mice per group (mean+/-SD).
(D) Flow cytometry gating strategy for the OT-II adoptive transfer study. Transferred versus recipient cells were separated based on the CD45 allelic marker, and huPD-1 versus WT PD-1 expressing cells within the transferred population were separated based on GFP expression.
(E) Evidence that clone 19 IgG1 is a non-depleting antibody. Fraction of $CD4^+$ T cells expressing CXCR5 and ICOS, i.e., Tfh cells, in mice on Day 30 following treatments on Days 0 or 28 with 10 mg/kg clone 19 IgG1 anti-PD-1 or isotype control antibody, in the setting of the SLE model (left panel, see Fig. 6). Levels of expression of PD-1 by the Tfh cells (right panel). Treatment with clone 19 for 2 days, i.e., the time during which depletion by CDC-ADCC would occur, leads to downregulation of PD-1 only, confirming that the receptor has been engaged and that the antibody is non-depleting. Each point represents an individual mouse. Error bars shown represent SD. Data are representative of two independent experiments. One-way ANOVA with Dunnett's multiple comparison follow-up testing comparing each group to the isotype control, was used for statistical comparisons.

Supplementary tables

Table S1 | Non-linear regression/binding model*-based analysis of the agonistic and costimulatory activities of JJ316 and JJ319 antibodies, related to Figures 1 and 2

	**No anti-CD28**	**Isotype**	**JJ316**	**JJ319**
**DO11.10**				
**Maximum**	Constrained	Constrained	Constrained	Constrained
**n**	0.9894	0.945	0.8109	0.8006
**EC50 (µg/mL)**	28.64	32.25	213.8	1.492****
**Minimum**	-9.275	-6.292	654.4†	22.4
**Yae5b3k**				
**Maximum**	Constrained	Constrained	Constrained	Constrained
**n**	1.383	1.301	0.8145	0.6415
**EC50 (µg/mL)**	29.82	20.67	66.94	2.762**
**Minimum**	-2.176	1.455	202.1***	9.15
**TCR^+ BW**				
**Maximum**	722.9	890.6	309.4	833.3
**n**	0.8151	0.4672	2.398	3.553
**EC50 (µg/mL)**	1.888	3.312	0.008186	0.009791‡
**Minimum**	-2.135	-28.46	529.7§	6.886

* Model: [IL-2] = Maximum * [KT3]n/(EC50n + [KT3]n) + Minimum; where Maximum = [IL-2] at maximal [KT3]; EC50 = [KT3] that gives half-maximal [IL-2]; n = Hill slope; Minimum = [IL-2] at [KT3] = 0
** Different from isotype, p = 0.0023 (F-test)
*** Different from isotype, p < 0.0001 (F-test)
**** Different from isotype, p < 0.0001 (F-test)
† Different from isotype, p < 0.0001 (F-test)
‡ Different from isotype, p < 0.0001 (F-test)
§ Different from isotype, p = 0.0002 (F-test)

Table S2 | Data collection and refinement statistics for the PD-1-clone 19 Fab crystal structure, related to Figure 5

Space group	P 1
Unit cell	a = 52.16 Å, b = 53.79 Å, c = 103.00 Å, α = 104.80°, β = 101.83°, γ = 92.57°
Wavelength (Å)	0.979499
Resolution range (Å)	51.85 - 2.03 (2.06 - 2.03)
Completeness (%)	98.03 (95.11)
Multiplicity	3.63 (3.71)
CC-half	0.9918 (0.2220)
I/sigma	11.11 (1.07)
Rmerge(I)	0.1322 (1.2855)
Anomalous completeness (%)	96.89 (92.69)
Anomalous multiplicity	1.82 (1.88)
Reflections used in refinement	66773 (6591)
Reflections used for R-free	3086 (304)
R-work	0.2141 (0.3314)
R-free	0.2725 (0.3511)
Number of non-hydrogen atoms	8443
Macromolecules	8166
Ligands	28
Solvent	249
Protein residues	1058
RMS (bonds)	0.015
RMS (angles)	2.08
Ramachandran favored (%)	96.13
Ramachandran allowed (%)	3.29
Ramachandran outliers (%)	0.58
Rotamer outliers (%)	6.80
Clash score	6.44
Average B-factor	35.84
Macromolecules	35.97
Ligands	61.24
Solvent	28.84

Table S3 | Comparison of the interfaces formed with PD-1 by anti-PD-1 antibodies revealed by crystal structural studies, related to Figure 5

		Clone 19 (PDB: 8eq6)	Nivolumab (PDB: 5WT9)	Pembrolizumab (PDB: 5B8C)
**Buried area (Å^2)**	V_L	288.7	290.6	551.8
	V_H	484.2	571.3	662.1
	PD-1	781.5	926	1170.9
**No. of contacting residues**	V_L	5	4	11
	V_H	11	12	16
	PD-1	15	11	23
**No. of charged contacting residues**	V_L	0	0	2
	V_H	3	3	2
	PD-1	5	3	6
**No. of polar contacting residues**	V_L	5	2	7
	V_H	8	11	11
	PD-1	10	6	13
**No. of hydrophobic contacting residues**	V_L	3	4	8
	V_H	9	6	11
	PD-1	8	5	11
**No. of aromatic contacting residues**	V_L	3	1	4
	V_H	5	3	4
	PD-1	0	0	2
**Shape complementarity *(Sc)*[1]**		0.72	0.81	0.68

Supplementary reference

1. Lawrence, M.C., and Colman, P.M. (1993) Shape complementarity at protein/protein interfaces. J. Mol. Biol. *234*, 946-950.
